# Impact of a pain education program for people with spinal cord injury who experience neuropathic pain

**DOI:** 10.3389/fpain.2025.1569446

**Published:** 2025-05-27

**Authors:** Nicholas P. Cherup, Kimberly D. Anderson, Marlon L. Wong, Gabriel E. Fernandez, Linda E. Robayo, Kathryn Roach, Roberta Vastano, Eva Widerstrom-Noga

**Affiliations:** ^1^ The Miami Project to Cure Paralysis, Miller School of Medicine, University of Miami, Miami, FL, United States; ^2^ Department of Physical Medicine and Rehabilitation, MetroHealth System, Case Western Reserve University School of Medicine, Cleveland, OH, United States; ^3^ Department of Physical Therapy, Miller School of Medicine, University of Miami, Miami, FL, United States; ^4^ Department of Neurological Surgery, Miller School of Medicine, University of Miami, Miami, FL, United States

**Keywords:** spinal cord injury, neuropathic pain, qualitative interviews, pain education, pain outcomes

## Abstract

**Background:**

Neuropathic pain is common after spinal cord injury (SCI). Despite the availability of various treatments, many report inadequate pain relief, and various side effects.

**Objective:**

The primary purpose of the current study was to explore participants’ perspectives on a brief, four-week virtual pain education program and second to evaluate any effects on pain and psychosocial factors.

**Methods:**

This study included 36 participants with SCI who experienced moderate to severe neuropathic pain and explored their perspectives on the pain program using qualitative interviews and evaluated a small set of self-reported pain outcomes.

**Results:**

The analysis and coding of the qualitative interview data resulted in two primary overarching themes: *Benefits of pain education and Content and delivery of pain education.* The *Benefits of pain education* theme was further analyzed and divided into 6 subthemes: Learning about pain and treatment options in general, Learning from and interacting with peers, Learning about non-pharmacological approaches and ways to self-manage pain, Learning about pathophysiology of pain, Learning about pain medication, and Improving communication about the lived experience with pain. Under the main theme of *Content and delivery of Pain Education,* there were three subthemes: Positive, No effect or negative, and Change suggestions. Specifically, participants reported having a better understanding about treatment options, how their peers managed their pain, and the underlying causes and types of pain. Participants also perceived that this knowledge would improve their ability to talk to others about their pain. Participants mentioned the topics discussed and the small group interactive settings as positive aspect of the education, although some did not benefit or felt that focusing on pain made pain more obvious to them. The overall benefit was consistent with small but significant improvements in perceived pain interference with daily activities and difficulty in dealing with pain (*p* < 0.05).

**Conclusion:**

Overall, these findings suggest that a brief, virtually administered pain education program in a small group setting may be a positive addition to an interdisciplinary pain program. Future research should continue to develop and individually tailor such programs in this population, as these approaches are low-cost and easily accessible.

## 1 Introduction

Approximately 80% of those living with spinal cord injury (SCI) develop chronic pain during their first year of recovery (1–3). Pain that persists despite the implementation of available treatments can significantly interfere with sleep, exercise, work, and daily activities (4); all of which progressively compromise physical and mental health while decreasing self-reported quality of life (5). Qualitative studies have highlighted several important clinical concerns regarding pain's significant and negative impact on day-to-day activities (6, 7) with research indicating multiple areas of interest that require attention in the long-term care of those living with SCI. For example, individuals who experience chronic pain emphasize the need to obtain better information about pain and existing treatment options, including non-pharmacological approaches during both acute and chronic injury stages (8), having access to healthcare professionals with expertise in pain management (6, 7, 9), and learning how to develop pain acceptance and a better sense of control over their symptoms (10). These sentiments may be especially crucial for those living with unmanageable pain when compared to those who have acquired some effective pain management strategies.

The information and identified care needs are consistent with the principles of patient-centered multimodal treatment approaches and are critical to those with neuropathic pain since most pain symptoms will persist long after their SCI (1, 3, 11). Additionally, the requirements of those living with comorbid pain syndromes vary greatly — further emphasizing the clinical reality that what may work for one, may not work for all. In a previous mixed-methods study conducted by our research group, we found three different subgroups of people living with SCI-associated neuropathic pain (12). The first subgroup experienced considerable pain impact, pain interference, and affective distress, as well as low levels of life control after their SCIs. They considered obtaining information about pain and pain management options (including non-pharmacological options) to be of high interest throughout their recovery (acute, subacute, and chronic) to enable them to best understand and deal with their pain over time. Individuals in this group also felt that being able to communicate about pain and having access to knowledgeable healthcare providers were high priorities. A second subgroup was characterized by moderate pain and high resilience. These individuals considered pain information important; however, compared to the other subgroups, they were more concerned with side effects associated with pain medication use and the potential risk for addiction. Finally, a low-pain impact group was identified and included a subset of individuals who expressed less interest in current information about pain, its treatments, and the need for increased communication regarding their symptoms. Such findings illustrate the unique needs of those living with SCI-associated neuropathic pain and suggest that individuals with more severe pain and greater self-reported pain interference may benefit from learning more about their condition and the most effective strategies to ease pain's influence over their daily lives. Indeed, the severity of neuropathic pain after SCI has been shown to improve when pain education is integrated into a multidisciplinary pain management program (13). Therefore, it is likely that effective management of SCI-associated neuropathic pain may be influenced by increasing access to condition-specific information. Moreover, improving overall health literacy (14), can only help to facilitate informed decision-making processes involved with an individual's pain management program.

The present study consisted of a subset of data from a larger mixed-methods 12- week pilot study including 4 weekly pain education small virtual group sessions that were followed by a series of exercise, and bodily illusions sessions, and a 4 week follow up. The present article focused on the pain education component which was completed before the other sessions. The pain education was based on a written educational resource, the *SeePain*, which is publicly available as Supplementary Material in our recent publication (15). Both patient perceptions and their lived experience represent important, yet at times overlooked, components of the pain management process; thus, the main purpose of this mixed method study was to explore participants’ perspectives on the pain education sessions using qualitative interviews. The secondary aim of this study was to evaluate any effects on pain and psychosocial factors.

## 2 Methods

### 2.1 Research design

The current study was part of a larger mixed-methods qualitative 16-week study that included four weeks (1 h each, administered weekly) of pain education followed by 6 weeks of biweekly upper body exercise combined with visual illusions, and a 4-week follow-up. The current manuscript focused on the data that was obtained via qualitative interviews after completed pain education and before other study activities, and outlines participants' perceptions about their engagement in the four-week pain education sessions. Each participant also completed a pain assessment at baseline and after the four educational sessions.

### 2.2 Study participants

Study participants were recruited via flyers posted at the Miller School of Medicine and from The Miami Project's research volunteer database. All participants went through a screening and an informed consent process and provided written consent after confirmed eligibility. The study adhered to the principles of the Declaration of Helsinki and was approved by the Institutional Review Board of the University of Miami Miller School of Medicine and the Department of Defense Office of Human and Animal Research Oversight.

The sample (*N* = 36) consisted of English-speaking men and women with complete or incomplete SCI, who were between 18 and 75 years of age, and who had also experienced moderate to severe neuropathic pain for a minimum of 6 months [numeric rating scale (NRS) ≥ 4/10]. See Table 1 for pain and demographic information. Potential participants with a history of systemic illness (e.g., cardiovascular disease, multiple sclerosis, rheumatoid arthritis, cancer), severe depression (BDI-II > 29), body mass index (BMI) > 35, unhealthy alcohol (AUDIT > 10) or drug (DAST-10 > 6) use within the past year, were not eligible for the study.

**Table 1:** Demographic and injury characteristics (*N* = 36).

Characteristic	Category	Summary Statistic
Worst neuropathic pain type	At-level	17 (47.2)
(*n*, %)	Below-level	19 (52.8)
Worst neuropathic pain intensity		
Mean, SD		7.1 (2.2)
Age (years, mean, SD)	-	42.3 (14.0)
Sex (*n*, %)	Male	27 (75.0)
	Female	9 (25.0)
Ethnicity (*n*, %)	White non-Hispanic	8 (22.2)
	Hispanic	13 (36.1)
	African American	8 (22.2)
	Asian	1 (2.8)
	Other	6 (16.7)
Marital Status (*n*, %)	Single	25 (69.4)
	Married	6 (16.7)
	Divorced/separated	5 (13.9)
Education (*n*, %)	Pre-High School	4 (11.1)
	High School	9 (25.0)
	Trade School	1 (2.8)
	AA or some college	14 (38.9)
	Bachelor's	4 (11.1)
	Advanced Degree	3 (8.3)
	Other	1 (2.8)
Employment (*n*, %)	Employed Full Time	4 (11.1)
	Employed Part Time	1 (2.8)
	Unemployed	16 (44.4)
	Self-Employed	6 (16.7)
	Disability	4 (11.1)
	Retired	3 (8.3)
	Student	2 (5.6)
Age at Injury (years)		
mean (SD), range (min, max)	-	32.4 (13.3) 52 (16, 68)
Time since Injury (years)		
Mean (SD) range (min, max)	-	10.0 (9.9) 38.5 (0.5, 39)
Type of Injury (*n*, %)	Tetraplegia	20 (55.6)
	Paraplegia	16 (44.4)
Completeness (*n*, %)	Complete	11 (30.6)
	Incomplete	25 (69.4)
Cause of Injury (*n*, %)	MVA	18 (50.0)
	Fall	7 (19.4)
	Sporting Accident	4 (11.1)
	Acts of Violence	4 (11.1)
	Other	3 (8.3)

### 2.3 Screening measures

#### 2.3.1 Beck depression inventory, 2nd edition (BDI-II)

The BDI-II is a 21-item self-report multiple choice questionnaire designed to assess depressive symptoms consistent with the DSM-IV (16, 17). Participants were asked to rate their symptoms over the past two weeks on a 4-point scale from 0 to 3, with overall scores ranging from 0 to 63. Range of depression: 0–13 minimal, 14–19 mild, 20–28 moderate, and 29–63 severe. The internal consistency and reliability of the BDI-II among those with chronic pain conditions has been previously established (18).

#### 2.3.2 Alcohol use disorder identification test (AUDIT)

The AUDIT (19) provides an accurate measure of risks associated with overconsumption of alcohol. The AUDIT consists of 10 items about alcohol use, alcohol dependence symptoms, and alcohol-related problems over the past year. Psychometric properties of the AUDIT have been discussed elsewhere (20).

#### 2.3.3 Drug abuse screening test (DAST-10)

The DAST-10 (21) is designed to be used in a variety of settings to provide a simple way to detect drug-related problems over the past year. The DAST includes 10 items rated on a yes/no binary. The DAST-10 is psychometrically consistent and reliable among various populations (22).

### 2.4 Pain education sessions

The one-month educational program consisted of four separate Zoom sessions. Each 60-minute session included a Power point presentation (Supplementary Materials) and was led by one or several members of the study staff. The sessions were hosted by different members of the SCI pain research team but most commonly by NC, LR, or GF. Two were postdoctoral associates trained in interdisciplinary SCI pain research and in interaction with research participants with SCI who experienced neuropathic pain. All were trained regarding the content of the pain education sessions. The PowerPoint presentations were based on the recently published pain education resource developed by our laboratory entitled the *SeePain* which is publicly available as Supplementary Material in our recent article (15). The *SeePain* features basic information about the classification and chronicity of neuropathic pain, real case examples, and different ways to manage symptoms, including self-management strategies, pharmacological approaches, and non-pharmacological techniques. Each session was conducted in a small group format consisting of 2–6 participants. While specific content was covered in each respective session to provide consistency, the group facilitator continuously encouraged questions and solicited those in attendance to share their personal pain management experience. Participants stayed with the same group for all 4 group sessions. Participants were enrolled consecutively and not based on specific injury characteristics, pain presentation, coping capabilities, cultural background etc. Participants were encouraged to share details about their pains and how they managed it. Some had not discussed this with other people before. However, if the group facilitators noticed that a participant did not engage or seemed shy, they tried to facilitate engagement by asking that person questions and encouraging their participation. As a rule, the educational sessions were scheduled on the same weekday day and time to facilitate participation. For a more detailed outline of the content covered in each weekly session, please see Table 2 below and Supplementary Material.

**Table 2:** Educational outline for each zoom session.

Session	Topic	Specific Content Covered
1	Basic Pain	Describe the components of the central nervous system affected by SCI, the difference between nociceptive and neuropathic pain types, how such pain is differentially classified and experienced after SCI, and relevant pain mechanisms.
	Mechanisms	
2	Real-world Case Examples	Provide examples of real cases describing the pain relative to level of injury, the experience, and the location of different types of pain. In addition, there was a discussion among participants regarding the life domains that may be impacted by pain.
3	Pharmacological Treatment	Overview of pharmacological treatments, including current recommendations, side effects, and potential drug interactions.
4	Non-pharmacological Treatment	Overview of non-pharmacological modalities including multiple non-pharmacological treatments (e.g., non-invasive stimulation, exercise, mindfulness, and various self-management strategies).

### 2.5 Qualitative interviews

After the completion of all the education sessions, each participant took part in a qualitative Zoom interview on a 1 to 1 basis conducted by MW who did not have any other interactions with the study participants. During each session, participants were asked a predetermined set of open-ended questions based on an interview guide to facilitate uniformity across interviews. The interview questions (see examples below) were designed to expand participants' perspectives about the pros and cons of the pain education and their overall thoughts and feelings about each session.

• Please describe your experience with the pain education part of the study.
• What did you like about it?
• What did you not like about it?
• What were the benefits, if any, of pain education?
• What were the risks or burden, if any, of pain education?
• What impact, if any, did it have on your ability to manage your pain?

### 2.6 Quantitative measures

Before and after the educational sessions, we conducted the following assessments:

#### 2.6.1 The international SCI pain basic dataset (ISCIBPD-2)

The ISCIBPD-2 (23) is part of the NIH SCI Common Data Elements and provides a pain classification and overall description of all pain experienced. For the purposes of the present study, we used the three pain interference items (Interference with activities, mood, and sleep), rated on a numerical rating scale ranging from 0 = no interference to 10 = extreme interference, and the worst pain intensity rating ranging from 0 = no pain to 10 = pain as bad as you can imagine.

#### 2.6.2 Difficulty in dealing with pain

Participants provided ratings for overall difficulty in dealing with pain on an NRS from 0 (not hard at all) to 10 (extremely hard) (24). Global ratings of difficulty in dealing with chronic pain and other consequences of injury have previously been used in the SCI population (25).

#### 2.6.3 Psychosocial

The SCI version of the Multidimensional Pain Inventory (MPI-SCI) was used in the current study to assess the psychosocial impact of pain. The West Haven-Yale Multidimensional Pain Inventory (MPI) (26) is a comprehensive instrument designed to assess a range of self-reported behavioral and psychosocial factors associated with chronic pain syndromes. The MPI-SCI consists of 50 items that are answered on a 7-point Likert scale Ranging from 0 to 6) and is a valid and reliable measure among those with SCI (27). For the purposes of the present study we used the 5 subscales: Pain severity, Life Interference, Affective Distress, Life Control, and Social Support.

### 2.7 Data analysis

#### 2.7.1 Thematic analysis

The target sample size was determined using recommendations set forth by prior studies utilizing grounded theory (28). All interviews were conducted via Zoom and were recorded and transcribed verbatim by an independent transcriber. The study staff also completed quality control procedures to ensure data accuracy. This included listening to the audio files and comparing it to the transcripts and correcting any errors in the transcripts including removing identifiers. The final transcribed documents were uploaded to NVIVO (software headquartered in Lumivero, Denver, CO). Two independent researchers (EW and KA) used an iterative open coding comparative process. All content was summarized into themes, revised and confirmed. The themes obtained from the reviewers' thematic analyses were subsequently discussed in study team meetings and revised if appropriate. Given the narrow scope of our study, we reached saturation (no new themes emerged) at 30 SCI participants. Therefore, we completed our interviews with 36 individuals with SCI-associated neuropathic pain.

#### 2.7.2 Quantitative analyses

Pain-related variables were assessed for normality and homogeneity of variance using Shapiro–Wilk tests indicating non-normality. Median scores for ISCIBPD-2 worst pain intensity, and pain interference with activities, mood, and sleep, difficulty dealing with pain and MPI sub-scales (Pain Severity, Life Interference, Locus of Control, Affective Distress, and Social Support) were compared between baseline and after the educational sessions using Wilcoxon Signed Ranks Tests. All statistical analyses were conducted using Statistical Package for the Social Sciences (SPSS) v28 (IBM Corp., Armonk, NY). Results were considered significant if values met the *a priori* threshold set at *p* ≤ 0.05.

## 3 Results

### 3.1 Thematic analysis

Results and representative quotes emerging from the thematic analysis are presented below. With respect to the overall perceptions regarding the pain education sessions, two main themes emerged, i.e., *Benefits of pain education*, and *Content and delivery of pain education. Benefits of pain education,* was further analyzed and divided into 6 subthemes: (a) Learning about pain and treatment options in general, (b) Learning from and interacting with peers, (c) Learning about non-pharmacological approaches and ways to self-manage pain, (d) Learning about pathophysiology of pain, (e) Learning about pain medication, and f. Improving communication about the lived experience with pain. Under the main theme of *Content and delivery of pain education*, there were 3 subthemes: (a) Positive, (b) No effect or negative, and (c) Change suggestions. All themes are arranged below in descending order of endorsement with a few representative quotes relative to number of quotes.

#### 3.1.1 Benefits of pain education:

a. Learning about pain and treatment options in general

Under this theme participants described the general perceived benefit of the pain education sessions. They valued having access to more information regarding pain associated with SCI and its management. Some participants mentioned that the education sessions made them reevaluate their SCI and pain and search for more information, while others expressed that they would have appreciated getting this information while in the hospital or from their providers. Overall, they expressed that having a better understanding of their pain was a benefit itself.

Participant 11: *I've had a spinal cord injury for 10 years and I've always wondered why or how is this pain happening? Because I would go to the hospital and they wouldn't even know. So, it was very informative. It could be nerves, it could be the way you're sitting, it could be the temperature, and such things like that.*

Participant 16: *There was a few things popped up that I've heard of or what I'm familiar with, but it was good to hear them within that session…. It was nice. And so it got me thinking some things, so I read some stuff.*

Participant 19: *So, it's learning things that I never thought about, but it's also helping me live my life with the knowledge of what happened to me and what is going on in my body.*

Participant 23: *It gave me a totally different perspective on pain, spinal cord injuries, and my own spinal cord injury. Made me think about myself a little differently, which was really cool.*

Participant 27: *You don't really want to study bad news like that, but in a way, this don't look at it as bad news. It's like a way of life. You're just being informed of… the nature to your injury and just getting past that.*

Participant 36: *Just all the good information and just knowledgeable information that I didn't know that I know now and have in my notes and emails that I could look back on and reflect on. Just know that I know it now and I didn't know it before.*

b. Learning from and interacting with peers

Another highly endorsed theme included perceptions regarding the opportunity to interact with and learning from other people who also experienced neuropathic pain after their injury. Participants found it particularly useful to hear from and talk to others regarding how they managed their pain and how their experiences were similar and different. Acknowledging that not everything works for everyone, the ability to hear other people's trial and error experiences was considered valuable. The mixture of participants based on time post-injury seemed to be helpful as several participants who were earlier after injury or who had been socially isolated commented on the value of learning from people who had lived with pain longer or had been exposed to more people with SCI than them. This interaction appeared to normalize their pain by the realization that they were not alone in having pain after their SCI. It also appeared to inspire hope in being able to live with and manage pain.

Participant 15: *People were willing to open up and talk about things that I don't think that they would be otherwise willing to open up and speak about in a different environment. It was real nice. I'm pretty sure a lot of people found something that they probably hadn't come across because we covered a lot, meditation and..* *medication, meditation, a wide range..* *or not a wide range, but a nice-sized range of different ways to adjust the pain.*

Participant 17: *I liked being able to hear other people, what they are going through and to see how they manage their pain. And just to know that I'm not the only person going through something like this, it's comforting to know.*

Participant 19: *I really liked how it was in a setting with other people who are also suffering from spinal cord injuries and hearing from their experiences. And I'm pretty new, I'm a year out of my injury. And to hear how people who've had this injury for a lot longer, how they've been managing their pain.*

Participant 23: *I would say it helped me understand my pain. We would've a little group session, we would chat after a slide or two and really just get to hear what other people have to say and what they do differently. That if they've tried this particular way or strategy that how it affected them, and seeing if you could apply it to yourself at some point.*

c. Learning about non-pharmacological approaches and ways to self-manage pain

Participants expressed an appreciation for learning about a variety of nonpharmacological options available to them. They mentioned that the pain education sessions made them more aware about factors that may trigger their pain and how to avoid them, as well as different self-management techniques. For some participants many of the approaches mentioned were new and novel (e.g., meditation, creams, TENS), and for others the education was a good reminder of modalities that they knew of but had not employed recently. A few participants expressed that they had misconceptions regarding non-pharmacological approaches (i.e., spinal cord injury patients shouldn't use ice or heat) that were corrected by the educational sessions and thus expanded their self-management strategies.

Participant 11: *The benefits are that now I actually think of the differences in what I'm doing with myself to see what's affecting or how it starts using some littlest things from heat or coldness, if I'm in a position for a long time, or I just pay attention to that more often now. I'll actually think about how do I feel right now? Or is it nervousness or is it just I need to adjust myself or things of that nature.*

Participant 17: *Before, I was thinking that my pain, I just got to deal with it. I realize…you can just use yoga, stretching, and breathing, and different stuff like that. And I saw it before, I never really thought those things work, but he reinforced to tell me that those things do work. I just got to keep trying, which one. I find the one that's right for me, that will help me with the pain. Before, I just thought, “Man, that crap ain't going to work.*"

Participant 19: *I thought the section on alternative medicine was really interesting. So acupuncture and yoga, deep breathing. Because like I said, I'm not one for pharmaceuticals, so I'm just trying to find other ways to lessen the pain. I mean, just hearing about how there are a lot of other alternatives to pharmaceuticals, and some of them I had heard of before and others that I'm really excited to do.*"

d. Learning about pathophysiology of pain

This theme included the participants' perceptions regarding the more theoretical components of the pain education concerning pain classification, and pathophysiological mechanisms of pain. Although some mentioned that they did not remember all the terms, they appreciated being able to better understand the various types of pain that occur after injury and the underlying mechanisms of pain. Some participants also mentioned that the educational information encouraged them to search for more information.

Participant 10: *I think it was informative to learn about pain, the science behind it and learning where the pain comes from, and things like that. I thought that was interesting.*

Participant 14: *Then, I started learning about the neuro pathways. I like learning stuff. Once I went home, I Googled about the neuro pathways and how the medicine affects, what's targeting where, and all that stuff.*

Participant 17: *There's things that I learned that I didn't learn throughout my time in the hospital and throughout my injuries. So, it was good to get just more a little in depth with the pain management and why I have pain, and just to get a whole background of what I'm experiencing and why.*

Participant 25: *The education was good because again, it spoke about..* *it also taught us about some of the underlying reasons for our pain, knowing the pathology, the background, why our pain is maybe happening.*

e. Learning about pain medication

Many participants found it helpful to better understand the rationale for their medication prescriptions and how the medications may work. Additionally, it was enlightening for some to learn that the medications may not have the same effects in all people. Overall, participants expressed that education on medications is empowering because it can confirm that they are doing the right things or provide them with knowledge or questions to discuss with their physicians (e.g., trying a different drug or changing a drug dosage).

Participant 2: *Yeah, it informed me a lot on the medicine. I actually even adjusted my back with one dose, just looking at what we looked at and what we spoke about, so it definitely informed me on the medicines. And even when we spo*k*e about Tramadol, I used to think that was probably a long-term cure, but it's not. No, it informed me a lot. Definitely the medicine part was very informative because I don't have anyone to tell me these things.*

Participant 12: *Just to be able to talk to people and hear what everybody else has to say. And the lecture itself was good…. all the slides … had all the meds spread out for us and the doses. I had no clue.*

Participant 17: *So I found that very interesting that I'm clearly doing something right. And I did hear about a couple different pain medications because I'm on Baclofen for my spasms. And there was a couple other ones that they had mentioned that I wanted to present to my doctor to see if that might be an option for me.*

Participant 24: *The part I found helpful was seeing why doctors have prescribed me things and the times they did prescribe me things; I had some medicine like Lyrica. It failed and then I had to switch. So that I found helpful. If I was in my first year of the injury, that would've actually saved me a lot of time. I feel like it was the first time I got a comprehensive picture of what the norm is for options for medicine.*

f. Improving communication about the lived experience with pain

Participants mentioned that they would use the knowledge that they gained in the educational sessions when communicating both with their healthcare providers but also other people, including family and friends. Particularly, their increased understanding of pain, treatments, and terminology appeared to be helpful in communication with others. Additionally, having written information to share with others (family, friends, healthcare providers with limited knowledge of SCI pain) was described as valuable. Some participants said that being able to better verbalize their experiences could help them share the emotional impact of pain, which is often not discussed.

Participant 1: *Now that I'm going back in memory, there was something very useful, which was a pamphlet that you did that talks about the pain and what we go through every day. I wanted to give that to my family who speaks Spanish because my caregiver only speaks Spanish. I would like for her to have it, even though she knows what I go through, but nobody really knows about the emotional part of it because I don't broadcast it all 24 h. I keep a lot of that inside.*

Participant 23: *They were introducing the terminology, basically the technical terms that makes what I'm feeling or experiencing explainable, especially to medical professionals.*

Participant 24: *The surgeons might know, neurologists might know, physical therapy might know, but they're not relaying that information to me, so if I don't know it's rare, then I don't know what to look for..* *so all those things work together to put my knowledge in a better place for doctor's appointments. I feel I have a better strategy I want to work on for the daily, weekly physical and just peace of mind on some of my symptoms that I thought were so rare. They're not, so I'm not worried about them. That's huge.*

Participant 32: *Some of the words, I start to learn the new words. I ask them what that means, “Okay. That's what is hurting. This is what it feels.” It's pretty good. It's been good educational for me. When I tell other people, I'm like, “Hey, this is what's going on with me and this is what's helping out for me. I don't know if it help out for you, but it's helping me out.”*

#### 3.1.2 Content and delivery of pain education

a. Positive

Participants provided their perspectives regarding what they found to be positive regarding the content and delivery of the pain education. The amount of information was mentioned to be sufficient although some thought the pain education program was too short. The group format including being able to interact with the others in the group while seeing slides and discuss the content was perceived to be very helpful. To be able to have difficult concepts explained in person when they were presented was also mentioned as being positive.

Participant 2: *No, actually it was a really good amount. I actually thought it was shorter than I expected, which I guess is a good thing for people don't feel good sometimes. You don't have a lot of time. But I think it was a great amount of time because it really wasn't too long and boring. It was good. I liked it.*

Participant 15: *Mostly, I liked the environment. It was a cool environment. …It wasn't boring. It wasn't … a somber environment. Things were upbeat. …It was interactive. A lot of people were not scared to share what they were going through or give tips and give feedback on what was going on. It was a cool environment to be in, discussing something that's not really cool at all.*

Participant 18: *So to me, visualizing also the information by saying it and visualizing it, really trying to maintain that information in my brain. So it really did help a lot.*

*Also the combination of the me and the other people who were involved. They were involved also in the talking, just also to provide some personal expertise regarding the area that x was explaining. … The connection and the communication, that was very important.*

b. No benefit or negative

Participants provided their perspectives regarding those things that they found to be of no benefit or perceived as negative. One negative perception was that some felt that the focusing on pain made pain worse or even triggered their pain. Additionally, some felt that despite information about how to manage pain was interesting it did not apply to them or change the fact that pain was refractory and was not going to resolve. Some expressed that they did not benefit from the educational sessions as they had been injured for a long time and heard it all before.

Participant: 8: *I have so many years involved in the pain field as it relates to my body, so pretty much all of it, I've heard of it. But I'm going on 40 years now, injury.*

Participant 35: *… just because you're learning these things and you're finding out about these things, doesn't actually mean anything can be resolved when it comes to the pain, unfortunately. Learning about different pharmaceuticals doesn't actually do anything. Even taking some of the pharmaceuticals sometimes doesn't actually do anything. So just because you're becoming more educated on the subject, doesn't mean you can actually do anything about the subject.*

Participant37: *The constant reminder of pain. I don't think about it and it doesn't hurt at the moment I speak about it or think about it, it's like, “Ah, I feel the pain now.”*

c. Change suggestions

Participants suggestions were constructive where some expressed that they preferred pain education in person rather than on Zoom. One person suggested to making the program available to family and friends to help them understand what they were going through. Other suggestions included making written material available to all participants and to include specific instructions about some of the self-management approaches rather than just learning that other people have used it.

Participant 1: *Thinking out loud, that'd be a good one-day program, also, that you have. Invite the family members and friends to a one-day seminar, a couple hours and go through everything that we go through, that we don't talk about…..*

Participant 10: *It was a little bit confusing when there was just people's quotes. I guess that it was nice to have people's quotes there, but also sometimes it was just quotes and not actual information about the subject. It was just people's perspectives. That was a bit confusing.*

Participant 17: *I mean, maybe it would've been better to be in person, but I understand that having a bunch of people available at the same time is difficult, but things are always better when you're face-to-face, and it's just different being on the phone.*

Participant 29: *If I had something to read on, I probably could better understand…*

### 3.2 Non-parametric analysis

#### 3.2.1 Baseline vs. post pain education changes

Given that assumptions of normality were violated across most quantitative measures, nonparametric Wilcoxon Signed Rank tests were used to compare baseline values to post pain education values (Table 3). These results showed that both pain interference with activities and difficulty dealing with pain were significantly but modestly lower after the educational sessions. In contrast, no statistically significant changes were observed with respect to pain interference with mood or pain interference with sleep. Similarly, none of the MPI-SCI subscales or worst pain intensity changed significantly after pain education.

**Table 3:** Baseline vs. post pain education changes.

Measure	Baseline		Post education		Wilcoxon sign rank test	
ISCIBPDS (0–10)	median	IQR	median	IQR	Z	*p*-value
Worst pain intensity	8	3.75	7	3.75	0.081	0.418
Pain interference with activities	6	6.5		7.5	2.261	0.024*
Pain interference with mood	5.5	8.0	4	6.0	1.583	0.113
Pain interference with sleep	6	8.75	5	6.0	0.738	0.461
Difficulty dealing with pain (0–10)	6	5.0	5	5.0	2.177	0.029*
MPI-SCI (0–6)						
Pain Severity	4	2.75	4.5	3.0	−0.94,	0.925
Life Interference	3	3.84	3.25	3.09	−1.244	0.179
Life Control	4	2.33	3.83	4.09	0.938	0.348
Affective Distress	2.33	3.25	2.5	2.59	−1.531	0.126
Support	5	2.59	4.67	2.50	1.491	0.136

ISCIPBDS, international SCI pain basic dataset; MPI-SCI, multidimensional pain inventory SCI-version; IQR, interquartile range.

* *p* < 0.05.

## 4 Discussion

The current study was primarily designed as a qualitative study within a mixed method framework to capture the effects of a brief 4-week pain education program conducted in small virtual group sessions among those living with SCI who experienced moderate to severe neuropathic pain. We primarily sought to examine participants' perspectives regarding the pain education program, including the content covered and their overall thoughts and feelings about the delivery of the information presented. Our analyses showed that participants perceived the pain education to be positive, especially with regards to having a better understanding of their own pain and the underlying mechanisms, learning about treatment options including medication and self-management, how their peers managed their pain; and using this knowledge to improve their ability to talk to others about their pain. Participants also mentioned the topics discussed, and the small group interactive settings as positive aspect of the education, although some did not benefit or felt that focusing on pain made pain more obvious to them. Suggestions provided by the participants included in person sessions rather than virtual and making the material available to family and friends to help them understand what they were going through. Other suggestions included making written material available to all participants and include specific instructions about some of the self-management approaches rather than just learning that other people have used it. The overall benefit was consistent with small but significant improvements in perceived pain interference with daily activities and difficulty in dealing with pain.

The opportunity to interact with and learn from other people who also experienced neuropathic pain after their SCI was frequently mentioned as a valued benefit of the pain education sessions. Participants found it particularly helpful to hear from and talk to others with issues like their own regarding how they managed their pain and what their experiences were with different treatment approaches. This interaction appeared to normalize their pain and emphasize that they were not alone in dealing with persistent and often severe pain after their SCI. Similar results have been reported elsewhere (29, 30) showing that peer interaction after SCI has positive effects on self-efficacy and may reduce the number of readmissions (31). The interaction with peers who have experience with various pain management approaches may introduce more treatment options, provide hope, and increase engagement (32, 33).

Participants reported that the information presented enhanced their knowledge of SCI and provided them with a better basis for communicating not only with their healthcare providers but also with family and friends. Prior work by our research group has identified healthcare provider communication challenge as a significant barrier to effective pain management for those with SCI (34). Consequently, improvements in patients' ability to communicate their needs to their care team is a potentially beneficial aspect of the *SeePain* program. Participants also highlighted that learning about medications and non-pharmacological approaches to pain management were particularly beneficial, as many reported having little to no information regarding the different classes of pain medications commonly prescribed. Similar treatment related barriers or a general lack of pharmacological understanding have been reported in other studies (34, 35).

Interestingly yet unsurprisingly, our results may suggest that those who were less informed due to shorter time since injury or had limited access to information would benefit more, not only from the pain education itself, but also from the interaction with individuals who had more experience with managing pain. It is likely that these individuals, having been exposed to various treatment options over time, possessed a more nuanced understanding of the information presented, and thus experienced a ceiling effect for any benefits that may have been otherwise reported by those with less time living with SCI.

The reduction in pain interference with activity and difficulty in dealing with pain observed after the pain education relative to baseline supports the qualitative data and aligns with findings from previous studies on pain education. For instance, a randomized controlled trial on chronic low back pain including either 6 weeks of standard physiotherapy or standard care with the addition of pain education program demonstrated that the addition of pain education significantly reduced disability and pain intensity while improving overall well-being (36). Moreover, a systematic review and meta-analysis on pain neuroscience education for fibromyalgia patients found that it reduced pain intensity short-term with a moderate clinical effect without any effects on anxiety and catastrophizing (37). These authors suggested that future research studies should determine the most useful ways to deliver pain neuroscience education to patients and potential combinations with other treatments. A large internet survey involving 465 people with chronic pain found that participants who felt that pain education had changed their views on pain and changed their way of self-managing pain, were more likely to experience lower pain intensity scores and higher expectations of recovery (38). Like the study involving people with fibromyalgia (37), our study failed to detect statistically significant improvements in affective distress or pain interference with mood. To contextualize the non-significant result, the small effect size for pain interference with mood would have required a sample size of 165 people to detect a statistically significant difference. We also did not detect any changes in pain intensity or severity, life interference, life control, or social support. The observed improvements in pain interference with activities and difficulty in dealing with pain do support indirect positive effects across psychological factors commonly perceived to be negatively affected in those living with SCI who experience neuropathic pain. However, these improvements were small and the study observations by Sidiq and colleagues (36) suggest that combining pain education with other interventions may result in better improvements in these outcomes.

Several meta-analyses reveal that the implementation of pain education either combined as part of a multidimensional treatment approach or in isolation, can improve psychosocial measures of pain and physical function across a wide range of chronic pain conditions (39–45). For example, in a recent study by Marris et al (39) pooled data from 1,024 participants with chronic musculoskeletal pain and found that the inclusion of a pain education component to standardized physical therapy improved clinical measures of both short-term and long-term pain severity and disability. In a similar population and using a mixed method approach, Watson et al (40) consolidated data from 755 participants and discovered that the introduction of a pain science education program, specifically designed to help individuals reconceptualize their pain as less threatening, helped to mitigate subjective pain ratings, disability, pain catastrophizing, and kinesiophobia in the short or medium term. Subsequently, researchers have expanded such examinations beyond those with musculoskeletal pain and offered evidence for the benefits of similar pain education programs across those living with chronic migraines (42), cancer pain (43, 44), and pain related to limb amputation (45).

Ultimately our findings align with those of previous research groups (13, 46, 47) and suggest that the implementation of a brief pain education program may help to equip individuals living with SCI and comorbid neuropathic pain with information that enables them to significantly reduce perceived pain interference with activities, while simultaneously enhancing their ability to cope with their pain symptoms. Notably, Burns and colleagues (46) showed that participation in a 10-week multidimensional pain management program that incorporated a heavy education component led to significant reductions in life interference at the intervention exit and improved subjective ratings of life control at the 12-month follow-up. The authors also noted improvements in the maintenance of coping strategies over the intervention period. Norrbrink Budh et al (13) similarly showed that when compared to controls, those who participated in a 10-week pain management program which included educational sessions, behavioral therapy, relaxation, stretching, light exercise, and body awareness training, afforded significant improvements across indices of depression and sense of coherence. Given that sense of coherence is a construct related to how an individual successfully copes with stressors, individual improvements in the ability to deal with pain in the current study make outcome comparisons justifiable. Heutink et al. (47) also compared the effects of a 10-week multidisciplinary treatment program consisting of educational, cognitive, and behavioral components to a waitlist control. The authors found significant reductions in pain intensity, pain-related disability, anxiety, and increases in activity participation in the intervention group alone. Measures at the 3-month follow-up also indicated that indices of anxiety and activity participation remained significantly improved, the results of which were not observed among those allocated to the control group. The authors suggested that the amelioration of these symptoms could be due to the intervention helping individuals to positively reframe their current situation while bolstering their ability to restructure potentially dysfunctional cognitions associated with how they experience their pain. Additionally, the intervention included a strong emphasis on activity participation, therefore, the authors proposed that such improvements could be the result of this focused framework. Finally, the findings by Perry et al (48) who found that participation in a multidisciplinary cognitive-behavioral pain management program which consisted of 10 group-based sessions, led to significant and clinically meaningful improvements in self-reported pain symptoms, physical function, and quality of life over those receiving usual care (48) further emphasizes that the optimal utility of pain education is likely as part of a multimodal approach to pain management. Moreover, those in the pain management program demonstrated significant and positive improvements in pain interference over those allocated to usual care, suggesting that such programs could favorably influence indices of mood and functionality.

### 4.1 Limitations

While the current mixed method study indicates that there is clinical utility in the implementation of a brief 4-week pain education program among those with SCI and neuropathic pain, there are several noteworthy limitations that must be addressed when attempting to generalize the results. The primary objective was powered for qualitative analysis, and not for detecting significant changes in quantitative pain variables. Further our group of 36 participants is rather small and may not represent all people with SCI who experience neuropathic pain. For example, we did not include those with mild pain and inadvertently excluded those with lack of internet access or ability to use internet resources. It is also possible that those who were less informed and/or injured for a shorter time benefitted the most from the pain education because they had not yet been introduced to as many different approaches to manage pain compared to those who had been injured a longer time. Because the present study was primarily designed as a qualitative study, there was no control group within the protocol, making group comparisons impossible to ascertain. Finally, the educational program included only 4 h of contact time with the research staff. Previous chronic pain studies suggest far greater contact time (45–100 h) during rehabilitation programs is required to elicit appreciable improvements across biopsychosocial symptoms commonly associated with chronic pain (48, 49). However, the neuropathic pains after SCI are notoriously refractory to treatments which may also have been contributing to the lack of effects on pain intensity and psychosocial impact.

### 4.2 Conclusion

Overall, our findings suggest that the pain education sessions were highly appreciated among those who experienced moderate to severe neuropathic pain following SCI. The perceived benefits included a better understanding about pain, treatment options and self-management in general, learning from and interacting with peers, better understanding about pathophysiology of pain and being able to better communicate with healthcare providers, family, and friends. In contrast, some participants felt that the focus on pain during the educational sessions made pain worse or triggered their pain. Some also thought that pain education did not change the fact that pain was refractory and was not going to resolve.

The usefulness of this brief pain education program is also supported by small but significant reductions in pain interference with activities and difficulty in dealing with pain. These findings suggest that pain education in small peer groups may be useful as part of a multimodal approach to the management of neuropathic pain after SCI. The suggestions provided by participants included a preference for in-person sessions rather than virtual, making pain education available to family and friends, to include written materials consistent with the pain education content, and provide more specific instructions regarding self-management strategies. There was a preference for in-person pain education among some of the participants, which suggests that when possible, this should be an option. However, this is only an option for those with adequate transportation and proximity to research or clinical centers. Therefore, virtual pain education can significantly facilitate access for more people with SCI. These suggestions and the heterogeneity in pain characteristics and psychosocial impact emphasize the need for further development of pain education strategies tailored to individual pain presentations and preferences including previous experience with pain management approaches.

## Data Availability

The raw data supporting the conclusions of this article will be made available by the authors, without undue reservation.

## References

[1] Finnerup NB Norrbrink C Trok K Piehl F Johannesen IL Sorensen JC Phenotypes and predictors of pain following traumatic spinal cord injury: a prospective study. J Pain. (2014) 15(1):40–8. 10.1016/j.jpain.2013.09.008 24268112

[2] Jensen M Hoffman A Cardenas D. Chronic pain in individuals with spinal cord injury: a survey and longitudinal study. Spinal Cord. (2005) 43(12):704–12. 10.1038/sj.sc.3101777 15968299

[3] Siddall PJ McClelland JM Rutkowski SB Cousins MJ. A longitudinal study of the prevalence and characteristics of pain in the first 5 years following spinal cord injury. Pain. (2003) 103(3):249–57. 10.1016/S0304-3959(02)00452-9 12791431

[4] Widerström-Noga EG Felipe-Cuervo E Yezierski RP. Chronic pain after spinal injury: interference with sleep and daily activities. Arch Phys Med Rehabil. (2001) 82(11):1571–7. 10.1053/apmr.2001.26068 11689978

[5] Putzke JD Richards JS Hicken BL DeVivo MJ. Interference due to pain following spinal cord injury: important predictors and impact on quality of life. Pain. (2002) 100(3):231–42. 10.1016/S0304-3959(02)00069-6 12467994

[6] Buscemi V Cassidy E Kilbride C Reynolds FA. A qualitative exploration of living with chronic neuropathic pain after spinal cord injury: an Italian perspective. Disabil Rehabil. (2018) 40(5):577–86. 10.1080/09638288.2016.1271023 28054832

[7] Widerström-Noga E Anderson KD Perez S Hunter JP Martinez-Arizala A Adcock JP Living with chronic pain after spinal cord injury: a mixed-methods study. Arch Phys Med Rehabil. (2017) 98(5):856–65. 10.1016/j.apmr.2016.10.018 27894730

[8] Norman C Bender JL Macdonald J Dunn M Dunne S Siu B Questions that individuals with spinal cord injury have regarding their chronic pain: a qualitative study. Disabil Rehabil. (2010) 32(2):114–24. 10.3109/09638280903033248 19817663

[9] Norrbrink C Löfgren M. Needs and requests–patients and physicians voices about improving the management of spinal cord injury neuropathic pain. Disabil Rehabil. (2016) 38(2):151–8. 10.3109/09638288.2015.1035456 25918963

[10] Hearn JH Cotter I Fine P A Finlay K. Living with chronic neuropathic pain after spinal cord injury: an interpretative phenomenological analysis of community experience. Disabil Rehabil. (2015) 37(23):2203–11. 10.3109/09638288.2014.1002579 25601144

[11] Cruz-Almeida Y Martinez-Arizala A Widerström-Noga EG. Chronicity of pain associated with spinal cord injury: a longitudinal analysis. J Rehabil Res Dev. (2005) 42(5):585. 10.1682/JRRD.2005.02.0045 16586184

[12] Widerström-Noga E Anderson KD Perez S Martinez-Arizala A Cambridge JM. Subgroup perspectives on chronic pain and its management after spinal cord injury. J Pain. (2018) 19(12):1480–90. 10.1016/j.jpain.2018.07.003 30056113

[13] Budh CN Kowalski J Lundeberg T. A comprehensive pain management programme comprising educational, cognitive and behavioural interventions for neuropathic pain following spinal cord injury. J Rehabil Med. (2006) 38(3):172–80. 10.1080/16501970500476258 16702084

[14] Brach C Harris LM. Healthy people 2030 health literacy definition tells organizations: make information and services easy to find, understand, and use. J Gen Intern Med. (2021) 36(4):1084–5. 10.1007/s11606-020-06384-y 33483812 PMC8042077

[15] Widerström-Noga E Anderson KD Robayo LE Perez S Martinez-Arizala A Calle-Coule L Development of a pain education resource for people with spinal cord injury. Front Public Health. (2023) 11:1197944. 10.3389/fpubh.2023.1197944 37554730 PMC10406314

[16] Steer RA Brown GK Beck AT Sanderson WC. Mean beck depression inventory–II scores by severity of major depressive episode. Psychol Rep. (2001) 88(3_suppl):1075–6. 10.2466/pr0.2001.88.3c.1075 11597055

[17] Dozois DJ Dobson KS Ahnberg JL. A psychometric evaluation of the beck depression inventory–II. Psychol Assess. (1998) 10(2):83. 10.1037/1040-3590.10.2.83

[18] Harris CA Joyce L. Psychometric properties of the beck depression inventory-(BDI-II) in individuals with chronic pain. Pain. (2008) 137(3):609–22. 10.1016/j.pain.2007.10.022 18079063

[19] Babor TF Higgins-Biddle JC Saunders JB Monteiro MG, Organization WH. AUDIT: the alcohol use disorders identification test: guidelines for use in primary health care. 2001.

[20] Allen JP Litten RZ Fertig JB Babor T. A review of research on the alcohol use disorders identification test (AUDIT). Alcohol Clin Exp Res. (1997) 21(4):613–9. 10.1111/j.1530-0277.1997.tb03811.x 9194913

[21] Skinner HA. The drug abuse screening test. Addict Behav. (1982) 7(4):363–71. 10.1016/0306-4603(82)90005-3 7183189

[22] Yudko E Lozhkina O Fouts A. A comprehensive review of the psychometric properties of the drug abuse screening test. J Subst Abuse Treat. (2007) 32(2):189–98. 10.1016/j.jsat.2006.08.002 17306727

[23] Widerström-Noga E Biering-Sørensen F Bryce T Cardenas DD Finnerup NB Jensen MP The international spinal cord injury pain basic data set (version 2.0). Spinal Cord. (2014) 52(4):282–6. 10.1038/sc.2014.4 24469147

[24] Widerström-Noga EG Cuervo F-Broton E Duncan JG Yezierski RC P R. Perceived difficulty in dealing with consequences of spinal cord injury. Arch Phys Med Rehabil. (1999) 80(5):580–6. 10.1016/S0003-9993(99)90203-4 10326925

[25] Barrett H McClelland JM Rutkowski SB Siddall PJ. Pain characteristics in patients admitted to hospital with complications after spinal cord injury. Arch Phys Med Rehabil. (2003) 84(6):789–95. 10.1016/S0003-9993(02)04944-4 12808528

[26] Kerns RD Turk DC Rudy TE. The west haven-Yale multidimensional pain inventory (WHYMPI). Pain. (1985) 23(4):345–56. 10.1016/0304-3959(85)90004-1 4088697

[27] Widerström-Noga EG Cruz-Almeida Y Martinez-Arizala A Turk DC. Internal consistency, stability, and validity of the spinal cord injury version of the multidimensional pain inventory. Arch Phys Med Rehabil. (2006) 87(4):516–23. 10.1016/j.apmr.2005.12.036 16571391

[28] Charmaz K. Grounded theory. Qualitative psychology: a practical guide to research methods. (2015) 3:53–84.

[29] Chaffey L Bigby C. Health education by peers with spinal cord injury: a scoping review. J Dev Phys Disabil. (2018) 30:141–54. 10.1007/s10882-017-9569-6

[30] Fernandez GE Anderson KD Vastano R Frank SI Robayo LE Cherup NP Perspectives of people with spinal cord injury on a pain education resource. Front Public Health. (2024) 12:1385831. 10.3389/fpubh.2024.1385831 38962773 PMC11220275

[31] Jones ML Gassaway J Sweatman WM. Peer mentoring reduces unplanned readmissions and improves self-efficacy following inpatient rehabilitation for individuals with spinal cord injury. J Spinal Cord Med. (2021) 44(3):383–91. 10.1080/10790268.2019.1645407 31403374 PMC8081317

[32] Barclay L Hilton G Fossey E Ponsford J Downing M Analytis P Peer mentor contributions to an early intervention vocational rehabilitation specialist service following trauma: a qualitative study. Disabil Health J. (2025) 18(1):101680. 10.1016/j.dhjo.2024.101680 39152070

[33] Gassaway J Jones ML Sweatman WM Young T. Peer-led, transformative learning approaches increase classroom engagement in care self-management classes during inpatient rehabilitation of individuals with spinal cord injury. J Spinal Cord Med. (2019) 42(3):338–46. 10.1080/10790268.2017.1385992 29037112 PMC6522966

[34] Widerstrom-Noga E Anderson KD Perez S Martinez-Arizala A Calle-Coule L Fleming L. Barriers and facilitators to optimal neuropathic pain management: sCI consumer, significant other, and health care provider perspectives. Pain Med. (2020) 21(11):2913–24. 10.1093/pm/pnaa058 32219441

[35] Löfgren M Norrbrink C. But I know what works”–patients’ experience of spinal cord injury neuropathic pain management. Disabil Rehabil. (2012) 34(25):2139–47. 10.3109/09638288.2012.676146 22512334

[36] Sidiq M Muzaffar T Janakiraman B Masoodi S Vasanthi RK Ramachandran A Effects of pain education on disability, pain, quality of life, and self-efficacy in chronic low back pain: a randomized controlled trial. PLoS One. (2024) 19(5):e0294302. 10.1371/journal.pone.0294302 38805446 PMC11132453

[37] Suso-Martí L Cuenca-Martínez F Alba-Quesada P Muñoz-Alarcos V Herranz-Gómez A Varangot-Reille C Effectiveness of pain neuroscience education in patients with fibromyalgia: a systematic review and meta-analysis. Pain Med. (2022) 23(11):1837–50. 10.1093/pm/pnac077 35587171

[38] Mittinty MM Vanlint S Stocks N Mittinty MN Moseley GL. Exploring effect of pain education on chronic pain patients’ expectation of recovery and pain intensity. Scand J Pain. (2018) 18(2):211–9. 10.1515/sjpain-2018-0023 29794302

[39] Marris D Theophanous K Cabezon P Dunlap Z Donaldson M. The impact of combining pain education strategies with physical therapy interventions for patients with chronic pain: a systematic review and meta-analysis of randomized controlled trials. Physiother Theory Pract. (2021) 37(4):461–72. 10.1080/09593985.2019.1633714 31250682

[40] Watson JA Ryan CG Cooper L Ellington D Whittle R Lavender M Pain neuroscience education for adults with chronic musculoskeletal pain: a mixed-methods systematic review and meta-analysis. J Pain. (2019) 20(10):1140.e1–1140.e22. 10.1016/j.jpain.2019.02.011 30831273

[41] Tegner H Frederiksen P Esbensen BA Juhl C. Neurophysiological pain education for patients with chronic low back pain: a systematic review and meta-analysis. Clin J Pain. (2018) 34(8):778–86. 10.1097/AJP.0000000000000594 29443723

[42] Minen MT Kaplan K Akter S Espinosa-Polanco M Guiracocha J Khanns D Neuroscience education as therapy for migraine and overlapping pain conditions: a scoping review. Pain Med. (2021) 22(10):2366–83. 10.1093/pm/pnab131 34270769 PMC8677457

[43] Bennett MI Bagnall A-M Closs SJ. How effective are patient-based educational interventions in the management of cancer pain? Systematic review and meta-analysis. Pain. (2009) 143(3):192–9. 10.1016/j.pain.2009.01.016 19285376

[44] Oldenmenger WH Geerling JI Mostovaya I Vissers KCP de Graeff A Reyners AKL A systematic review of the effectiveness of patient-based educational interventions to improve cancer-related pain. Cancer Treat Rev. (2018) 63:96–103. 10.1016/j.ctrv.2017.12.005 29272781

[45] Pantera E Pourtier-Piotte C Bensoussan L Coudeyre E. Patient education after amputation: systematic review and experts’ opinions. Ann Phys Rehabil Med. (2014) 57(3):143–58. 10.1016/j.rehab.2014.02.001 24726790

[46] Burns AS Delparte JJ Ballantyne EC Boschen KA. Evaluation of an interdisciplinary program for chronic pain after spinal cord injury. PM R. (2013) 5(10):832–8. 10.1016/j.pmrj.2013.05.004 23684779

[47] Heutink M Post MW Bongers-Janssen HM Dijkstra CA Snoek GJ Spijkerman DCM The CONECSI trial: results of a randomized controlled trial of a multidisciplinary cognitive behavioral program for coping with chronic neuropathic pain after spinal cord injury. Pain. (2012) 153(1):120–8. 10.1016/j.pain.2011.09.029 22100355

[48] Perry KN Nicholas MK Middleton JW. Comparison of a pain management program with usual care in a pain management center for people with spinal cord injury-related chronic pain. Clin J Pain. (2010) 26(3):206–16. 10.1097/AJP.0b013e3181bff8f3 20173434

[49] Guzmán J Esmail R Karjalainen K Malmivaara A Irvin E Bombardier C. Multidisciplinary rehabilitation for chronic low back pain: systematic review. Br Med J. (2001) 322(7301):1511–6. 10.1136/bmj.322.7301.1511 11420271 PMC33389

