# Supplementary material for: Impact of a pain education program for people with spinal cord injury who experience neuropathic pain
Source: Front Pain Res (Lausanne). 2025 May 27;6:1569446. doi: 10.3389/fpain.2025.1569446 (PMC12148921; doi:10.3389/fpain.2025.1569446)
Supplement: Supplementary file 1 [file Datasheet1.pdf]

Pain after spinal cord injury

PART 1

Background

"Consumer perspectives on a multimodal approach to make neuropathic pain manageable after SCI" funded by Department of Defense.

Up to 80% of people develop some pain within their first year after SCI. People have many important questions about their injury including pain. For example: Why do I have pain? What kinds of pain are common after SCI? What can I do myself to manage my pain? What are the treatment options? What can I expect long-term? What are other people's experiences and thoughts on pain and pain treatments? We will try to answer these and other questions in 4 separate sessions. In each session we will present information and you can ask questions. The purpose of the pain education is to help people better understand their own pain and their treatment options to make their chronic pain more manageable.

Reactions to pain education

"I read the whole thing and most of the stuff, it helps you understand it clearly and it's stuff that I learned on my own. And all the stuff that I learned on my own without nobody telling me, everything is here."

"It was interesting…the other side of the story, to hear the health care providers, the doctors… And it helps … me see how to better try to communicate with them so they can understand. Because for them it's also like they don't understand your pain."

"It almost had me in tears because I had no way to explain this to anyone. And when I explain it to them, I know they don't get it. I mean, they might. They can put it in perspective, but they don't get it. But the quotes that you have down here, they send chills up and down."

Content for Part 1

1. What is pain and what influences the pain experience?
2. The nervous system
3. SCI classification
4. Pain classification
5. Nociceptive pain - What is it? Why does it happen? What does it feel like?
6. Neuropathic pain - What is it? Why does it happen? What does it feel like?

1. What is pain?

“an unpleasant sensory and emotional experience from actual or potential tissue damage.”

Type of pain
What does it feel like?
Is it constant ?

Do you feel depressed, anxious, worried, happy, calm…?

Your Pain Experience is personal

Does your pain hinder your activities, independence, mood, sleep..?

Is your pain manageable? Can it be reduced? Does it take multiple approaches?

“It’s really hard … first to deal with the spinal cord injury but now, in everyday life, you have to deal with the pain…. besides dealing with your everyday of the spinal cord … The pain is.. unbearable sometimes and sometimes you get…immune to the pain...but there are days you can’t deal with the pain and you have to find ways to make it less for you...”

2. The nervous system and pain after SCI

The central nervous system consists of the spinal cord and the brain, and the peripheral nervous system includes all the peripheral nerves. The central nervous system combines all the information from our senses and coordinates all body activities.

In a non-injured person, the nerves communicate with the brain which then interprets the information and tells you exactly from which area of your body are the signals coming from.

After a spinal cord injury (SCI), where the connections between the brain and the body have been damaged, the brain can no longer precisely determine where the pain signals are coming from. Therefore, pain may be experienced in areas of the body with no or only partial sensation.

Several brain areas communicate in a network. Some brain areas are involved in knowing where the pain is, how intense it is, and what it feels like. Other areas are more involved in reactions to pain and some areas can regulate pain.

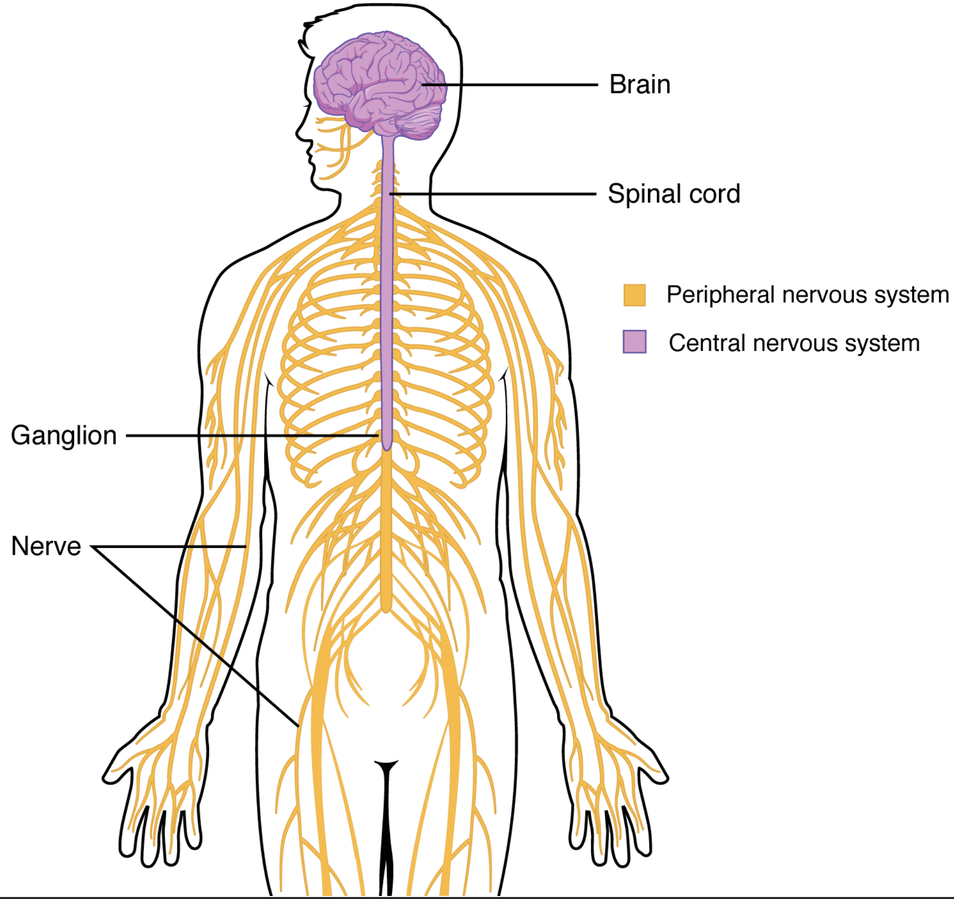

The pains that occur after SCI vary widely in where they are felt, what they feel like, how they respond to treatments, and how they affect your quality of life.

3. Spinal cord injury classification

To better understand pain, it is useful to know some basic information about the spinal cord:

Sensory nerves exit the spinal cord between each vertebra and provide sensation for a specific skin area or "dermatome" of the body.

The “neurological level of SCI” is the section of the spinal cord where the injury occurred. The determination of the level of injury is not only important for determining how severe an injury is but also for determining the pain type.

The ASIA Impairment Scale is the most common method to determine the severity of SCI. When the SCI is classified as complete, there is usually a total loss of sensory and motor function below the level of injury. When the SCI is incomplete, some sensation and/or movement is present below the level of injury.

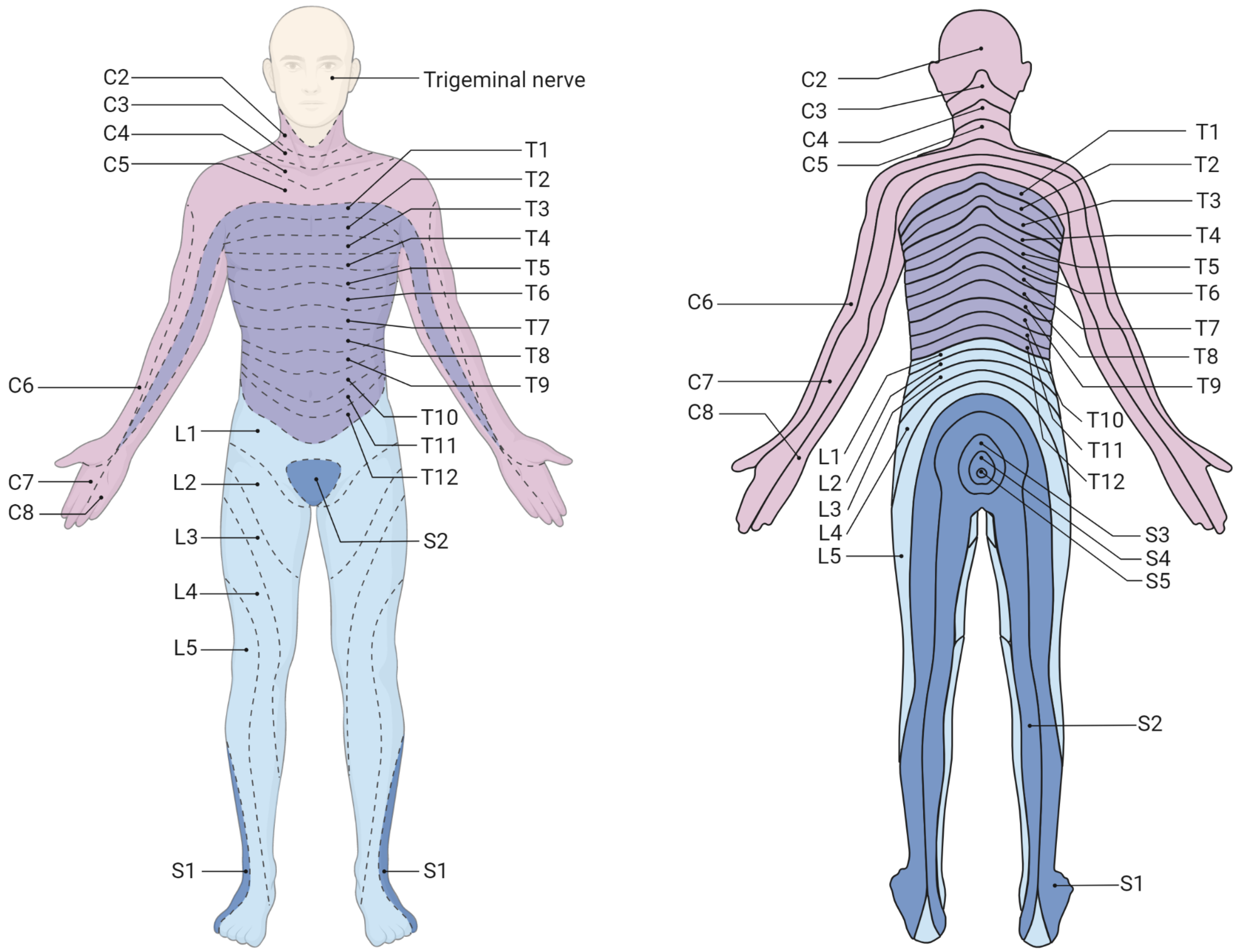

4. Types of pain after SCI

Most people experience more than one type of pain after their SCI. The most common is two or three pains.

There are two major types of pain after SCI:

- **Nociceptive pain** (no-ci-cep-tive), is a pain that a person without an SCI can also experience, such as muscle pain

- **Neuropathic pain** (nyoor-oh-path-ik), which is pain directly caused by the injury to the spinal cord.

We will present the different types of pain in the following slides.

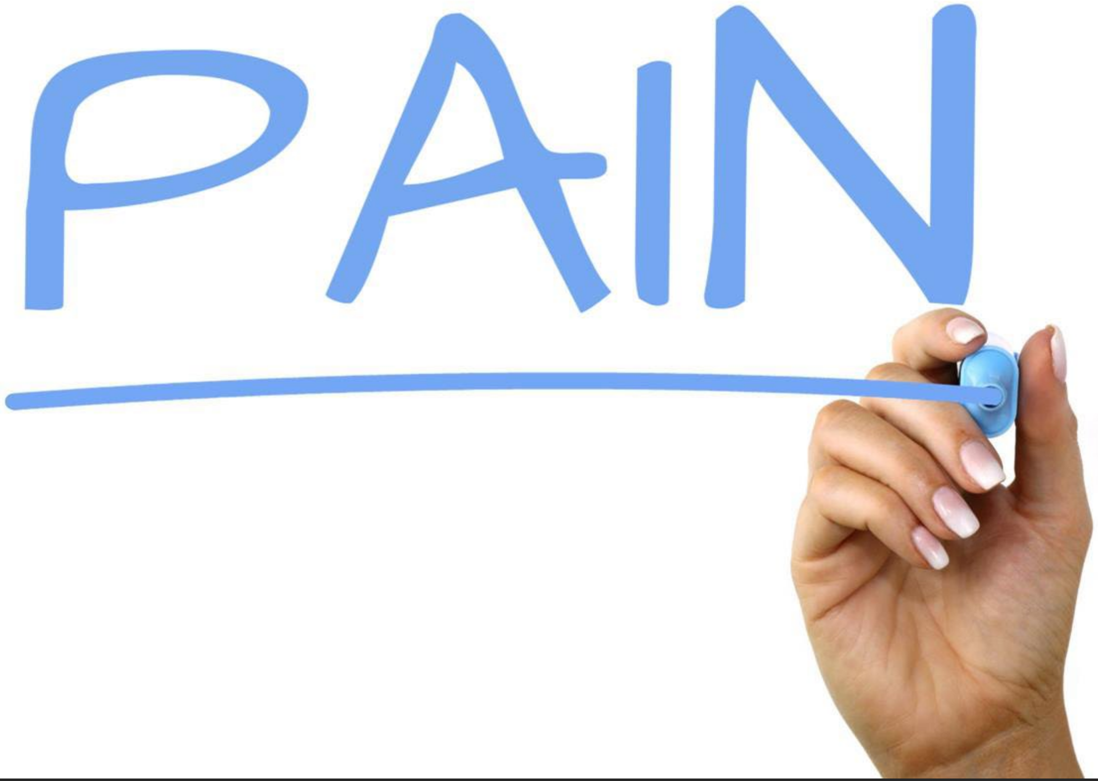

5. Nociceptive Pain – What is it?

Nociceptive pain is generated when nociceptors (pain sensors located throughout the body, including on the skin, in muscle, or internal organs) are activated by specific stimuli like strong mechanical pressure, inflammation, or hot or cold temperature.

For example, heat from a fire can activate the nociceptors in your hand.

Acute

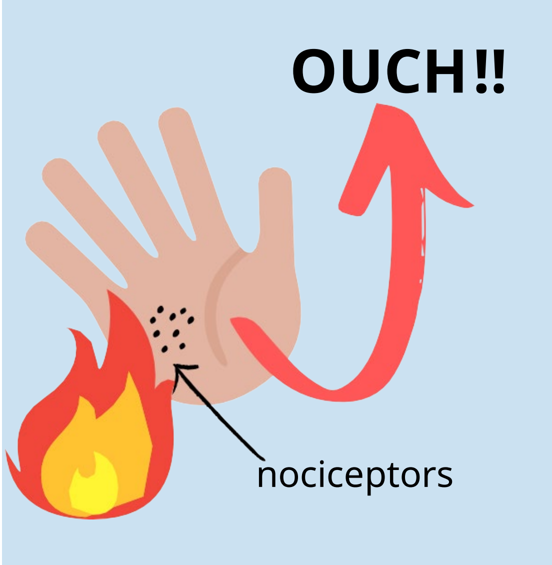

Similarly, the over-extension of a muscle or joint can also activate nociceptors and produce pain.

When nociceptors are activated, they send signals via the spinal cord to the brain and when the signal reaches your brain you feel pain.

Chronic

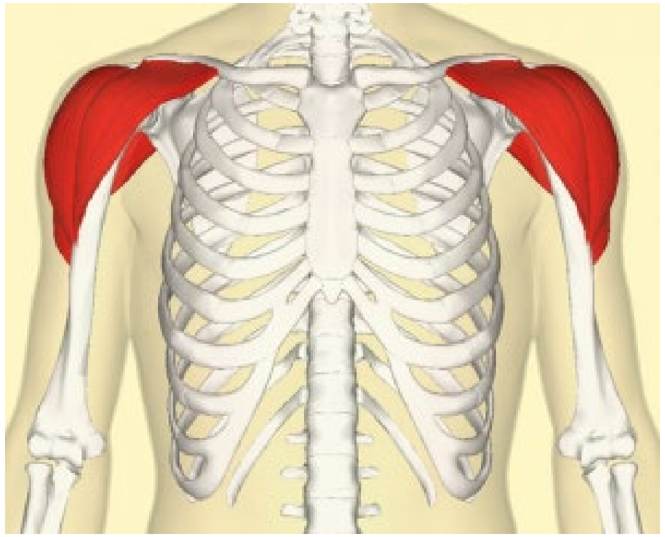

Nociceptive Pain – Why does it happen?

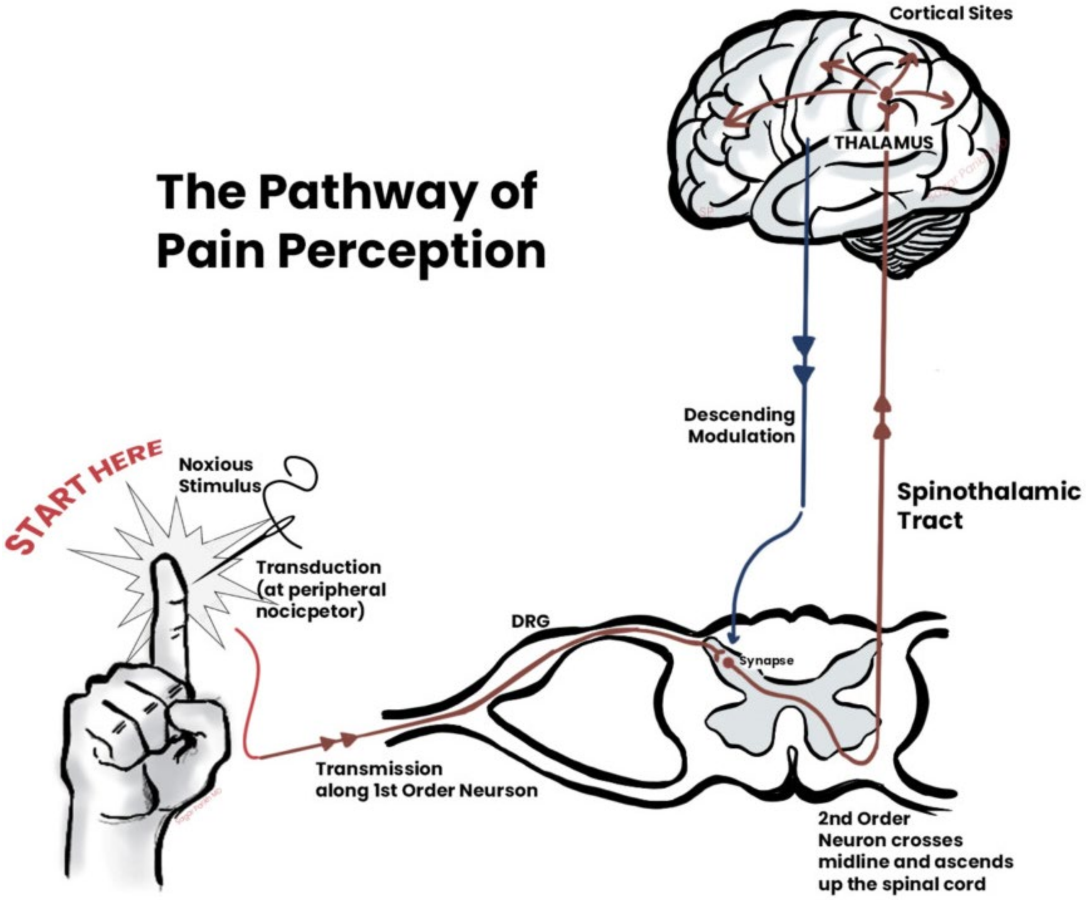

After SCI, a nociceptor can only be activated in an area with full or partial sensation. That means that nociceptive pains are most common above the level of injury but can also happen below the level of injury if the SCI is incomplete.

The most common type of **pain after SCI is nociceptive musculoskeletal pain**. This pain involves nociceptors in muscles, joints, and bones. For example, shoulder injury from daily wheelchair use is a common cause of musculoskeletal nociceptive pain. Musculoskeletal back and neck pain are also common due to sitting for long periods in a wheelchair, or because of muscle spasms.

Another kind of pain is **nociceptive visceral pain** that results from the activation of nociceptors of the thoracic, pelvic, or abdominal organs. It may be caused by constipation or a kidney stone but may not be felt the same way in those who have an SCI compared to uninjured people. It is important to consult with your doctor if you experience new pain in the abdominal area because there could be many reasons for this type of pain.

Nociceptive Pain – What does it feel like?

Musculoskeletal pain may be increased, decreased or changed by movement or a change in position. The painful area may feel tender when touched. This type of pain is often described as ***dull or aching*** by people with SCI.

Visceral pain is diffuse and difficult to localize. After SCI, this pain may be described as ***annoying, cramping, tender, sickening, shooting, tiring, stinging, sharp, pulling***. Constipation, urinary tract infection, stress, full bladder and eating can make pain worse, and emptying of bladder or bowel, or warm weather can make the pain better.

Musculoskeletal pain

“I still can’t sleep on my left side because of the shoulder pain" "twenty-thirty minutes is fine then the shoulder will start throbbing and I... roll around and get the weight off of it.”

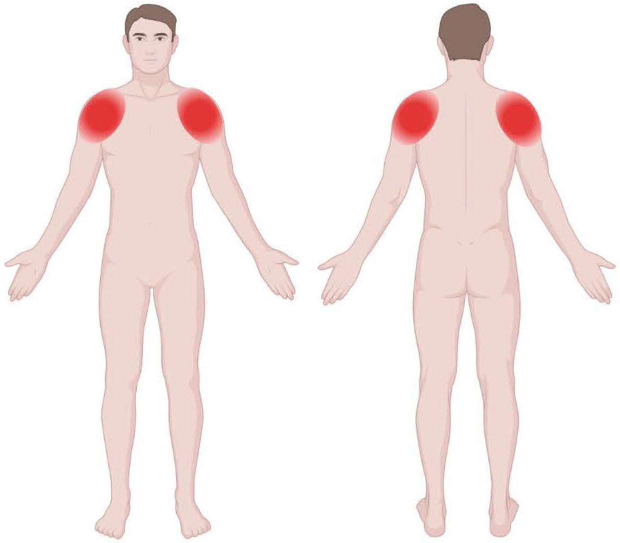

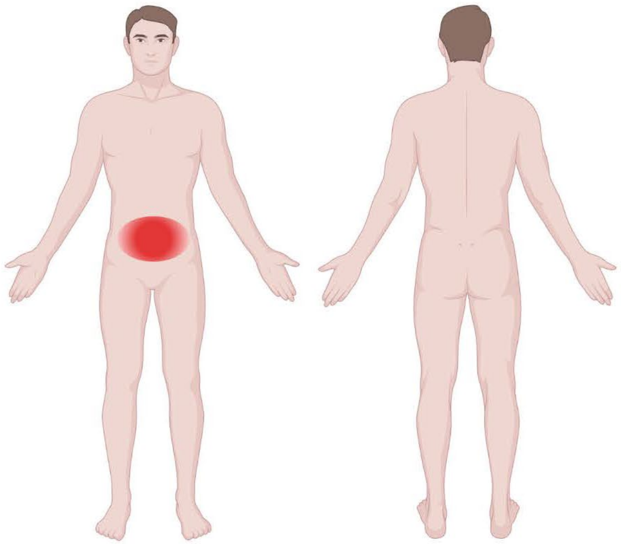

Visceral pain

“When it’s really bad ...my stomach.. hurts a lot and I can’t eat.. maybe I’ll eat less, I will try to rest, Sometimes getting out of the chair just allows me to relax the stomach, the pressure on the area.”

6. Neuropathic pain – What is it?

Neuropathic pain can develop after an injury to the brain, spinal cord, or peripheral nerves. This type of pain is associated with multiple changes in the spinal cord and brain. Neuropathic pain can last long after the initial injury has healed and occurs in about 60% of people who have had an SCI.

Below-level neuropathic pain occurs below the level of spinal cord injury and usually develops over the first year of injury. The figure below shows two different examples for these two different types of pain in an individual with an injury around Thoracic level 5 (black dot).

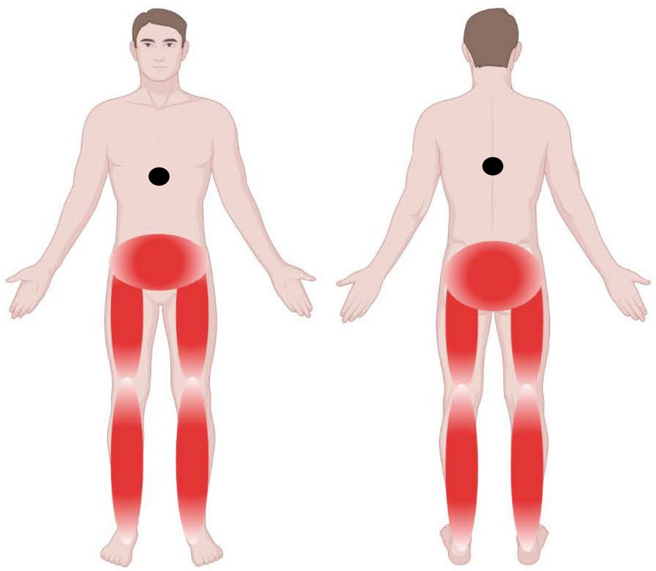

At-level neuropathic pain occurs around the level of SCI and usually starts early after the SCI.

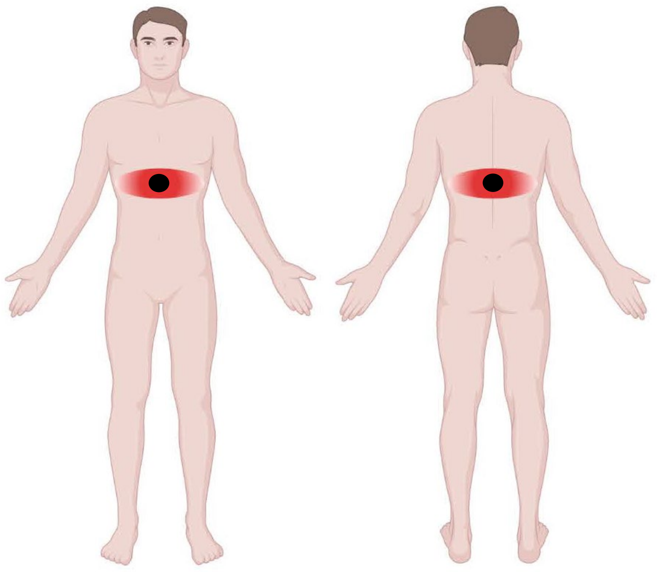

Neuropathic pain – Why does it happen?

There are several mechanisms that contribute to neuropathic pain after SCI. Unfortunately, these are not completely understood. This makes it very difficult to determine the cause of pain and the best treatment. The following slides will show some of the potential reasons for neuropathic pain.

1. Central sensitization

After an injury, the neurons that carry pain signals in the spinal cord and brain can become very sensitive (easily activated). This can lead to **hyperalgesia** which is hypersensitivity to a mild pain stimulus (pinprick) or **allodynia** which is when a stimulus that is not normally painful (light touch or cool temperature) causes pain.

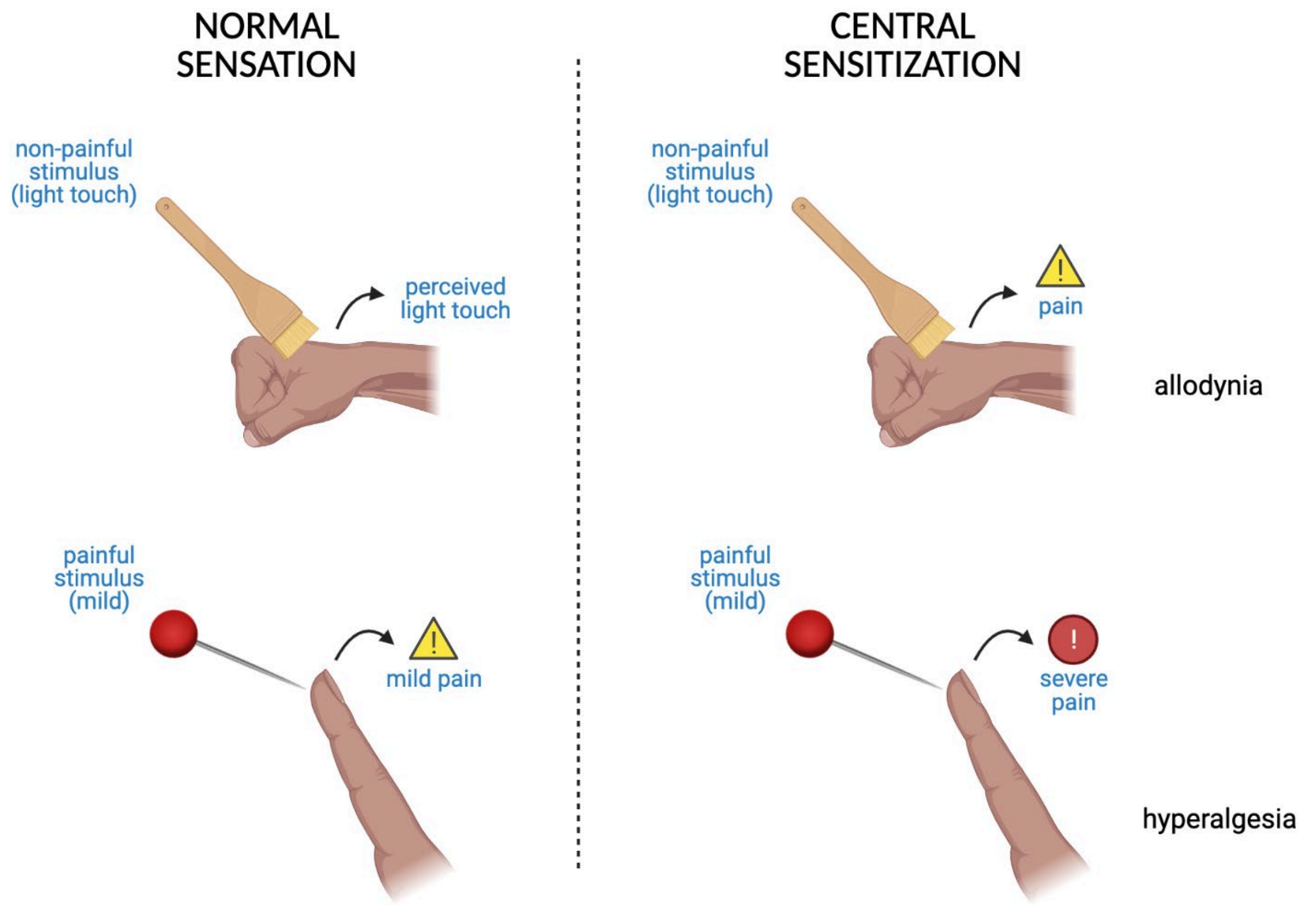

Neuropathic pain – Why does it happen?

2. Reduced inhibition of pain signals

When the spinal cord is injured, systems in the brain and the spinal cord that naturally reduce pain do not work well and as a result, neuropathic pain can develop. You can think of this mechanism as a "brake" and when the brake is not working the pain signals reach the brain at a greater strength than they would do if you did not have an SCI.

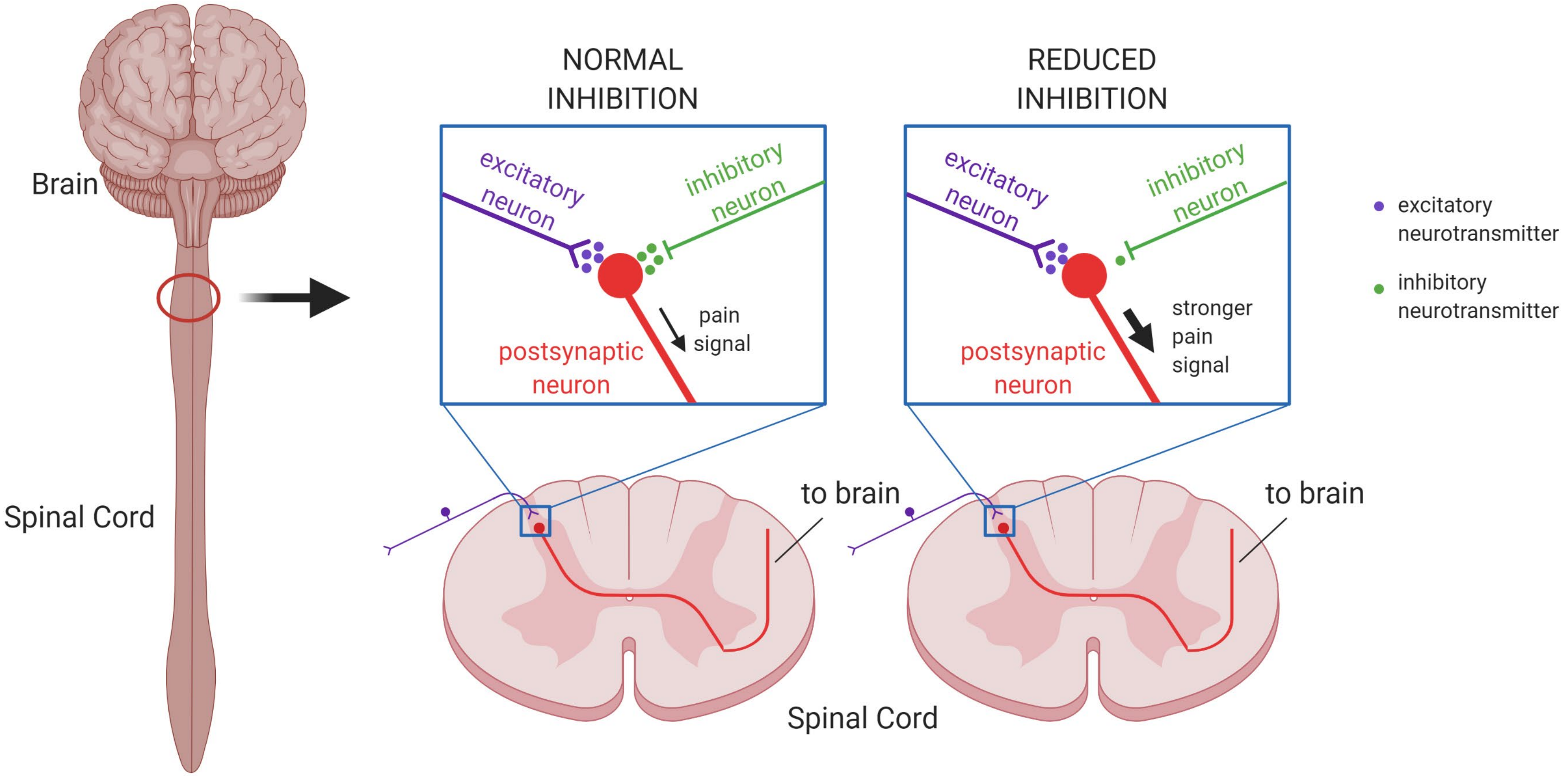

Neuropathic pain – Why does it happen?

3. Microglial activation changes to "support cells"

Cells that surround and support the neurons in the spinal cord and in the brain, called microglia can change after SCI and become "activated". This activation causes inflammatory factors and other substances to be released which causes neurons to become hypersensitive to stimuli.

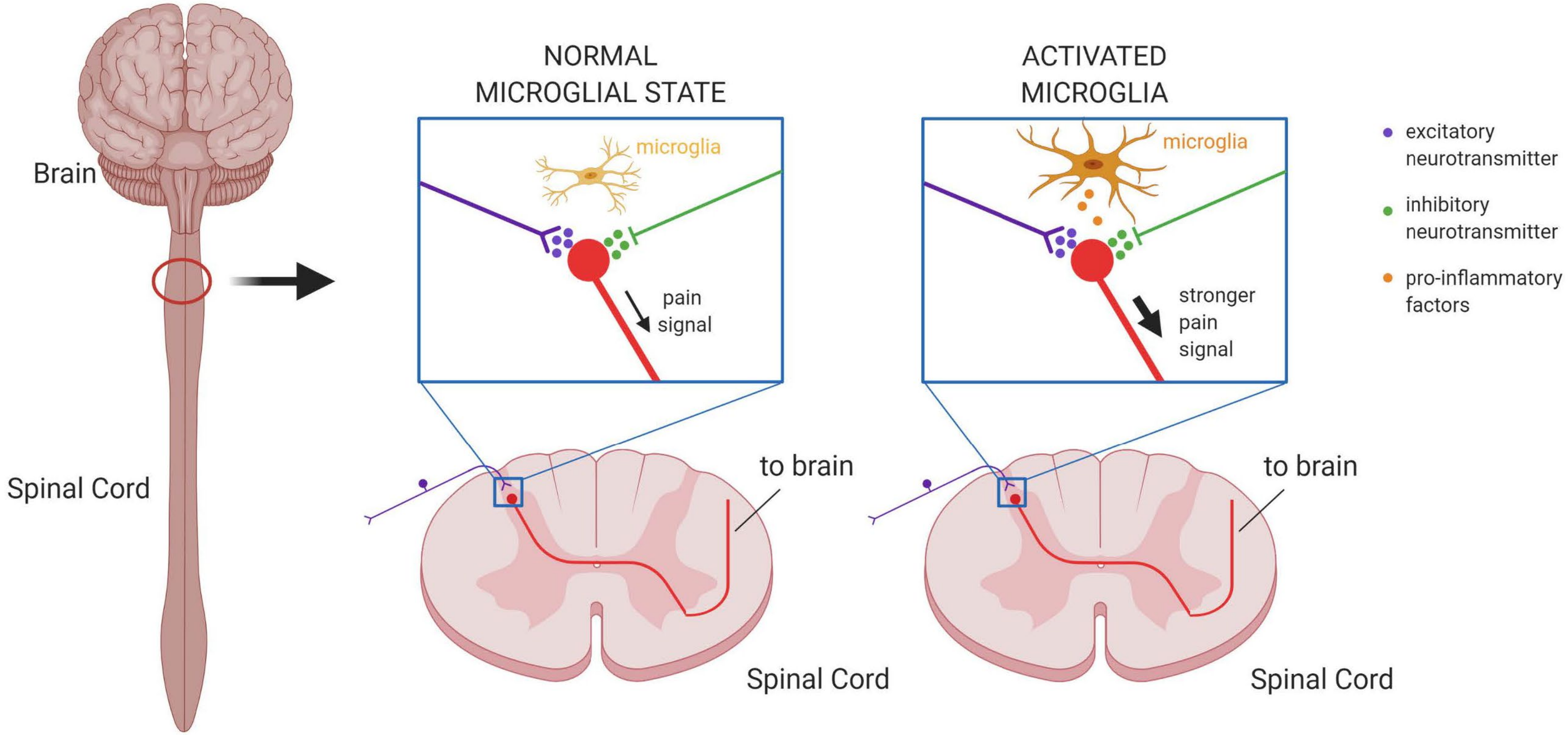

Neuropathic pain – Why does it happen?

4. Maladaptive Neuroplasticity

The nervous system reorganizes after an injury and changes to the structure of neurons and/or neuronal connections called "maladaptive" may occur. For example, neurons that normally provide pain signals can make new connections in areas of the spinal cord and brain, that may lead to the development of neuropathic pain. Neurons can also grow new axonal endings in these areas which also increases the possibility for the development of neuropathic pain.

In summary, both the spinal cord and the brain can become hypersensitive, hyperactive, and reorganized because of the SCI and all these changes can cause neuropathic pain. Therefore, most of the medications that are used to treat neuropathic pain target one or several of these mechanisms. This will be discussed further in the next module.

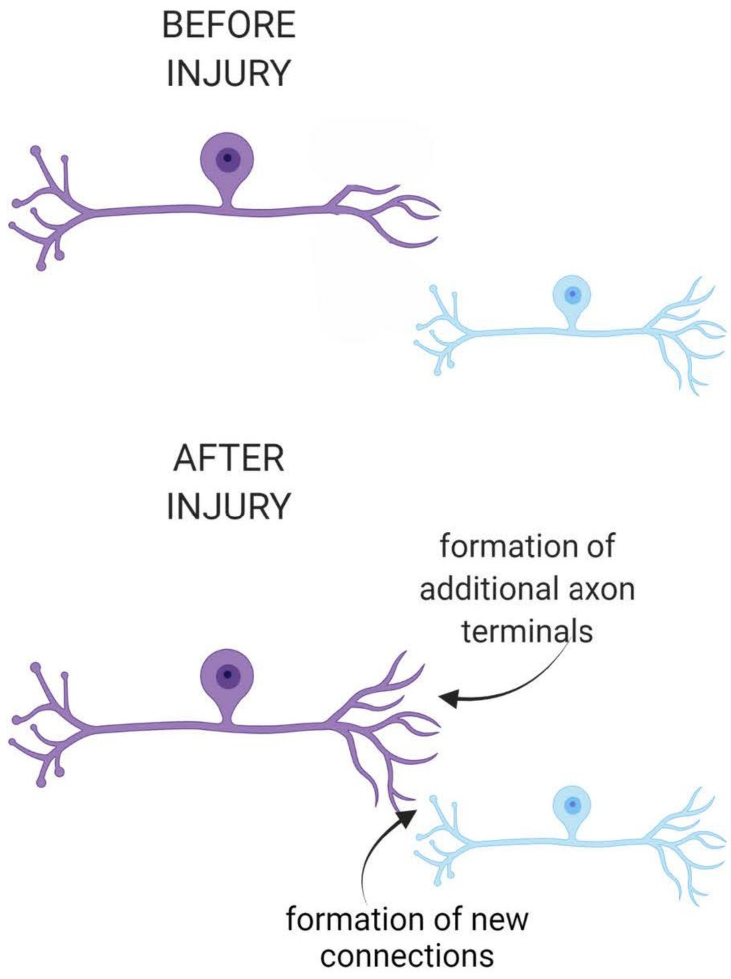

Neuropathic pain – What does it feel like?

“Pressure yes, but not brushing, not poking, it’s deep inside, deep pain.

“The pain there is as if you were to have your legs put into a massive vice grip and you’re constantly squeezing. It’s a very hard throbbing, squeezing pain constantly all day, all night.”

“It’s, intense um it’s not necessarily constant; it does stop…. just like suddenly I got stabbed and I’m like whoa you know, that kind of sensation.”

“The pain just kind of comes…. it’s very unexpected.” “It’s like a total ‘I’m free’ and I’m thinking ‘oh yes the sensation is coming back the nerves are regenerating’ and the next day I’m in excruciating pain.”

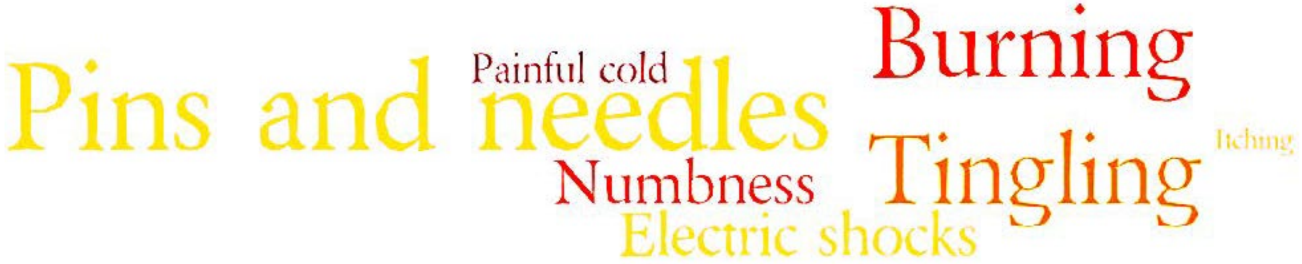

Other words “pricking”, “sharp”, “stabbing”, “shooting”, “lacerating”, “squeezing”, and “aching.”

“If somebody were to just hold my hand, the light touch kills me like I, it’s just, it’s too much, like even now you know if I keep my hands here they’re like a burning fiery so, yeah I don’t do touch.”

“Temperature really affects the way I’m feeling. The colder I am the more miserable I am cause everything gets really tight, the hyper-sensitivity gets worse and things like that.”

“I feel like I have another body connected to my body when I feel the pain.“

“The pain from the nerve pain you know it’s just so crazy you know you can’t, it’s like it can do a 180 in like a second”

“I get confused, cause I thought I pinpointed it and then the pain, it does what it wants to do, I can’t control it, you know it does, it comes and goes”
