# Supplementary material for: Impact of a pain education program for people with spinal cord injury who experience neuropathic pain
Source: Front Pain Res (Lausanne). 2025 May 27;6:1569446. doi: 10.3389/fpain.2025.1569446 (PMC12148921; doi:10.3389/fpain.2025.1569446)

Pain after spinal cord injury

PART 2

Content for Part 2

1. Real-life Case 1
2. Real-life Case 2
3. Real-life Case 3
4. Chronic nature of pain after SCI
5. Impact of pain after SCI - Life
6. Impact of pain after SCI – Activities and work
7. Impact of pain after SCI – Sleep
8. Impact of pain after SCI – Relationships

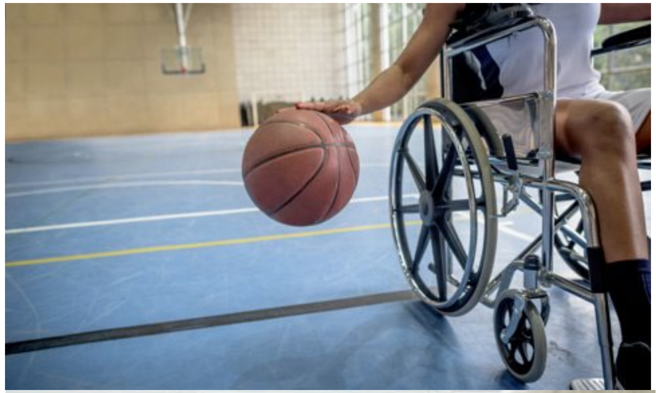

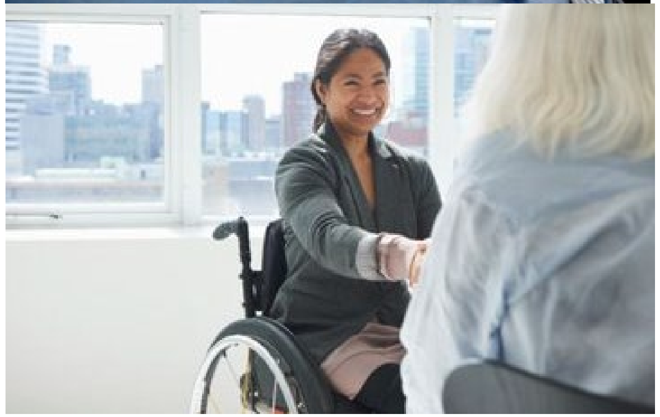

1. Real-life case 1

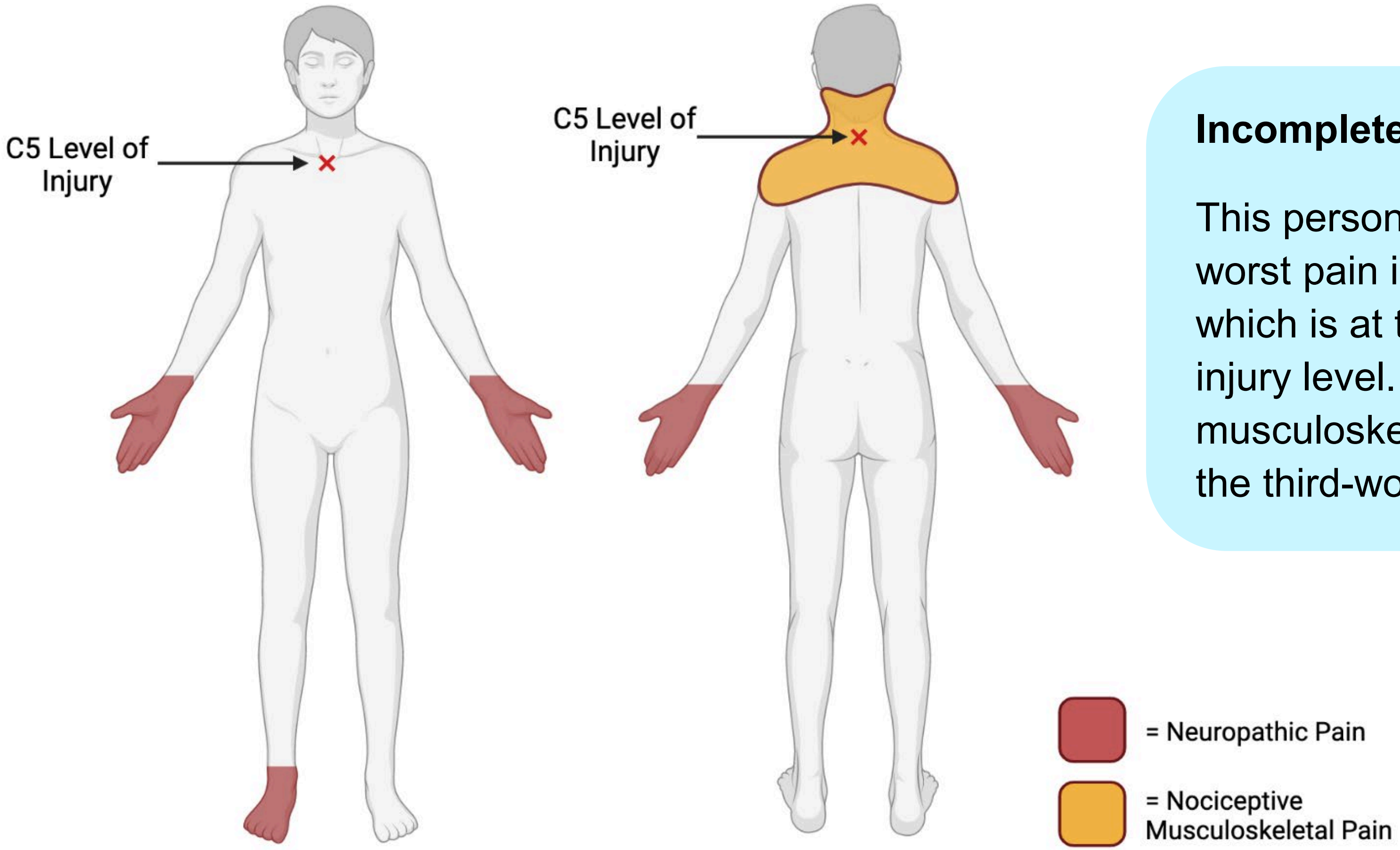

Incomplete (ASIA) C5 Injury level:

This person experiences three different pains. The worst pain is an at-level neuropathic pain in the hands, which is at the neurological level of this individual's injury level. The second worst pain is a nociceptive musculoskeletal pain in the neck and shoulders, and the third-worst pain is a below-level neuropathic pain.

"I have pains in both hands from the wrist down, it's mainly, mainly uh stinging and numbness and also in the right foot. And some pain in the neck but it only comes from sitting in like front of a computer or just being in one spot trying to pay attention, focus on one thing then I have pains in the neck and shoulder."

2. Real-life case 2

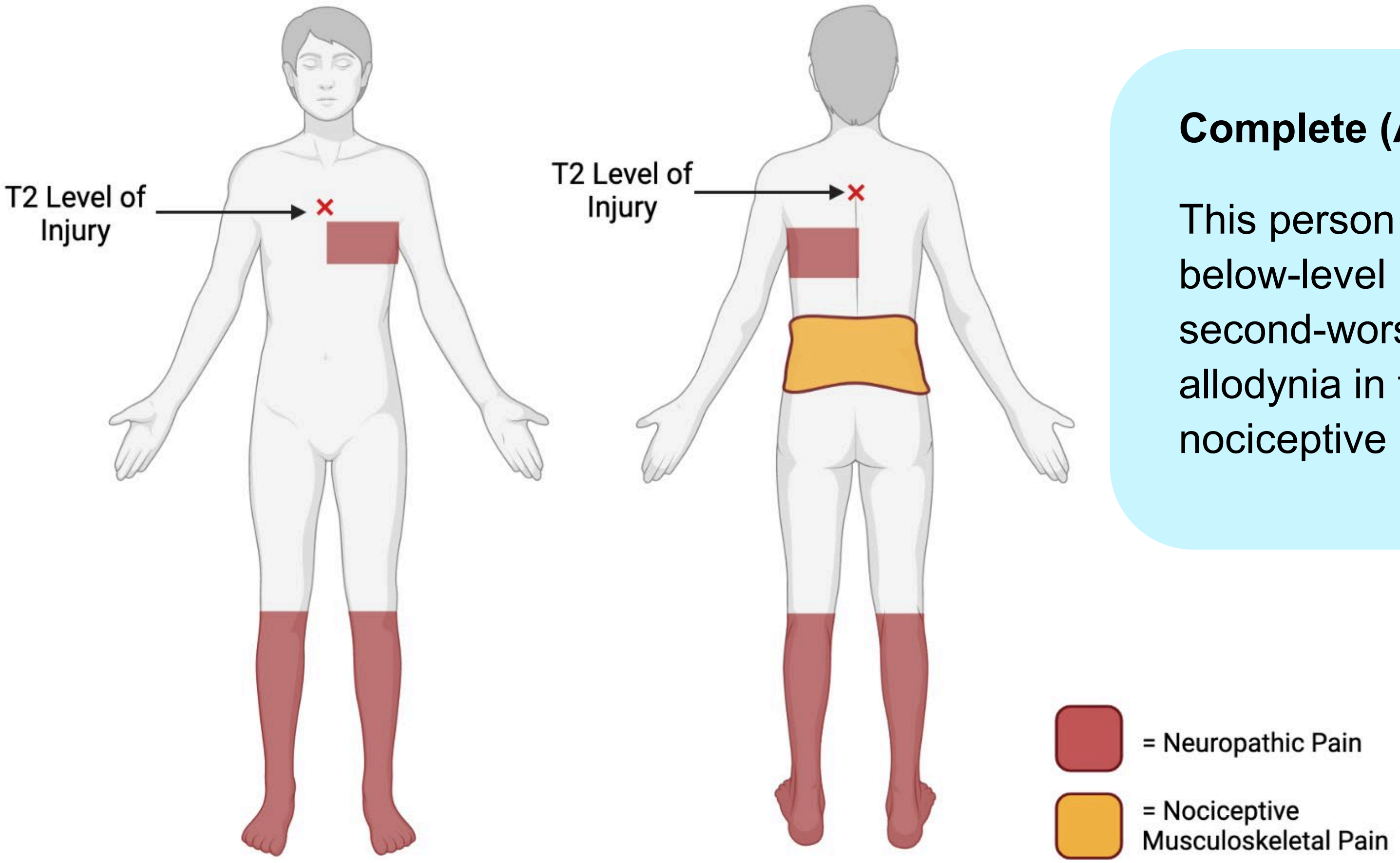

Complete (ASIA) T2 Injury level:

This person has three pain problems. The worst pain is the below-level neuropathic pain in the lower legs and feet, the second-worst pain is the at-level neuropathic pain with allodynia in the chest area, and the third-worst pain is the nociceptive musculoskeletal pain in the lower back.

"Yes, referring to my legs and feet. It's a very hard throbbing, squeezing pain constantly all day, all night. And the difference with my pain in my back is more of a sharp as if you had a knife or a sharp object sticking someone in their back constantly. Now the pain in my left shoulder area right around my chest area is very sensitive to the touch and what I mean by very sensitive: you can take something as light as a bedsheet and if it touches me it produces pain."

3. Real-life case 3

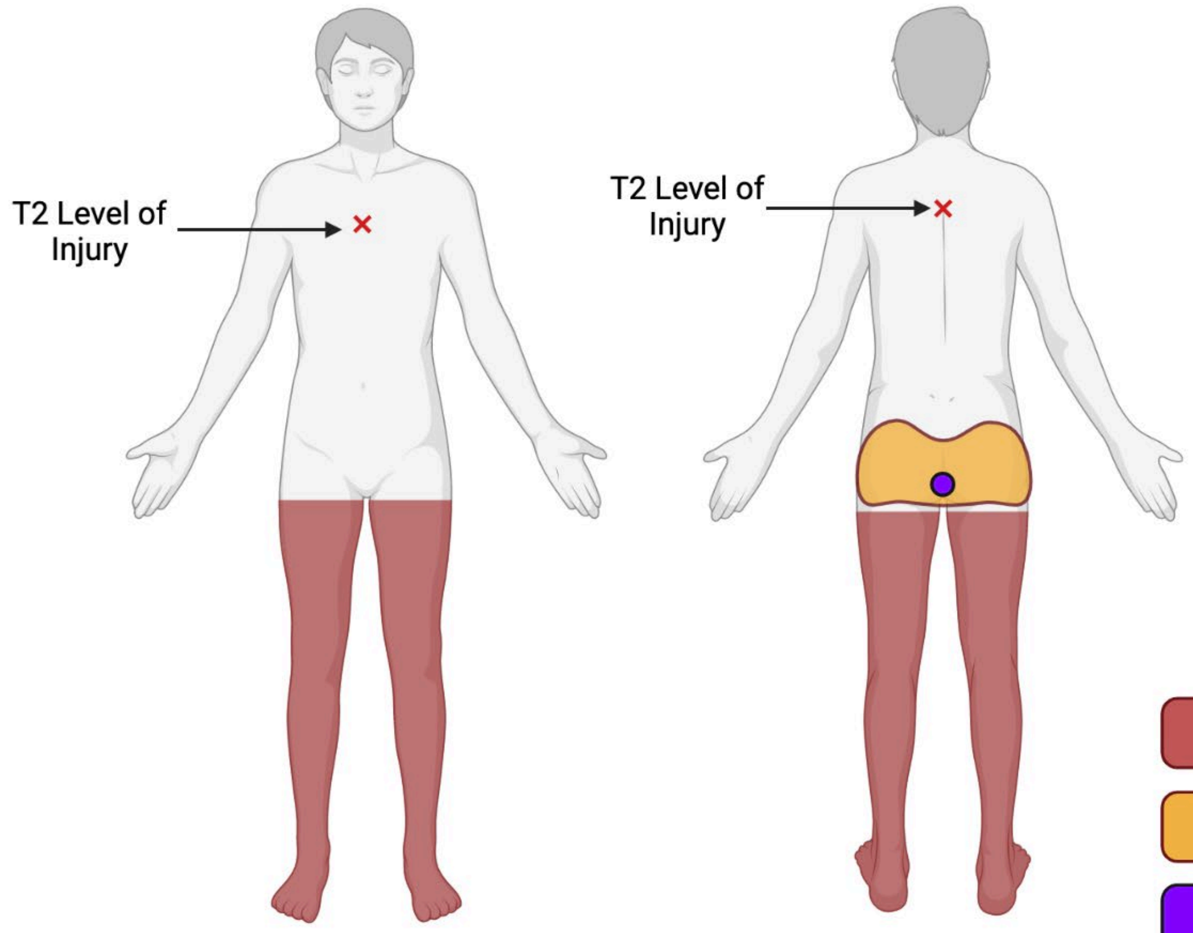

Complete (ASIA) T2 Injury level:

The following person also has 3 pain problems. The worst pain is the below-level neuropathic pain in the thighs, legs, and feet, the second-worst is the nociceptive musculoskeletal pain in the buttocks area, and the third-worst pain is the nociceptive visceral pain in the sphincter area.

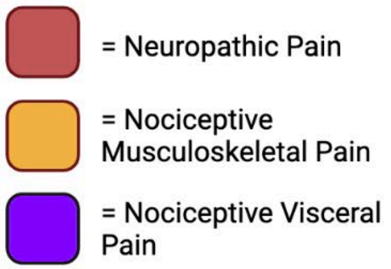

"The legs is, is just the cold, that's the only thing that aggravates my legs is the cold. We talked about the tingling but I think that's cool, it's like little electric shocks going on, and uh I, I, I look at them like they're good feelings things, it's like nerves shooting up or something they don't irritate me or anything".

4. Chronic nature of pain after SCI

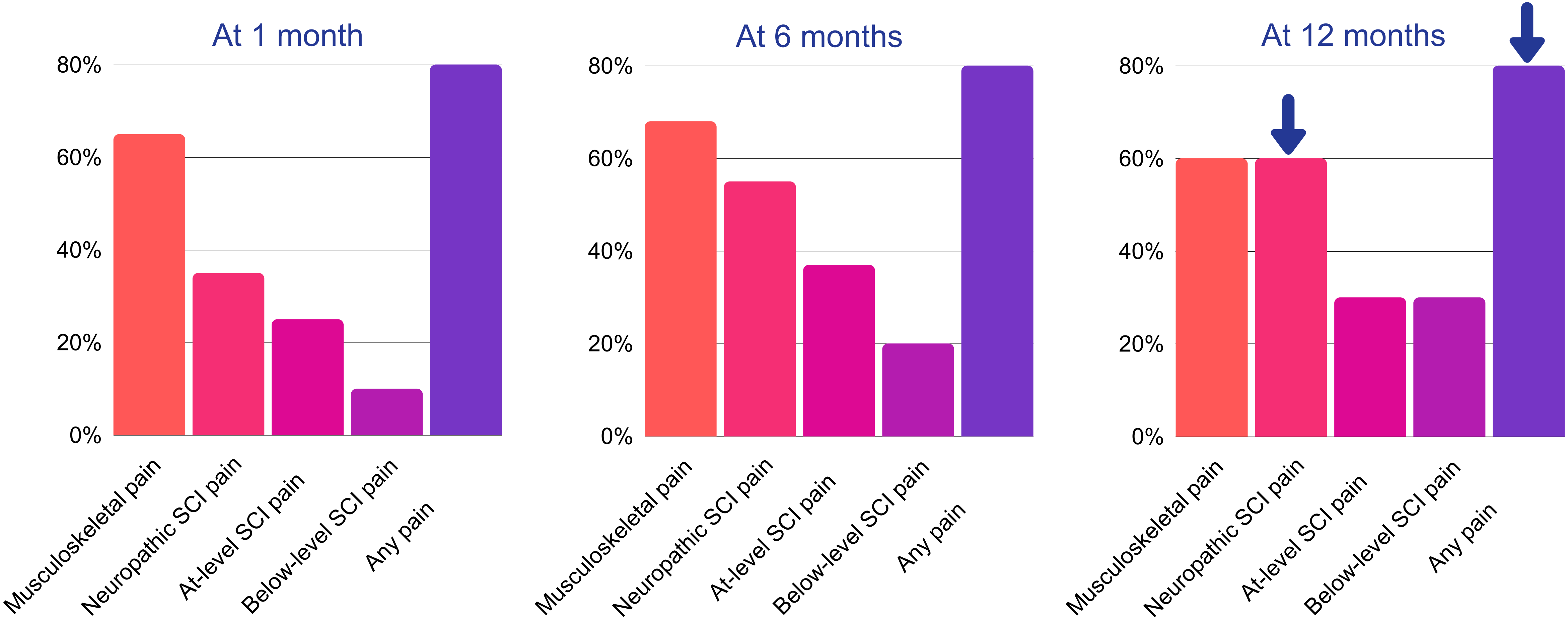

Prevalence of any pain, musculoskeletal pain, and neuropathic SCI pain at 1, 6, and 12 months after SCI

Finnerup, N. B. et al. (2014).

5. Impact of pain after SCI - Life

Persistent pain negatively affects the quality of life and independent living by interfering with sleep, mood, and daily activities. Therefore, these difficult pain conditions may lead to depression, significant psychosocial impact, and reduced quality of life.

“Pain changes everything even if you get good at ignoring it.”

“I think the big thing with spinal cord injury and, and kind of in general and with pain is just not being able to control it.”

“Yes, it affects everything I do. There have been days I wake up and I have to cancel everything”

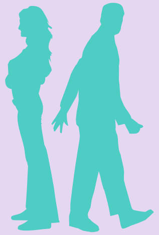

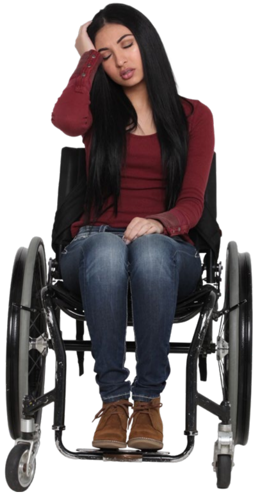

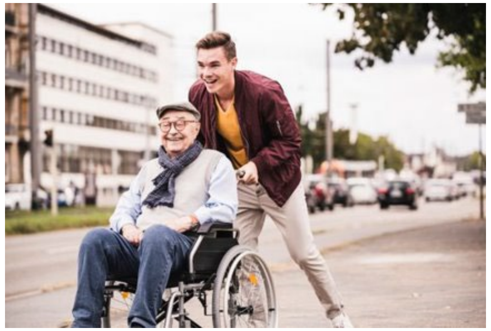

6. Impact of pain after SCI – Activities and Work

About 80% of people living with SCI and chronic pain report that pain interferes with exercise, household chores, work, and other daily activities.

“I can’t.. because if I have the pain I can’t. I like to read, I like to watch movies but if I have the pain it’s just … I get very tense and like I don’t want to, I want my medicine I don’t want anything. Because they don’t understand that kind of pain, it’s like inside, it's inside.”

“Folding laundry is a really big pain… just the motion of leaning in to grab something out of the dryer and then trying to balance myself and fold the clothes…it can really irritate my neck and my upper back and the back and forth motion.”

“There are days where I just can’t leave the house for hours until, sometimes I don’t even leave at all. So yeah I have to reschedule meetings and client visits and stuff.”

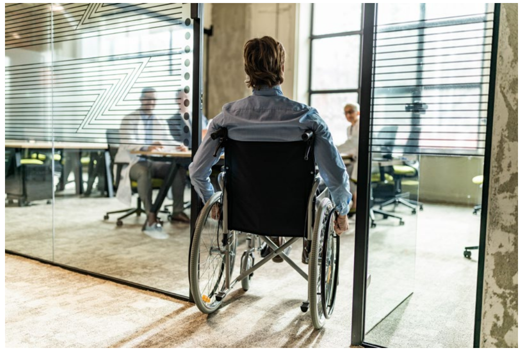

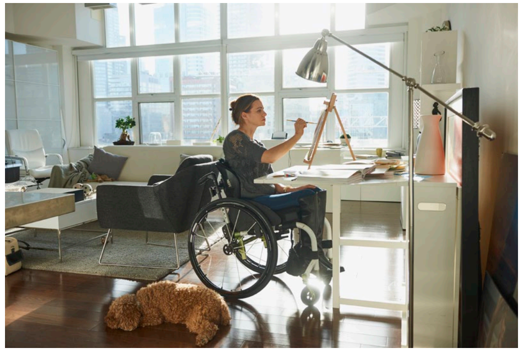

7. Impact of pain after SCI - Sleep

Research studies have demonstrated that pain can interfere with falling asleep, as well as staying asleep. Therefore, some people may feel very fatigued or tired during the day.

"So… sometimes I only get 2 hours or 1 hour. It's hard for me to go to sleep even if I take amitriptyline that's supposed to knock me right out."

"I even have problems I mean going to sleep because the pain is too much so I have to, at the beginning I could sleep all night, all night long but right now the spasticity and the pain wakes me up."

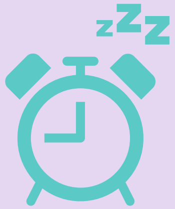

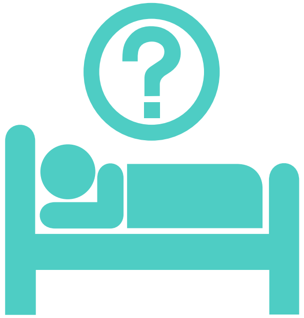

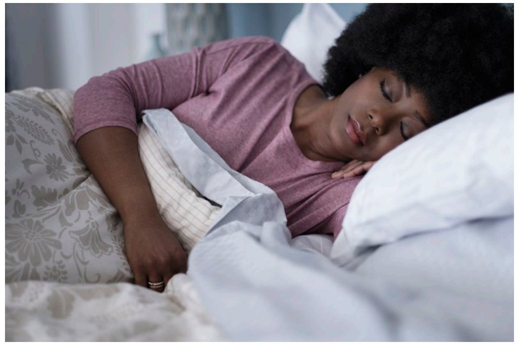

8. Impact of pain after SCI - Relationships

Pain and its impact on sleep, activities, and mood may also have a negative effect on relationships and social participation.

"It worries my significant other, it worries my girlfriend. My kids cry when they see me hurting."

"I don't do anything, I don't go out, I think it cost me my marriage cause I don't, I don't like going out, I don't, I don't like getting touched cause it hurts when I, when so yeah like she fell out of love with me"

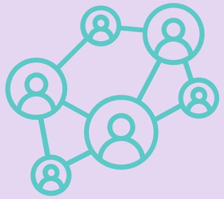

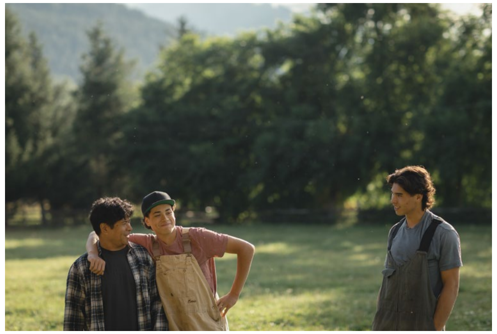

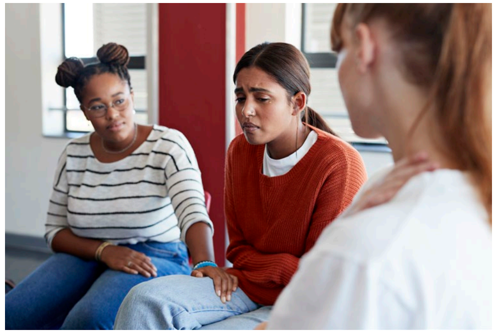

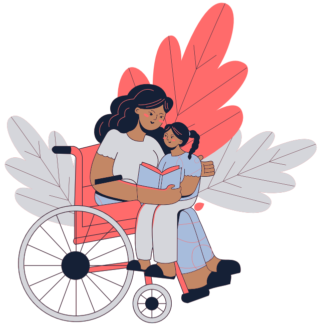

Supplement: Supplementary file 2 [file Datasheet2.pdf]
