# Supplementary material for: Impact of a pain education program for people with spinal cord injury who experience neuropathic pain
Source: Front Pain Res (Lausanne). 2025 May 27;6:1569446. doi: 10.3389/fpain.2025.1569446 (PMC12148921; doi:10.3389/fpain.2025.1569446)
Supplement: Supplementary file 3 [file Datasheet3.pdf]

Pain after spinal cord injury

PART 3

Overview

Many of the pain types that occur after an SCI tend to be long-lasting and can be difficult to treat. However, all pain can improve over time either by itself or because of pain treatment or self-management. Often a combination of different ways to manage pain works best. It is important to discuss your pain treatment goals together with your doctor so that you can find the best combination of approaches that works for you

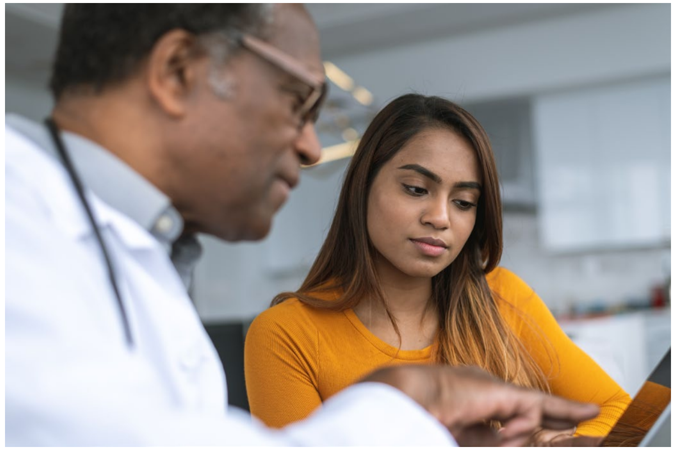

"... I use the oven analogy. I tell folks that I can't turn off the oven, I'm just turning down the heat a little bit."

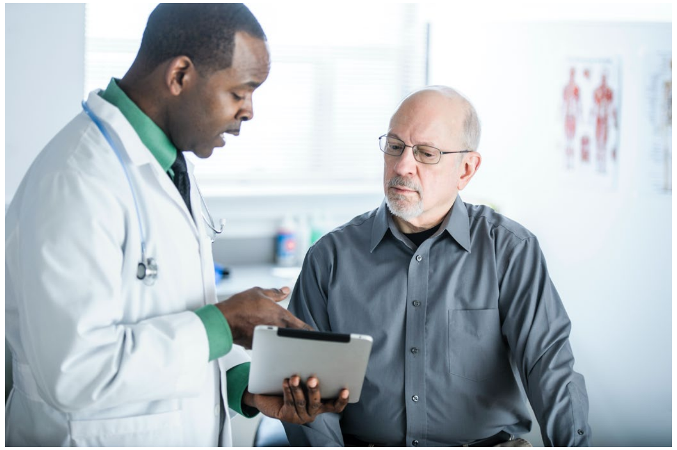

Content for Part 3

1. **Benefits of a better understanding of pain and how to manage it.**
2. **What do people do to manage their pain?**
3. **Evidence based treatments (pain medication and other)**
4. **Opioid perspectives**
5. **Pain medication perspectives**

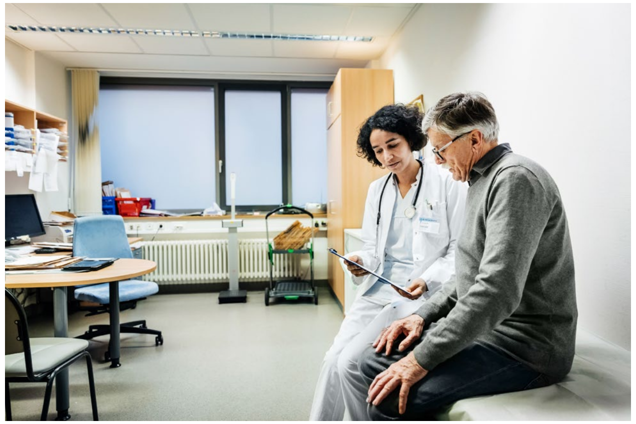

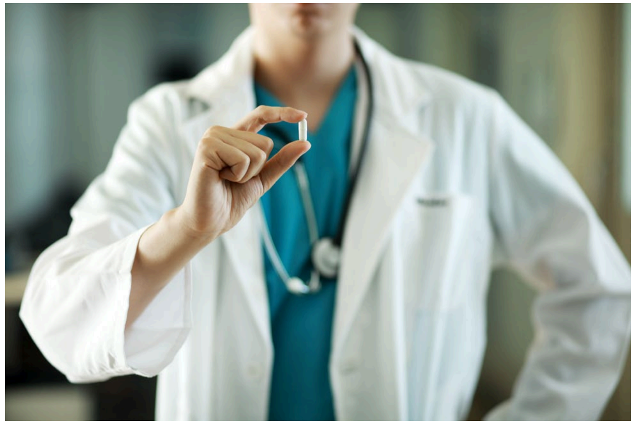

1. Benefits of a better understanding of pain and how to manage it

All people with SCI who experience chronic pain need to know their own pain and what kinds of treatments and other approaches that can be used to make their pain more manageable. A good understanding of your pain can help you to communicate better with significant others and family members, and healthcare providers regarding your pain. It can also be very helpful to learn from other people with SCI how they manage their pain and what their experiences are.

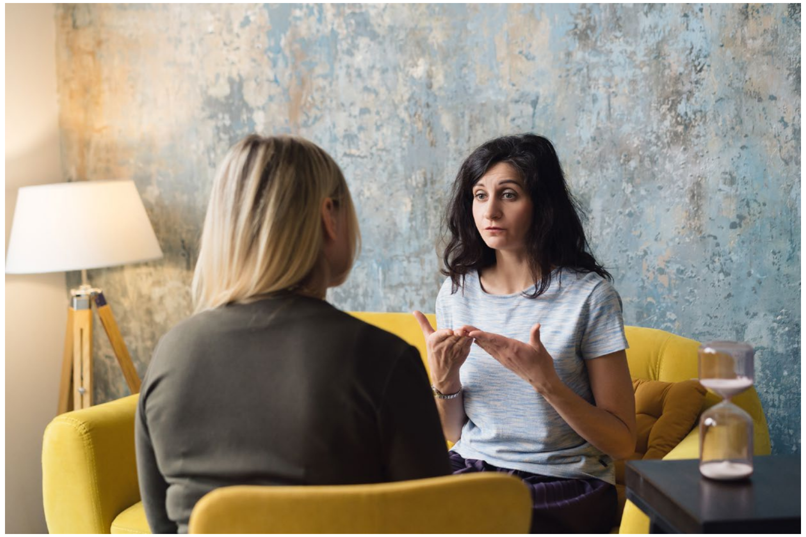

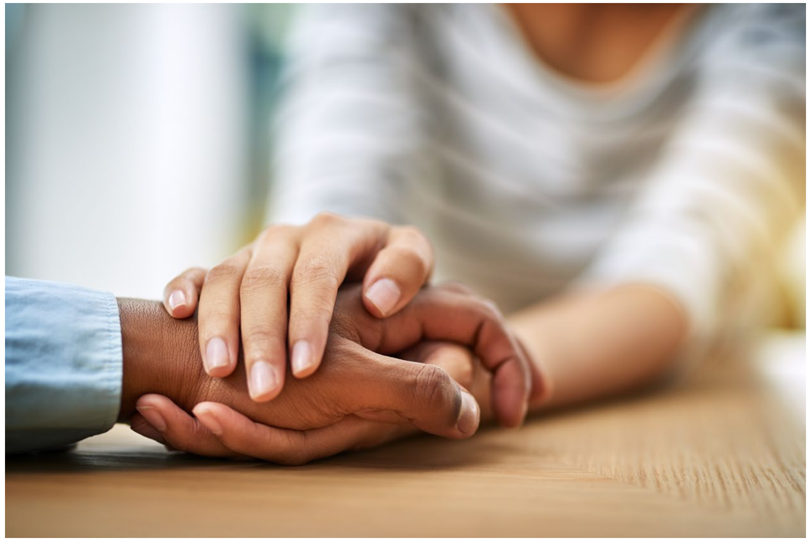

2. What do people do to manage their pain?

People who experience persistent pain after their SCI often use different combinations of medications, self-management, and physical and mental strategies to deal with their pain.

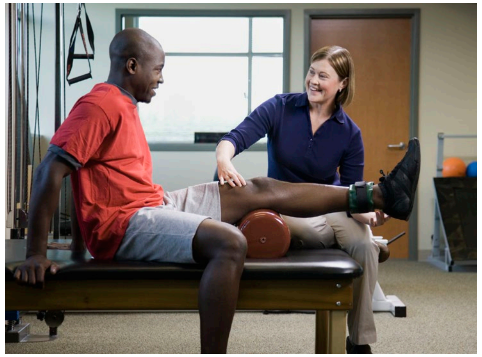

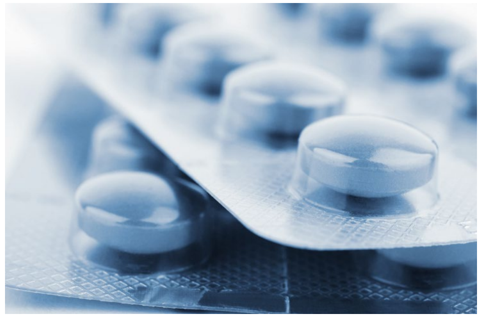

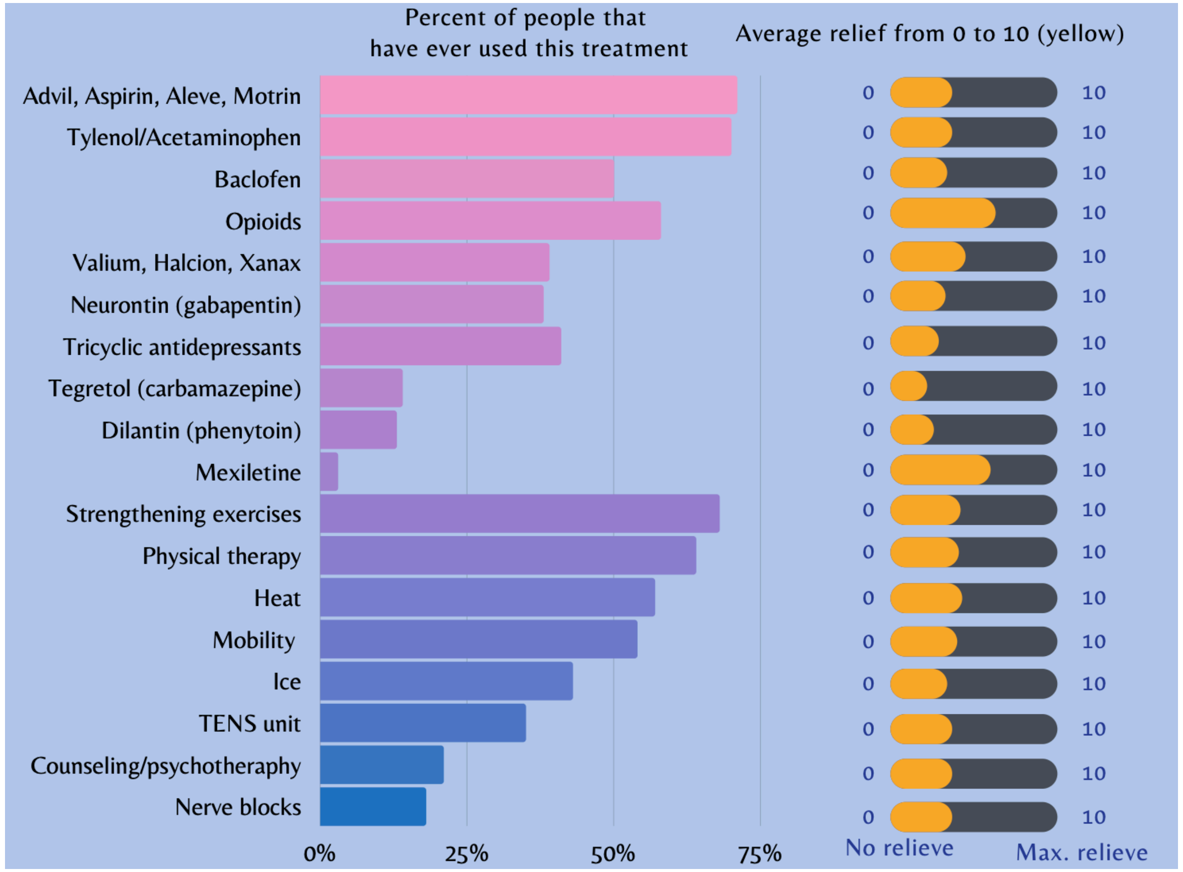

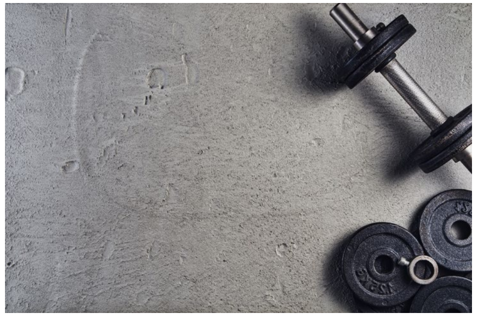

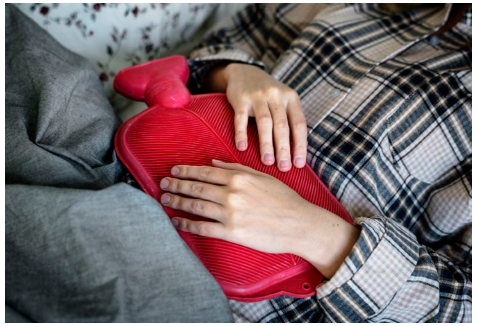

Cardenas, D. D., & Jensen, M. P. (2006). Treatments for chronic pain in persons with spinal cord injury: A survey study. The journal of spinal cord medicine, 29(2), 109–117. https://doi.org/10.1080/10790268.2006.11753864

3. Evidence based treatments

To decide if a treatment is effective in relieving pain, research studies investigate how effective the treatment is in decreasing the intensity of pain compared to a placebo (fake) treatment. If the treatment is more effective than the placebo, it provides an “evidence” for the treatment.

Some studies also measure other important pain-related factors, for example, levels of depression, activity, or sleep to understand the additional effects of the treatment.

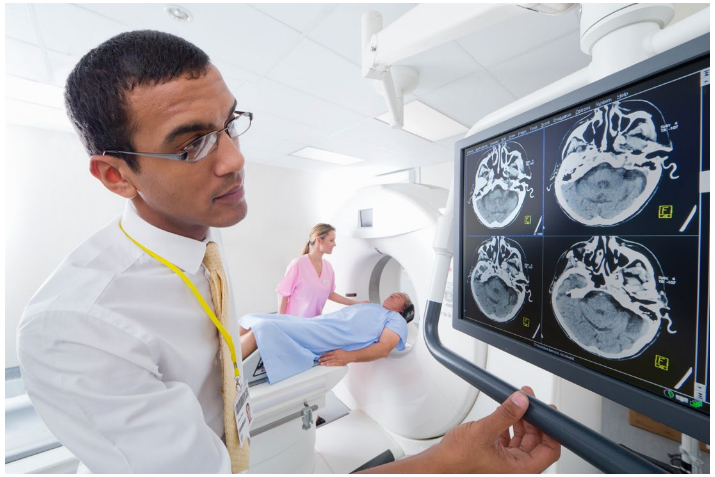

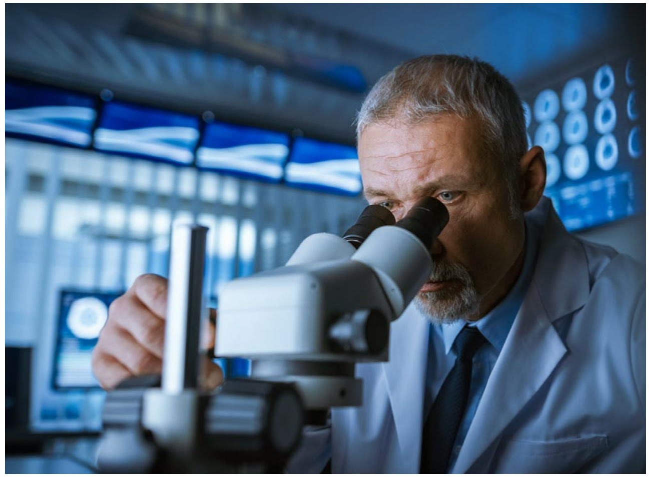

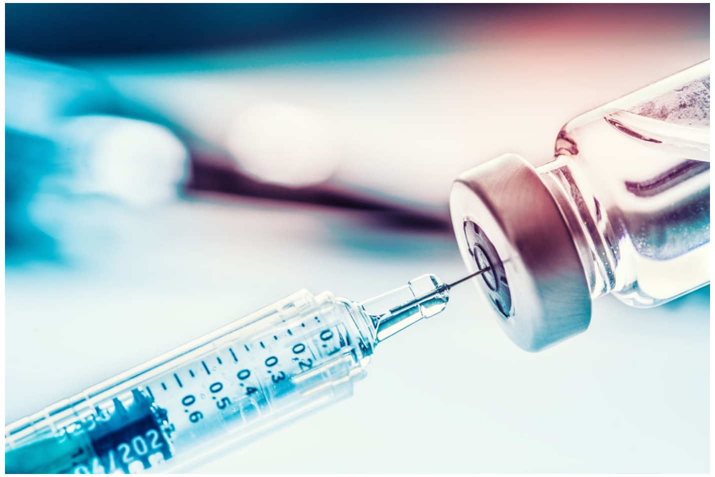

First line: Pregabalin, Gabapentin

Pregabalin (Lyrica) First Choice

Will it work?
Several research studies that used pregabalin to treat neuropathic pain showed that it significantly reduced the intensity of pain. Most studies used flexible doses between 150-600 mg per day. It is important to keep in mind that even the most effective medication does not work for everyone.

How it works?
Pregabalin can decrease the hypersensitivity of nerve cells by reducing the number of excitatory neurotransmitters that the neurons release.

Side effects:
The most common side effects are mild to moderate temporary drowsiness and dizziness, but other less common side effects such as weight gain and limb swelling are also possible.

Gabapentin (Neurontin) Second choice

Will it work?
Gabapentin is used when pregabalin is not an option. Some studies in people with SCI found no effect while others showed a significant reduction of neuropathic pain using doses between 1800 and 3600 mg per day.

How it works?
Like pregabalin, gabapentin can reduce the number of excitatory neurotransmitters that neurons release.

Side effects:
Drowsiness and dizziness, but other less common side effects such as weight gain and limb swelling are also possible.

First line: Amitriptyline, Duloxetine

Amitriptyline (Elavil) Third choice

Will it work?
Amitriptyline is another less common first-line medication that can be used if the previous two alternatives were ineffective. Studies have had mixed results, but overall, the studies suggest that amitriptyline could be beneficial. Studies used doses between 25 and 150mg per day.

How it works?
Amitriptyline is a tricyclic antidepressant. It has many effects, but the reduction of neuropathic pain is thought to be due to increasing the effectiveness of the body's own pain inhibitory system by keeping the brain and spinal cord levels of the neurotransmitters noradrenaline and serotonin elevated.

Side effects:
Possible side-effects include drowsiness, dry mouth, irregular heartbeat, and an increased risk for seizures for those with epilepsy.

Duloxetine (Cymbalta)

Will it work?
The typical starting dose is 60 mg a day, which may be increased to twice a day.

How it works?
Duloxetine is another antidepressant known as a serotonin- norepinephrine reuptake inhibitor (SNRI). It is believed that improved mood due to the increase in serotonin and norepinephrine can help regulate feelings of pain.

Side effects:
Some potential common side effects include dry mouth, headache, constipation, drowsiness, nausea, difficulty sleeping, and decreased appetite.

Other options (Pharmacological):

Oxcarbazepine (Trileptal, Oxtellar XR)

This medication may reduce electrical, burning, and pricking pain, numbness, allodynia, and pressure analgesia.

How it works?
Some studies show they reduce hyperactivity in the brain and the spinal cord.

Side effects:
Similar to gabapentin and pregabalin like drowsiness and dizziness, but other less common side effects such as weight gain and limb swelling are also possible.

Tramadol (Ultram, Ultracet, Ultram ER, Ultradol, ConZip)

Tramadol could be tried after “first-line” medications have not provided satisfactory pain relief. The maximum dose is 400mg/day.

How it works?
Tramadol increases pain inhibition by opioid-like mechanisms and by keeping the brain and spinal cord levels noradrenaline and serotonin elevated.

Side effects:
Common side effects are drowsiness, nausea, and constipation. Tramadol is a controlled substance in the US and there is a risk for addiction.

Lamotrigine (Lamictal)

Another option for neuropathic pain in individuals with incomplete SCI. The maximum dose of lamotrigine in the study was 400 mg/day.

How it works?
The primary action of lamotrigine is decreasing hypersensitivity in neurons by reducing the number of open ion channels.

Side effects:
Dizziness, drowsiness, headache, and rashes can be common side effects.

Other options (Non-Pharmacological):

Botulinum toxin A
Botulinum toxin A injections are widely used for reducing spasticity because this compound decreases muscle activation. Because people with spasticity can also experience pain, Botulinum toxin A has been used for below-level neuropathic pain with injection in an area with maximal pain.

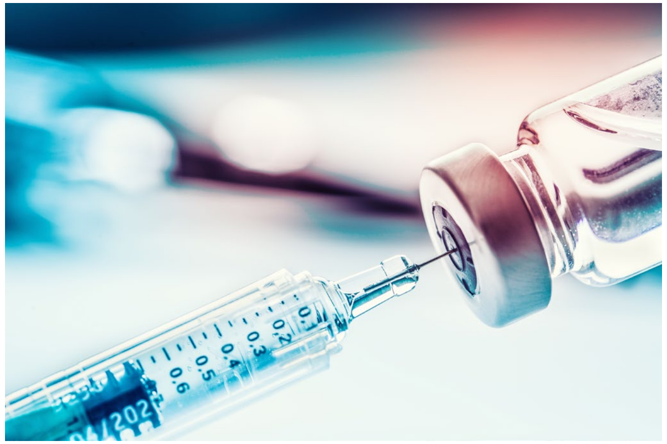

Combined visual illusion and direct current stimulation (tDCS)
Transcranial direct current stimulation has also been combined with visual illusion. Visual illusion is a way to trick the brain into seeing something that is not really there for example by using mirrors. A study found a significant reduction in pain intensity after a combined visual illusion and tDCS in people with neuropathic pain. Side effects include mild headache and fatigue.

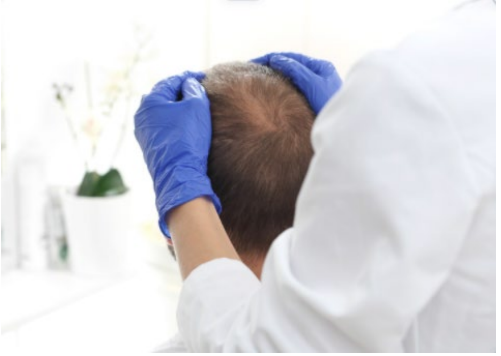

Transcranial direct current stimulation (tDCS)
Transcranial direct current stimulation uses constant, low direct current delivered via electrodes on the head and is a way to influence nerve signals in the superficial layers of the brain. People with neuropathic pain after SCI may experience a reduction in pain, specifically in pains that are continuous or attack-like. Side effects may include skin irritation and seeing a brief flash of light when the electrode is placed near the eye.

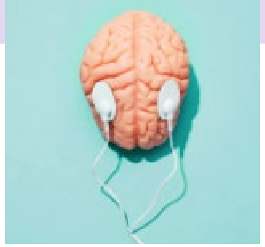

Other Options (opioids): Oxycodone

How it works:
Oxycodone is a potent opioid that increases pain inhibition.

Side Effects:
Dizziness, drowsiness, headache, and rashes were common side effects in studies.
With opioids, there are risks of addiction, abuse, and overdose.

Many of the side effects associated with long-term opioid therapy can negatively impact an individual’s quality of life. Side effects include opioid-induced constipation, increased pain, lower levels of male sex hormones, sleep disturbance, physical dependence.

Patients may also become tolerant to their usual dose of opioids, causing a decrease in the medication’s ability to treat pain.

Opioids are an effective and important tool for chronic pain treatment, but because of the risks, the use of opioids need to be discussed between the patient and the provider and its use monitored.

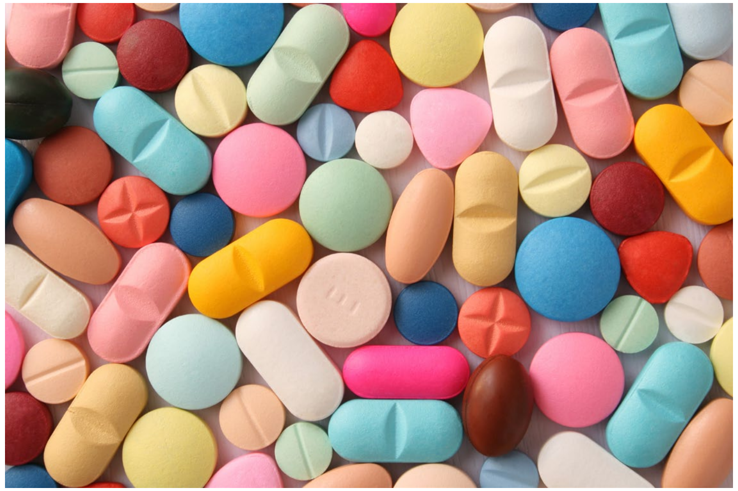

Opioid Perspectives: People with SCI

“I've used medications for the first seven, eight years, then that's when I asked to get different help, because I didn't like what they were doing to me. It felt like I was brain dead. They had me on Fentanyl, they had me on stuff. They had me on.. Oxycontin, Roxicet. They had me on... a lot of morphine. Everything you can think of, I've been on it.”

“I've tried many, many... forms of.. pain meds and whatnot; and I'm still on... pain meds. But one of the things that I noticed that everything that, my capabilities were limited and what I could do. And when the pain meds wear off, I become more irritable. I become short tempered. It has affected my social life.”

”They put me on methadone and it worked great. In fact, methadone is probably the best thing that I've had for all my pain. It's longer lasting thing, longer acting, and then for some reason, I don't know why, they decided to take me off of it.”

“I used to take half the amount or a quarter the amount before to get some relief now three, four times the same medicine is barely enough. So I don’t know if that’s because I’m developing more tolerance to the medicine or because my pain actually increased.”

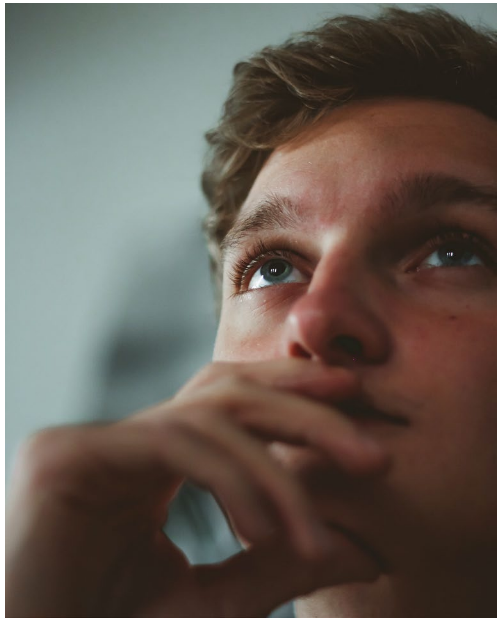

”I was taking Oxycontin twice a day so I would always be sleeping, I would always be on drugs and it affected my life because my children they didn’t even want to see me, I didn’t care about anybody just the pain to go away I just want to be, take the pain away so they would give me the drug and that was it.”

Opioid perspectives: Family

“I just think educating people on the fact that narcotics don't help neuropathic pain. I mean, we see so many people addicted to them all the time and especially people, like, in the military. I mean, they just throw them at them and, and I'm like, ‘This doesn't help. It makes things worse.’”

“This is every day and I think he takes it every four hours or three hours or something. So, how effective is it and you know, how long? Some meds might be the effect, you know. Would they switch him up on medication and then how long will that work? So, that's my biggest thing that.... will the pain ever go away? Can he be treated or can he be, you know, no more lies or ..”.

“I don't like that he takes medicine because when he starts with the pills, and at the beginning, that's okay, but after two months, he needs more, and more and more“

"You know, well, you have that drug in you, but is it gonna help?" And, uh, he says nothing really helps and, like, the Lyrica, they give him all kinds of side effects. You don't know which medication is doing the side effects”.

“Pain medication, when it, when she, like, say if two weeks go by, her body gets used to it, then sometimes it, it doesn’t help sometimes”

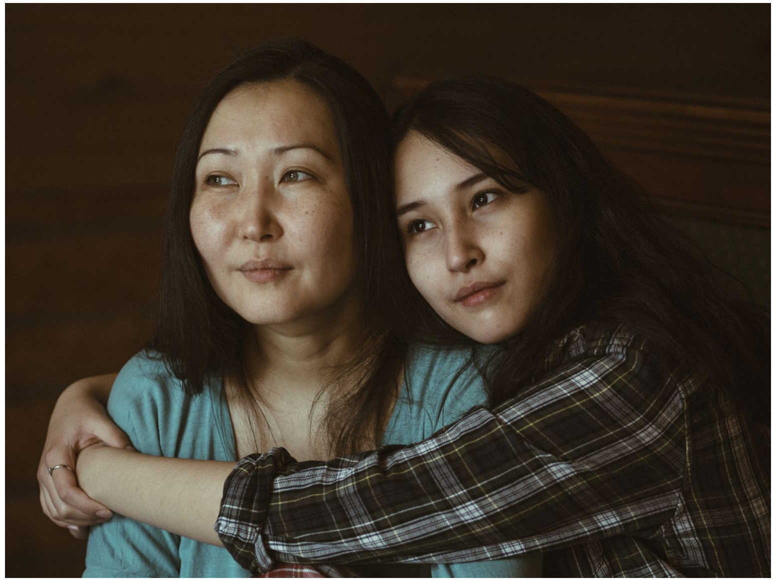

Opioid perspectives: Healthcare providers

"Giving guys two Percocet four times a day and his drug screen was never completed -we do a practitioner database query, to look and make sure they're not getting opiates from any outside providers, or the state"

"Neuropathic pain does respond to opiates, for a while. But then you have to end up having to increase your dose and then at a point you can't you don't want to increase your dose anymore and you have to change. You have to convince the patient to change....... and that can be difficult."
"We know that opioids are not the right choice"

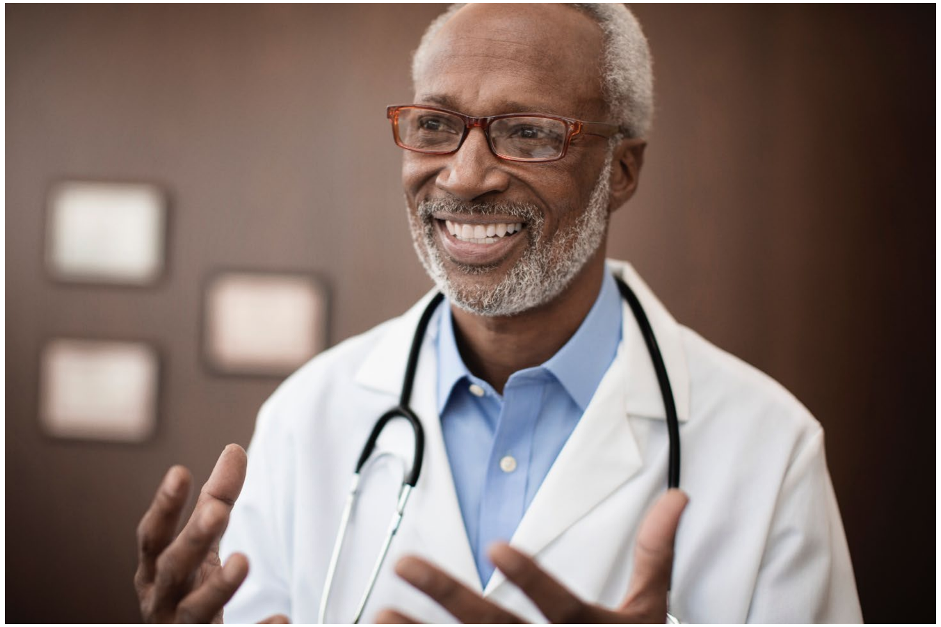

Opioid perspectives: Healthcare providers

“Some patients state that, "This is the only medication that seems to work." ... And that may be because they had tried out other medications.”

“But for those who have severe pain, ...trying a bunch of different regiment....even the opiates don't necessarily help.”

“So I think the providers need to-we need to all to start to practice the same and use the guidelines that are.... coming from the feds, the surgeon general's, plan-guidelines for how we need to manage. And a number of organizations have come out with opiate prescribing guidelines.”

“Doctors are really concerned...they don't want to be accused of something that they thought they were helping“

“Most of them have been on opioids...and this is kind of the challenge that I think, they are kind of facing, because at the beginning you do more acute pain management and they are into those kinds of medication, and after that, it's ... difficult to make them understand that sometimes opioids are not the right thing to do for neuropathic pain.”

“We know that opioids are not the right choice”

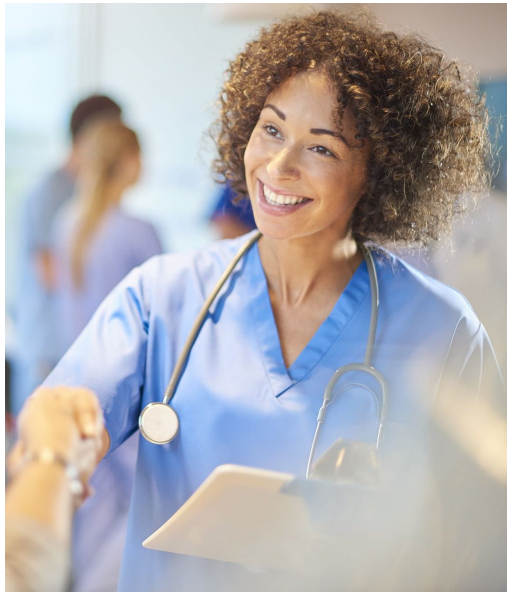

Other options: Cannabinoids, Marijuana/cannabis perspectives

Cannabinoids have the potential to affect multiple central nervous system targets, thus more recently it has been of interest for the treatment of pain and other neurological disorders. Studies have found varying results in the treatment of SCI-related neuropathic pain and more investigative research is needed, however, individuals with SCI have reported lower pain levels while using cannabinoids. One study found that the intensity of SCI-related neuropathic pain was significantly lower after vaporized cannabis (2.9%, or 6.7% THC) compared to placebo. Another study did not find differences between synthetic THC and placebo.

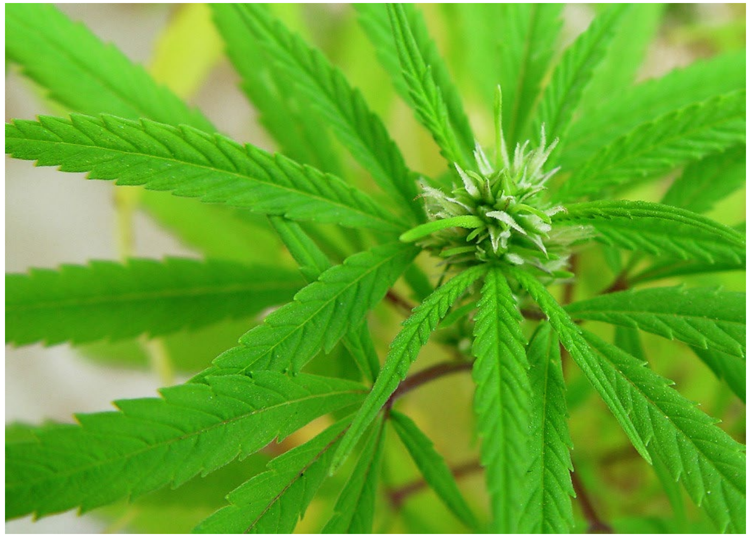

This Photo by Unknown Author is licensed under CC BY-SA-NC

"You can get paranoid, the rest of it, and your body still relaxed but your mind is not quite with it… so I don't like that side of it, that's what I hate, I hate not feeling in control… Like if I was to smoke it I wouldn't want to go out into the community."

There's some [high CBD cannabis] out there that'll relax your body, which is the one that helps me relax my mind, get out of that state of pain, or there's the one that's makes you go to sleep [high THC]. Those are the two simple breeds really. I'm fortunate sometimes to get the one that makes me live through the day and not sleep through the day.

"Well look, I've got nothing to lose to try it and I'm not getting high from it so, what have I got to lose? And it, it does a better job than the stuff that's legal."

Bourke JA, Catherwood VJ, Nunnerley JL, Martin RA, Levack WMM, Thompson BL, Acland RH.Using cannabis for pain management after spinal cord injury: a qualitative study. Spinal Cord Ser Cases. 2019 Oct 8;5:82.

Last resort options: dorsal root entry zone procedure (DREZ)

This approach is used for exceptional circumstances and as a last resort due to its invasive nature. DREZ procedure involves a neurosurgeon creating an opening in the spine to isolate an area of spinal cord damage to silence hyperactive nerve cells by creating lesions within the back part of the spinal cord. The goal is to decrease the number of pain signals.

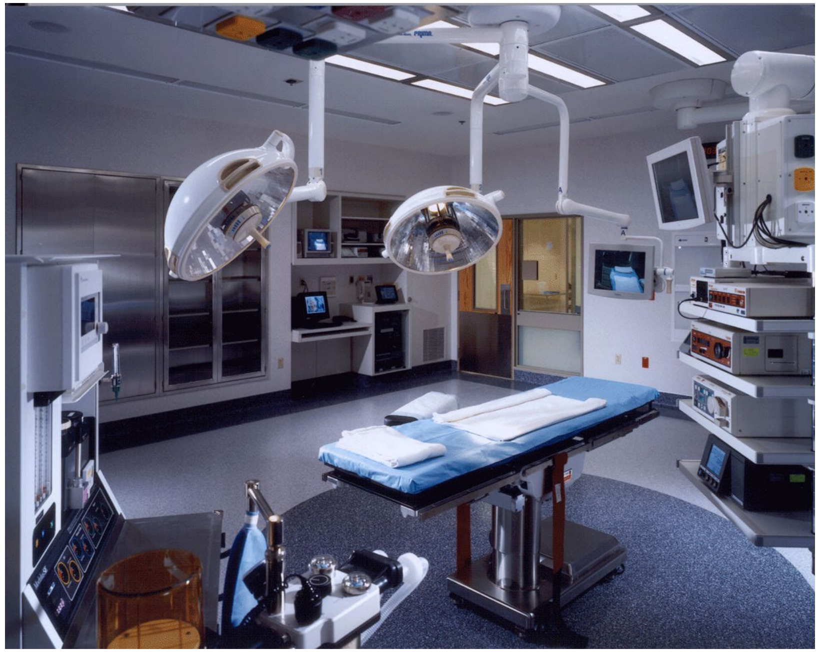

This Photo by Unknown Author is licensed under CC BY-ND

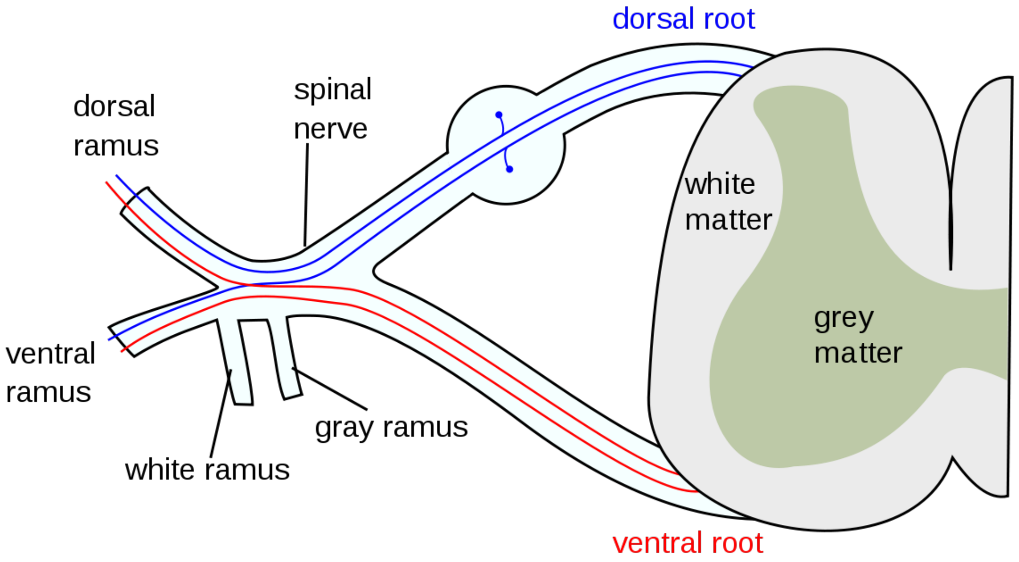

This Photo by Unknown Author is licensed under CC BY-SA

The pains that occur after SCI vary widely in where they are felt, what they feel like, how they respond to treatments, and how they affect your quality of life.

Pain medication perspectives: People with SCI

"Lyrica does help me"
"Aleve for me is wonders"
"I take the pain killers when I need to"
"The medicines I use, sometimes work, sometime don't" "It doesn't take it all away, but it does help"
"I take different pain medications"

"It didn't really help me"
"The way they tell you to deal with pain is, uh, medication"
"They just throw some drugs at you and, you know, say, "Try this and see how you do""
"The doctor that I see at the clinic, he doesn't really talk to me about pain. I- I'll tell him what's going on and he usually just throws another pill at me”

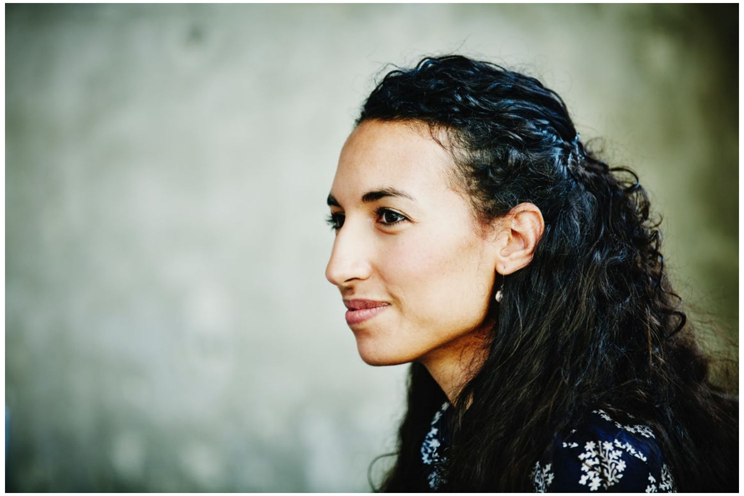

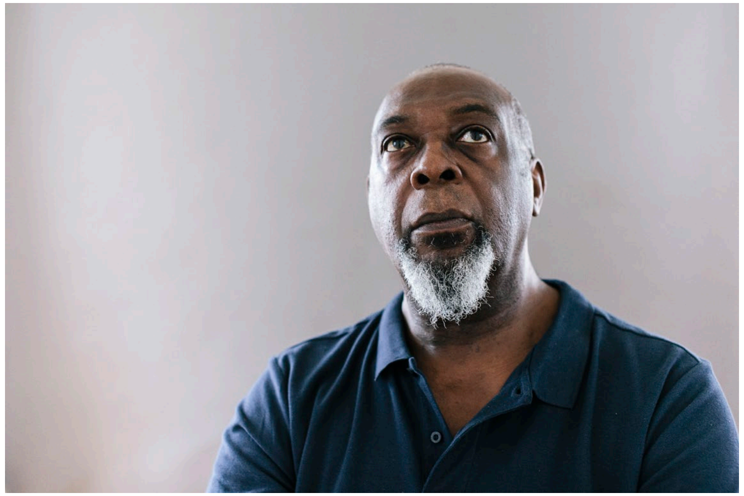

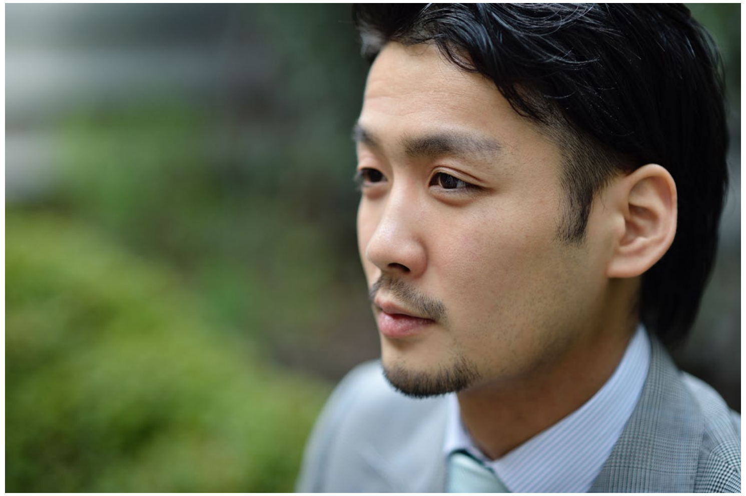

Pain medication perspectives: Healthcare providers

Every medication will carry a side effect, if not multiple side effects"

"Quite a few patients do not want to be on the medications”

"Find a good balance between medication and side effects can be tough at times"

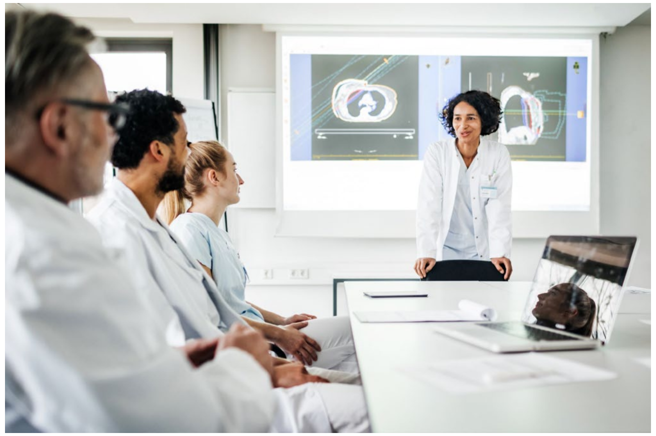

”There's clearly an ability of the medicines to actually reduce pain and .... the physical therapies, and the tens units, all of these things together to reduce pain”

"Because the nerves are smarter than the drugs, tolerance develops over time and so we're increasing doses and then we end up having to switch"

"Gabapentin, Pregabalin, and those are the best in efficacy by far. The anti-depressant class of medications is the next class and they're okay"

"We need more treatment options"

QUESTIONS?

OR COMMENTS
