# Supplementary material for: Impact of a pain education program for people with spinal cord injury who experience neuropathic pain
Source: Front Pain Res (Lausanne). 2025 May 27;6:1569446. doi: 10.3389/fpain.2025.1569446 (PMC12148921; doi:10.3389/fpain.2025.1569446)
Supplement: Supplementary file 4 [file Datasheet4.pdf]

Pain after spinal cord injury

PART 4

Overview

In our previous study, over 60% of people with SCI who experienced chronic pain strongly agreed that they would like more information about different treatment options for their pain including alternative methods. More than 30% strongly agreed that they would prefer another treatment than medication. In our previous study, over 60% of people with SCI who experienced chronic pain strongly agreed that they would like more information about different treatment options for their pain including alternative methods. More than 30% strongly agreed that they would prefer another treatment than medication.

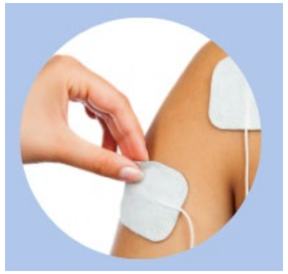
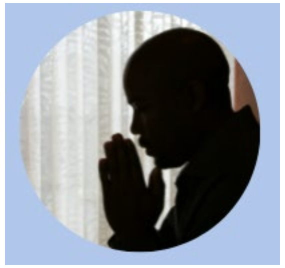
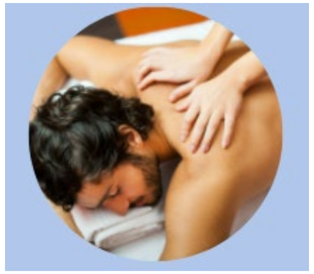
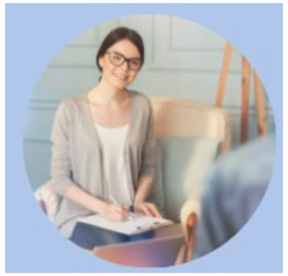
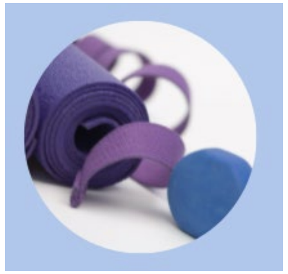

Content for Part 4

1. **Non-pharmacological Treatments and Self-management**
2. **TENS, Exercise & Activity, Rest & Physical Therapy, Massage and Acupuncture, Yoga, Relaxation, Mindfulness and Meditation, Mental Strength, Distraction and Companionship, Spirituality, Hope and Optimism**
3. **Learning, Planning, and Anticipating**
4. **Avoiding Triggers**
5. **Anxiety and Depression**
6. **Significant Others, Family Members, and Caregivers**
7. **Final Message**

1. Non-pharmacological Treatments and Self-management Overview

Recent recommendations for how to manage neuropathic pain after SCI often encourage approaches that combine self-management with both pharmacologic and non-pharmacologic methods. Such combined pain management strategies may also include patient education, cognitive behavioral therapy, self-management strategies, physical or relaxation exercises. These approaches aim to reduce pain, improve coping skills, and reduce pain interference with activity, sleep, and mood.

The most common additional treatments used by people with SCI to manage their pain are the following:

- Transcutaneous Electrical Nerve Stimulation (TENS)
- Physical Therapy
- Massage
- Acupuncture
- Heat/Cold Therapy
- Exercise

2. Transcutaneous Electrical Nerve Stimulation (TENS)

Transcutaneous Electrical Nerve Stimulation (TENS) uses a device with electrode adhesive pads to send low voltage electrical currents near or at the nerves.

TENS is often felt as a pricking/tingling sensation at the site, however, it is not painful and the current can be adjusted to the individual's desired level.

In several research studies, TENS has been shown to decrease pain in some people with SCI. TENS can be performed by a healthcare professional, or at-home with affordable kits that are available for purchase.

Healthcare provider: “ (TENS) may be underutilized... I can't say that it's extremely effective, but for some patients, it's been effective and it's probably worth trying.”

A person with SCI: "using .. e-stim at my biceps area, that actually helped slightly...it.. kind of ... helps while you're doing it, but then after you're doing it, the pain returns.”

Benefits

- Minimal side-effects
- No prescription needed
- Affordable
- Non-invasive
- Non-painful

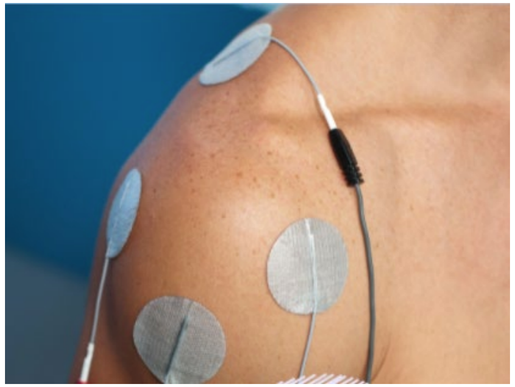

3. How do People Manage Pain: Activity and Exercise

SCI Perspectives: Staying Active

“You have to really push yourself to go out the door and even if you have nothing planned that day go for a roll you know and that really helps, like little things like that.”

“Um, a lot of it has to do with being socially active. That you're out and about and not sitting at home thinking about it. And I think it's very important to stay busy. Um, don't have idle time. Don't sit around by yourself and moping. Get out there and do things. Uh, stay active. Be with people. Uh, don't let the pain dictate what you're going to do. You be proactive and go out and try to live a normal life.”

SCI Perspectives: Exercise

"The physical exercise has a lot to do with how I deal with the pain. And I think I couldn't talk to people enough about that. You got to stay active. You got to exercise. And you got to be outside. Don't sit home and feel sorry for yourself. Just go out there and stay active.”

“Every day a little bit, and try to at least get the pressure off of you. Uh, sometimes it can hurt worse, it depends on what exercise you do. I've learned the exercises that don't make it hurt worse, that actually make it more comfortable.”

“A lot of it has to do with exercise. I exercise every day.”

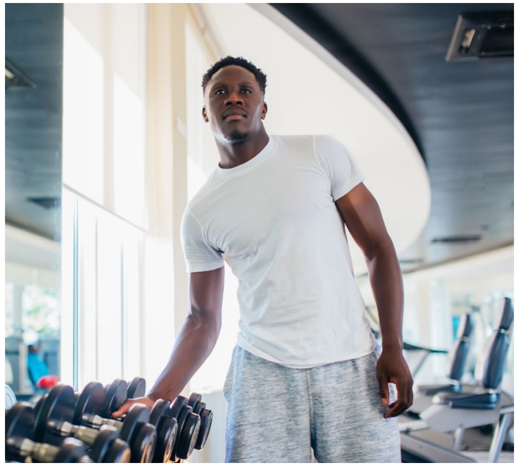

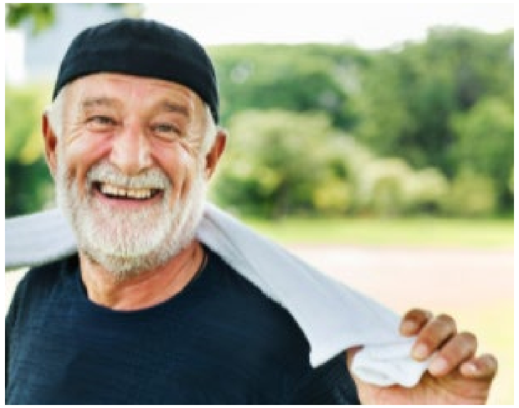

4. Exercise

Exercise Guidelines

The International Scientific SCI Exercise Guidelines (ISSEG) recommends both:

- 20 minutes of aerobic exercise 2 times per week (moderate to vigorous intensity)
- 3 sets of strength exercises for each major muscle group 2 times per week (moderate to vigorous intensity).

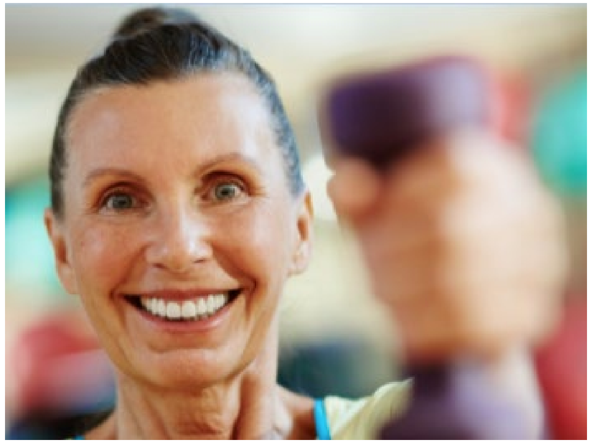

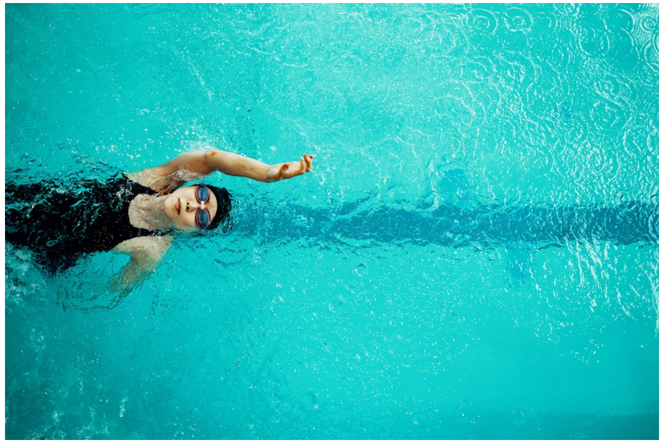

Aerobic Exercises

- Hand cycling
- (arm ergometer)
- Swimming
- Rowing
- Cycling
- Wheelchair pushing
- Walking

Strength Exercises

Arms

- Shoulder press
- Arm Curls
- Lateral Arm Raise
- Shoulder rolls & shrugs

Legs

- Seated knee flexion
- Hip flexion
- Seated hip adduction

5. How do People Manage Pain: Rest and Physical Therapy

SCI Perspectives: Rest

“The best, the best pain relief is just to lie down and I lie on my side, on my left side typically. For some reason, I've developed that habit. With all else fails, I just have to go lie down and then I get some relief.”

“I think a lot of my pain comes from, uh, being in a wheelchair, you know, all the time and sitting in it, and my legs get tight. Um, the front of my legs, my thighs. You know, I lie down in bed and stretch out, and cross my legs, either long way or both ways, left and right. That seems to, to take, to take a lot of the pain away. Sometimes when I lie on my stomach flat on the bed, that seems to help"

SCI Perspectives: Physical Therapy

“So physical or occupational therapy... sometimes depending on ..... if a person is a higher level tetraplegic and their neuropathic pain is around a limb ... like their hand, and they're trying to use it for something it's, a lot of neuropathic pain in that area then...... I will work with the therapist to either do some type of mirror therapy, or desensitization therapy to allow the ... focus on function over just "get rid of the pain."

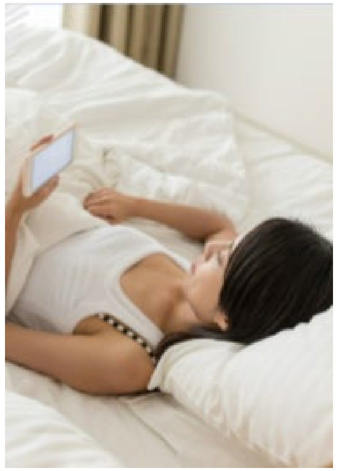

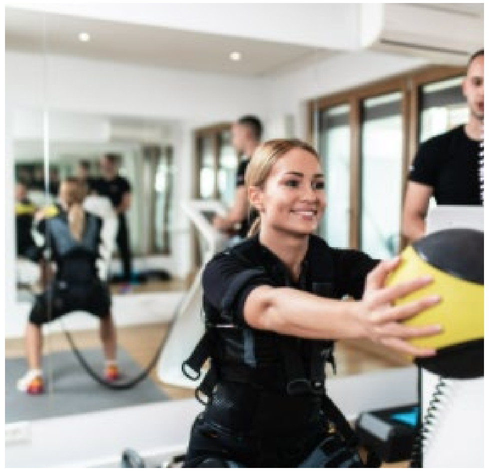

6. How do People Manage Pain: Massage and Acupuncture

SCI Perspectives: Massage

“I gotta tell you a good massage makes a big difference.”

“Sitting helps a little bit, but I really have to get reclined to really get the maximum pain relief. I have a, I have an automated, uh, massage chair, and the massages, they help relax the muscles a little bit. It reclines, and it does your legs, and it does your buttocks, and your back. And if I sit in that chair and recline for a while, then it would generally easy off”

SCI Perspectives: Acupuncture

“I've done acupuncture; that helps for about an hour….. the cold laser therapy, same thing. That'll help for an hour or so, um, with, uh, pain relief.”

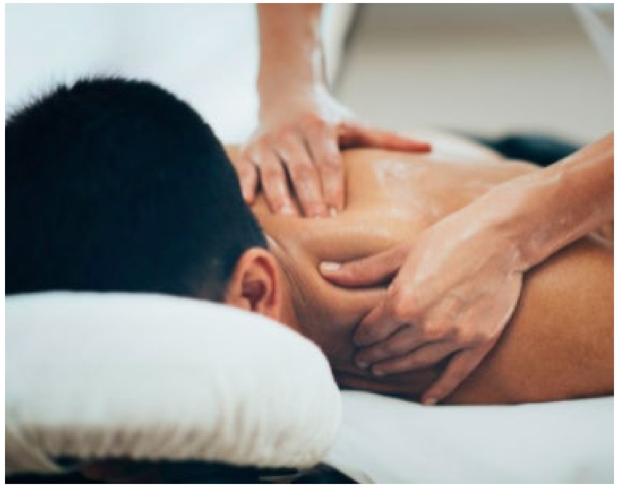

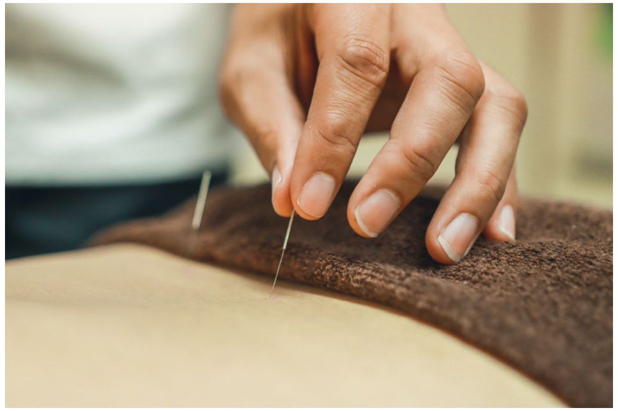

7. How do People Manage Pain: Yoga, Relaxation, Meditation, and Mindfulness

SCI Perspectives: Yoga, Relaxation, and Meditation

“To best handle my pain, I go into a lot of deep meditations. I exercise, do a little bit of, um, yoga, and meditation together, is a good combination. It's natural. When I meditate, I get into my mind really good. It takes me out to a different world where I don't feel pain.”

“So I think it's with, you know, meditation or, um, relaxation, it does help with the pain.”

SCI Perspectives: Mindfulness

“I use my mind as a way to deter pain from breaking me down"

“…I’ll place myself somewhere else, think about something else, take my mind off of the pain and place myself somewhere else. And even when I focus on the pain, move somewhat above the, above the pain before it gets back to, yeah you are, you’re separating yourself from it, you’re not right there, right there with it.”

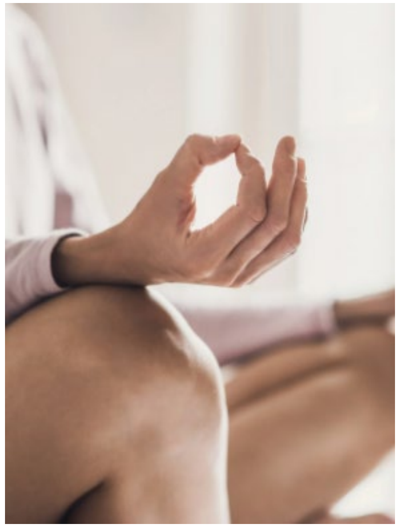

8. Mindfulness & Meditation

Mindfulness is the art of being present. It involves going from one moment to the next with disciplined awareness. As John Kabat-Zinn (1994) describes, it is the "complete owning of each moment of your experience, good, bad, or ugly." In other words, mindfulness means being aware of your own thoughts, feelings, and bodily sensations, while also feeling a sense of calm and peace. This sense of calm and peace will in turn help you to achieve a clearer lens through which you can improve reactions, increase your ability to make conscious decisions and ultimately enhance your quality of life.

There are several ways you can become aware of such thoughts and feelings in order to shift your perspective. Meditation can assist in the mindfulness process by helping you to become more aware of your own thoughts, feelings, and physical sensations. This in turn may help to increase your sense of calm, allowing you to clearly identify your experience, which may result in your ability to make a clearer choice about how to manage your pain. Meditation can also take on several forms of practice, such as learning deep breathing or diaphragmatic breathing, practicing a body scan guided meditation, visualizing yourself being in a safe and comfortable place, or listening to a prerecorded meditation.

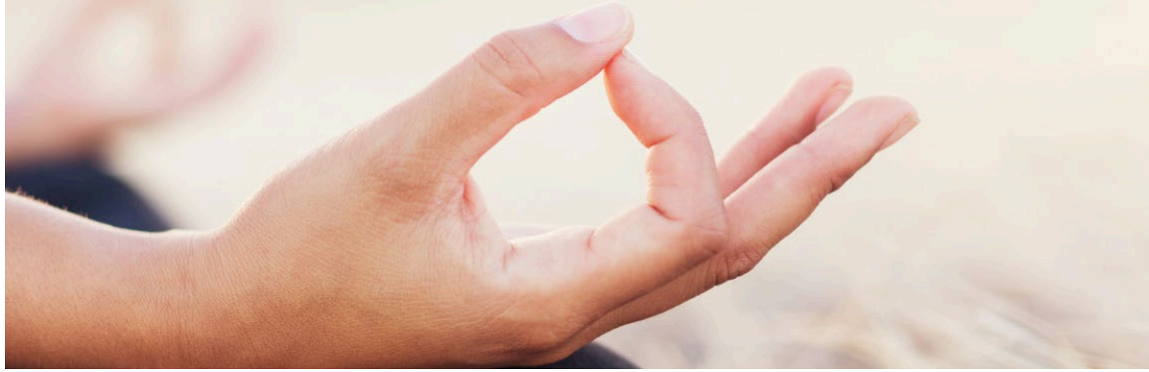

9. How do People Manage Pain: Mental Strength

SCI Perspectives: Mental Strength

“I have found that no matter how much you hurt, you got to keep moving”

“I deal with it, I think, very well. In the 19 years, it's never stopped me from doing anything. I was an athlete, and I still am, prior to my injury, and I think that had a lot to do with my attitude towards the pain.”

“I try to live a normal life and don't let the pain, even though it's with me a lot, I don't let the pain uh, dictate how my life is led.

“You're in pain, sure, but if you look at it as a challenge to get through it, um, where's like a goal, if you will, where you become ... I mean, it's your mission. That's your mission at the end of the day, is to not let it get to you.”

“And if you always have an attitude of gratitude and say to yourself when you start going down that bad path. I say to myself, "Stop, take a deep breath, attitude adjustment. You need an attitude adjustment."

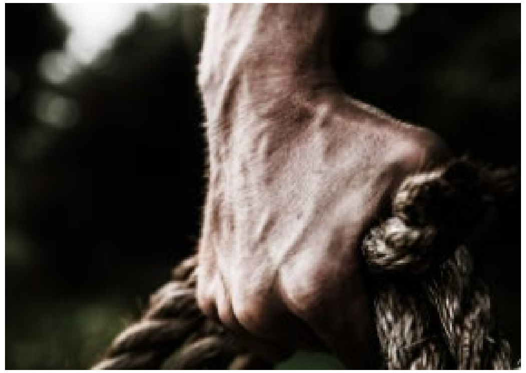

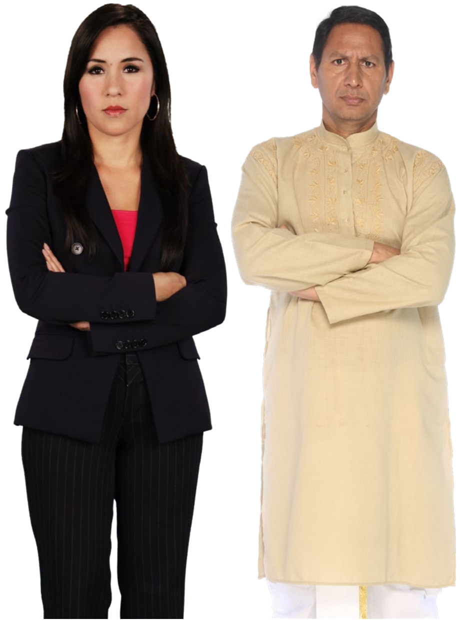

10. How do People Manage Pain: Ignore or Tolerate

SCI Perspectives: Ignore or Tolerate

“If I sit there and I think about the pain it will get worse.”

“I wake up and can’t believe I’m in so much pain and then I tell myself just forget about it, it’s there and it’s there and it is what it is and just forget about it.”

“There are days when it’s really easily tolerable but it’s always there I just put it in back of my mind after you know twenty something years so.”

“I’d rather put up with the pain, than… take the alternative. And… narcotics I don’t… I don’t wanna do any anymore… ever! And uh… I know there are a lot of stuff I could do to self-medicate but… no alcohol either.”

“Yeah like when you have so much pain that I, I get to the point where it's not that I don't care about anything, I f- I just, I'm just trying to make through the day. I'm not negative. I'm not positive. I'm just, I don't know where it is that I go.”

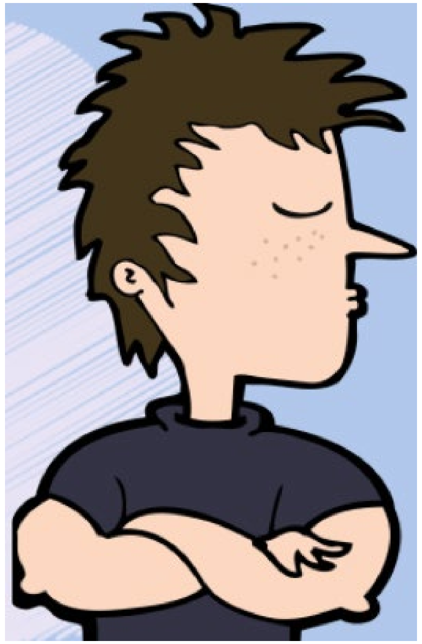

11. How do People Manage Pain: Distraction and Companionship

SCI Perspectives: Distraction

“Anything distracting. I mean, watching TV, sports, anything that gets your mind not on yourself.”

“Cause let's just say, you know, I'm having a really good conversation or I'm watching a really good, like a, uh, IMAX, I'm having a lot of fun at that moment, like, I'm not concentrating on the pain, so it's not that, like, it's not that serious to the point where I'm like, "Oh my gosh. I'm so miserable, oh, like I'm in pain." It's up to the point, like, "Okay, I'm having a good time."

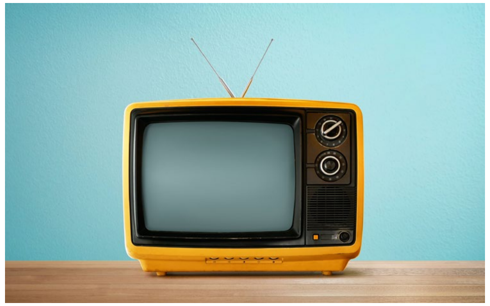

SCI Perspectives: Companionship

“…if they can get someone or something like even pets you know pets are great for that you know and, and just something to distract their mind from the pain you know whatever it could be spiritual, it could be you know a pet like I said or someone in the family but um that was one of my best um pain medicines was having companionship.”

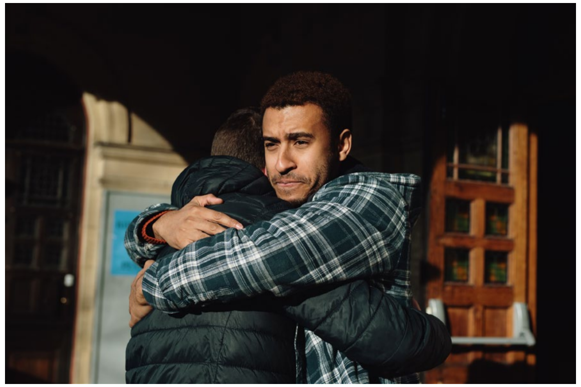

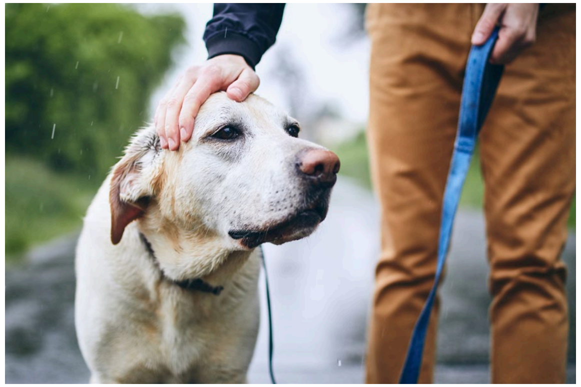

12. How do People Manage Pain: Spirituality, Hope, and Optimism

SCI Perspectives: Hope and Optimism

“Stay happy; find anything that makes you happy. I don’t care if its knitting, I don’t care if it’s taking pictures. You have to fight it like it’s, your life depends upon it because that pain, it will come and it will knock really hard. But if you find, like I watch America’s got talent, I be in so much pain but I put that on cause I hearing their inspiring stories you know. Their struggles you know we all got a struggle… “

“I think about ... Pain is a challenge more than a problem.”

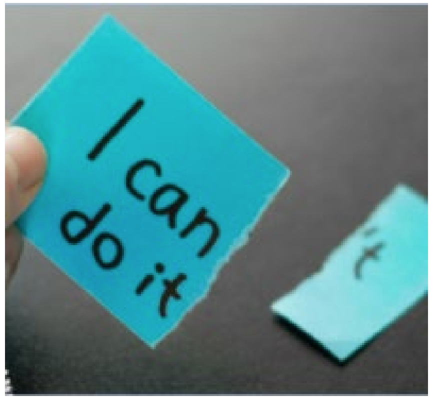

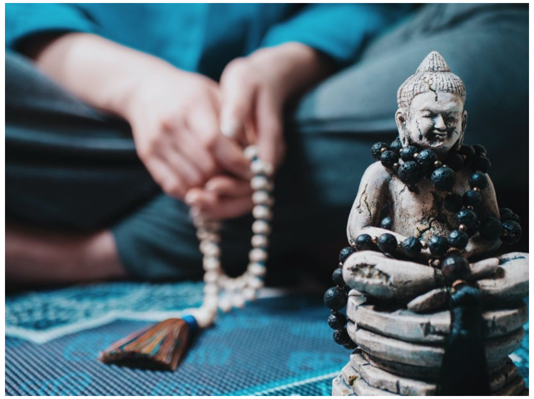

SCI Perspectives: Spirituality

“Well, pray is the best thing. Uh, I try to pray every morning and that helps me get through with the pain, uh, to deal with it. Um, I feel better in the mornings after I've rested, so, you know, I get started there in the day and then as the pain begins to ratchet up, I usually take something... but I'm usually in a state of trying to pray. Instead of just meditating on the universe, I'm usually in prayer. Um, and that helps me cope with it. And understanding that other people are in a lot worse situation than I am and being thankful for what I do have, bring this perspective to what has happened to me and believing that God has some purpose for this in my life. It's not just some random act of the cosmos. There is some purpose one day that I'll be able to realize.”

13. Pain Acceptance, Not Resignation

Many individuals living with spinal cord injuries struggle with the idea of accepting their pain and/or disability. The thought of accepting something perceived as negative or unwelcoming may seem counterproductive, or perhaps it may feel as if you are just giving up because you believe there is nothing else that can be done about it, yet this is not what defines acceptance.

Unlike resignation, which is when you have convinced yourself that nothing more can be done about your situation, acceptance means you recognize the reality of your situation, while still acknowledging your sense of agency.

In other words, acceptance is not a passive state like resignation, where you simply “throw in the towel”, but rather it is a state where you can deliberately commit to acknowledging that your situation has changed, but where you also acknowledge that your situation will no longer have power over you.

Through acceptance, you become capable of creating change, and will no longer feel trapped, whereas through resignation you remain a passive bystander believing that the odds are always stacked against you.

SCI Perspectives: Pain Acceptance

“Won't let the pain dictate how I'm living my life. But it's something I live with. You, you have to learn how to deal with it or it'll beat you every time.”

“If you don't face it, and it comes out years later, you're really gonna be in trouble. I faced mine”

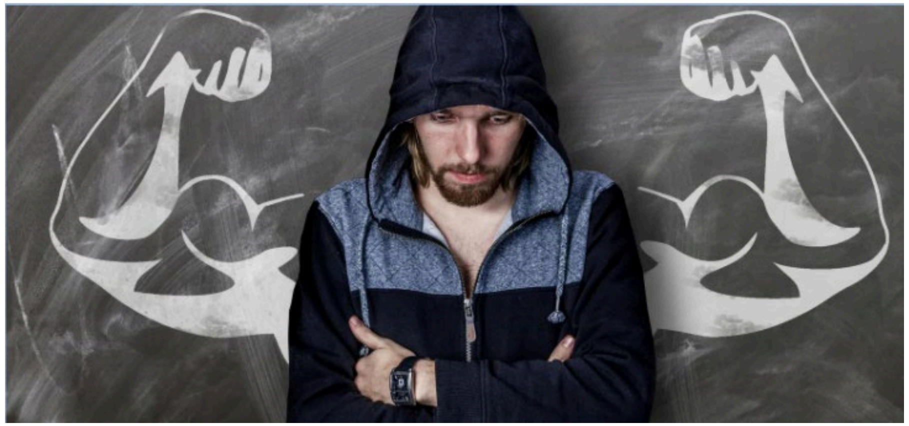

The pains that occur after SCI vary widely in where they are felt, what they feel like, how they respond to treatments, and how they affect your quality of life.

14. Learning, Planning, and Anticipating

SCI Perspectives: Learning, Planning, and Anticipating

“Pain to me, is, is uh ... Is something that an individual has to educate themselves on how to tolerate. And, uh, they can only educate themselves if the material is presented, or if they're allowed access to educational materials in regards to how to cope with their pain."

“Everyday is a challenge. Um. It's a learning experience, for me. It's been a learning experience. And um, it teaches not to take things for granted.”

“How do I turn so that this side won't hurt anymore? This side is hurting, let me turn, how do I stretch my foot? Let me see how I can wiggle my toe. You know, just, I'm always learning and then once my body starts to feel aching or I start to, "Okay, let me learn to calm down, relax, give my body a break, go back to ... To square one in a couple minutes, or in an hour, or maybe tomorrow." You learn to have patience and you learn to analyze everything you do And if I can't focus on something, I leave it alone, I don't torment myself with it. I leave it alone and I start another day when I'm more focused.”

“Um, just, uh, I don't overdo it. I learn that, not to overdo it, cause that hurts.”

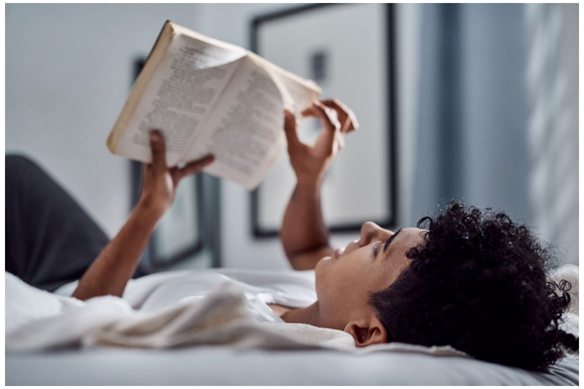

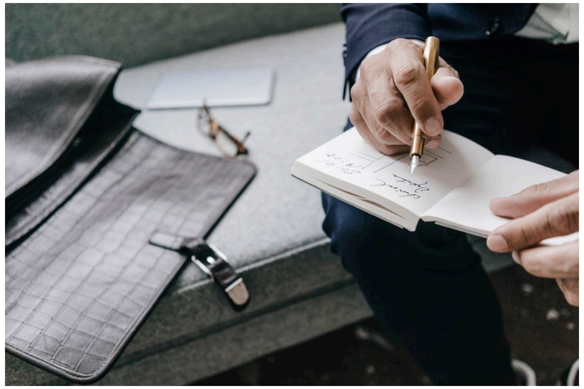

15. Avoiding Triggers

One important part of managing your pain is to figure out what factors and situations can make your pain worse. These are some factors and situations that people with SCI have reported in a study involving 120 participants. Please note that these factors do not have the same effect on all people with SCI.

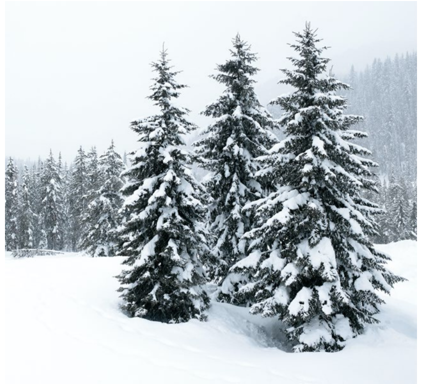

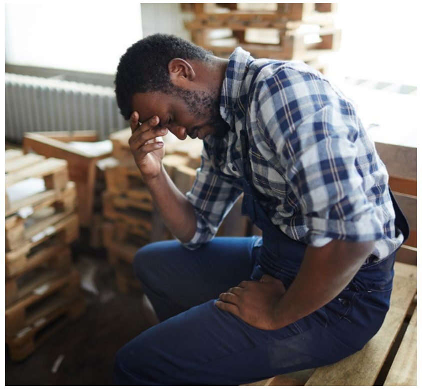

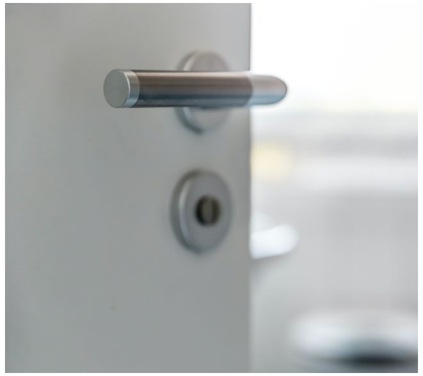

Situations that worsen their pain:

- Prolonged sitting without changing position
- Infections (e.g., UTI)
- Fatigue
- Muscle spasms
- Cold weather
- Sudden movements
- Negative mood (e.g., feeling sad or anxious)
- Constipation
- Full bladder
- Voluntary physical activity. Some people feel better when they exercise, and some people feel worse. Since exercise has so many health benefits it is important to figure out what type of exercise works best for you.

16. Anxiety and Depression: Normalizing the Experience

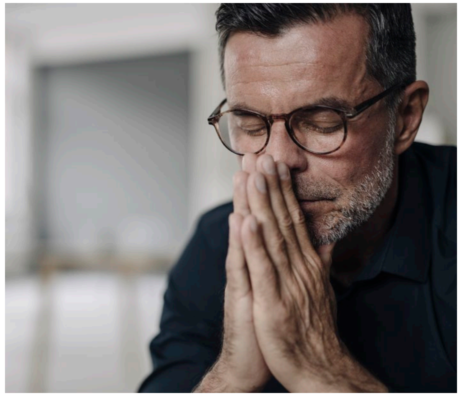

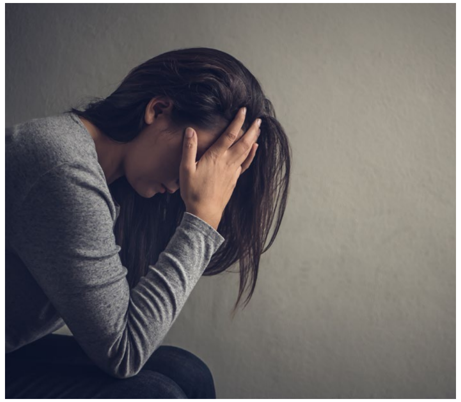

Being told you must learn to live with pain should not be the end of the road, but the beginning. Many individuals living with SCI and chronic pain may feel discouraged and experience depression or anxiety because of chronic pain that feels like it is unmanageable.

These feelings are very commonly experienced by individuals who experience chronic pain and disability, and they are also very treatable mental health conditions.

Treating the psychological symptoms of emotional and physical pain is a critical part of your recovery and overall well-being.

Psychological conditions can be treated by seeking psychotherapy treatment with a mental health provider. You may learn about cognitive-behavioral strategies to reduce your symptoms of emotional distress, as well as learning about mindfulness strategies.

You can learn to develop skills that may help you to effectively manage the psychological side-effects of living with spinal cord injury and chronic pain, which can ultimately result in optimizing your overall quality of life.

17. Significant Others, Family Members, & Caregivers

It can be challenging to watch a loved one suffer, let alone suffer from chronic pain which can be difficult to soothe and manifests in a variety of ways. Caregivers can be left feeling powerless and stressed. Yet, caregivers are a crucial part of the persons' support system as well as the health system.

Therefore, taking care of caregivers becomes an important task in taking care of our loved ones suffering from chronic pain. You, the caregiver, are the backbone to your loved one's care as you stay in the background, seldom complain, and juggle a variety of responsibilities. Feelings of helplessness when we see our loved ones in pain can increase our stress response which can lead to symptoms such as diminishing self-esteem, irritability, and loss of focus on your own life.

Perhaps you feel that you have less personal time, feel isolated from your own social support system, and experience an increase in worry. What can be done about this array of emotions?

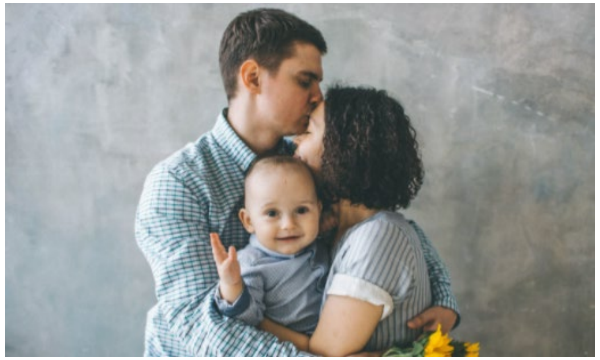

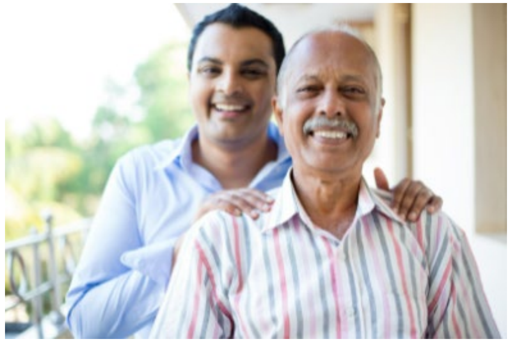

17. Significant Others, Family Members, & Caregivers

The following steps are ways that you, the caregiver, can keep building your resilience, your well-being, and remind yourself that "you can do it!"

Ask for help: Help can come in a variety of ways such as a talk with a friend, or a series of talks with a professional (known as psychotherapy), or if finances allow, getting some help to divide your workload. Resources such as support groups are also available to you to help you share your feelings with others who just might be experiencing your exact thoughts and emotions. Family or group psychotherapy is another option available to you and your loved one to help mediate any challenges in your relationship.

Seek out your social support system: Research has shown that not just high quality, long visits, improve our well-being, but short and frequent visits with others will also lower our stress levels. Reach out to friends, family members, and other individuals who will provide you with positive moments.

Get out of the house: Arrange to leave your home once a day whether that is to take a walk, go to the grocery store, spend time alone or spend time with friends. Make sure to also obtain sunlight on your skin throughout the day. Research has also shown that sunlight (and vitamin D) has a significant impact on your mood and stress levels. Aim for 30 minutes of sunlight per day and perhaps include your loved one in this adventure if suitable.

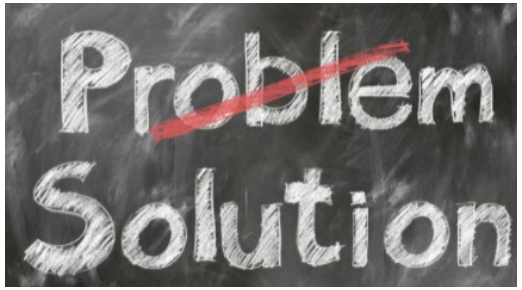

17. Significant Others, Family Members, & Caregivers

The following steps are ways that you, the caregiver, can keep building your resilience, your well-being, and remind yourself that "you can do it!"

Ground yourself: *Learning to meditate, and taking time to do so, can also help re-center you. Perhaps taking 15 minutes each day to meditate, or to simply sit out in your garden and breathe in the fresh air, allowing yourself to be fully present at the moment, can help re-energize you. Try and make this a daily practice each morning, or whenever you choose.*

Keep a gratitude journal: *Make a habit of writing 3 things you are grateful for each day. It may be as simple as spotting a bird in your garden or enjoying a cup of coffee. Research has shown that focusing on at least 3 things each day that we can be grateful for helps to improve mood.*

Be Mindful of each moment: *Allow yourself to not become overwhelmed by thoughts of the future, or by past events, but rather stay focused on the here and now. This will help reduce unnecessary worries that get created by living in the imagined future and will help you see that if you stay present in the moment, what you are feeling in this moment will pass. Remember that the only permanence in life is change itself.*

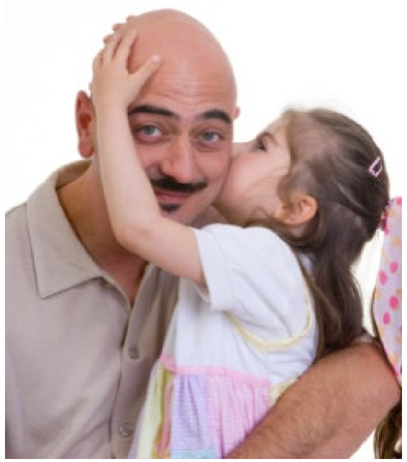

19. Final Message

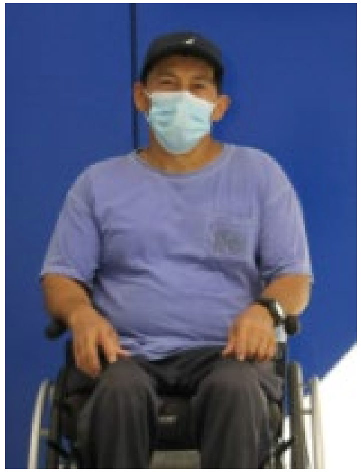

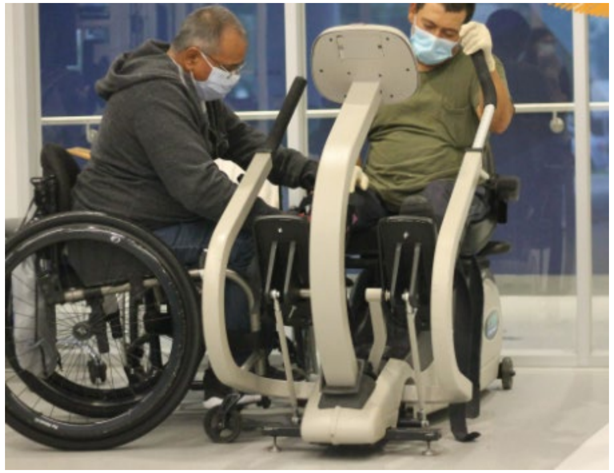

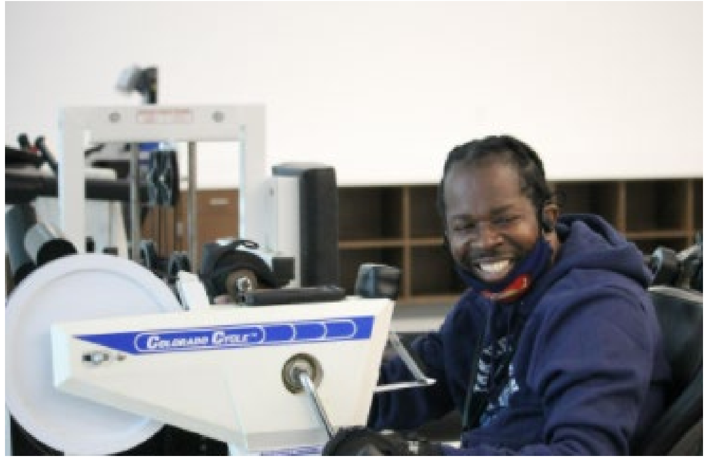

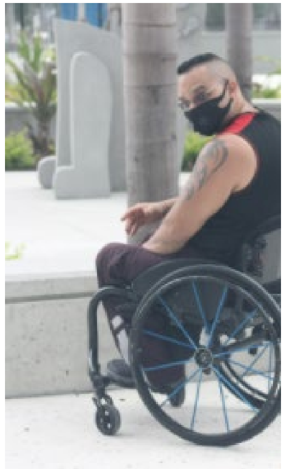

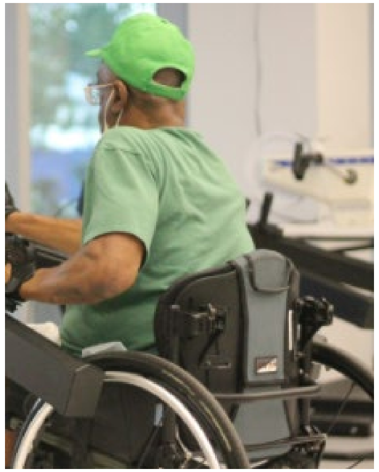

"Like I said you have to do what works for you. Take in the knowledge and then do what works for you."

But remember that...

YOU ARE NOT ALONE!

QUESTIONS?

OR COMMENTS
